# Signal Amplification in the HPT Axis—Evidence for Its Existence, Location, Significance, and Molecular Mechanisms

**DOI:** 10.1111/apha.70202

**Published:** 2026-03-20

**Authors:** Li Jing, Sarahna A. Moyd, Qiang Zhang

**Affiliations:** ^1^ Department of Toxicology and Hygienic Chemistry, School of Public Health Capital Medical University Beijing China; ^2^ Gangarosa Department of Environmental Health, Rollins School of Public Health Emory University Atlanta Georgia USA

**Keywords:** negative feedback, signal amplification, thyroid hormone, TRH, TSH, ultrasensitivity

## Abstract

Thyroid hormones (THs) are under negative feedback regulation via the hypothalamic–pituitary‐thyroid (HPT) axis. How this axis operates to keep the circulating THs within a narrow physiological range is not well understood quantitatively. Led by the design principle of robust homeostatic feedback control, here we review and synthesize the literature under a unifying theme of signal amplification in the HPT axis, providing evidence for its existence, location, functional significance, and potential molecular mechanisms. Drawing on human studies of the circulating TSH‐T4 relationship, we assert that a signal amplifier exists in the brain, where the TH feedback signal is amplified to inhibit TRH and TSH. With mathematical models we illustrate that placing the signal amplifier of the HPT feedback loop in the brain, not in the thyroid, provides an evolutionary advantage, which minimizes the disruption of operating TH levels by possible perturbations. We review the molecular neuroendocrine literature to reveal how signal amplification (ultrasensitivity) is likely achieved mechanically in the hypothalamus and anterior pituitary. We identify multiple signaling pathways in the TRH neurons, β2‐tanycytes, and thyrotropes that mediate the feedback action of THs, including transcriptional and posttranslational regulations of the synthesis, maturation, degradation, and release of TRH and TSH. Collectively, these multistep regulations amplify T3 signal, providing a high feedback loop gain for robust TH homeostatic control. The nature's design principle revealed here enhances our cross‐scale understanding of the systems biology of the HPT axis as a dynamical control system, which can promote precision thyroid medicine and risk assessment of thyroid‐disrupting chemicals.

## 1 Introduction

Thyroid hormones (THs) are iodine‐containing small‐molecule hormones synthesized and secreted by the thyroid gland, comprising primarily thyroxine (T4) as the prohormone and triiodothyronine (T3) as the active hormone. They are essential for the normal operations of various biological systems and processes, including development, cardiovascular, bone, and liver functions, reproduction, metabolism, and energy homeostasis [1, 2, 3, 4, 5, 6, 7, 8]. The circulating free T4 (fT4) and free T3 (fT3) levels are strictly controlled within a narrow 2–3 fold physiological range in human populations [9, 10]. In a healthy individual, the degree of fluctuation is expected to be much smaller, potentially centering around an operational setpoint that suits the individual's physiological state [11]. Out of the normal range, clinical hyper‐ or hypothyroidism may manifest. The circulating TH levels may be altered by a variety of persistent or transient factors and conditions, including insufficient or excessive dietary iodine uptake, thyroid disrupting chemicals, genetic disorders, and autoimmunity [12, 13, 14, 15]. It is imperative that the thyroid system regulate the synthesis, secretion, metabolism, and even actions of THs to maintain robust homeostasis against these perturbations; otherwise a diseased state may arise. Simultaneously it also needs to adjust in response to the body's changing allostatic demand. Toward these ends, multiple global and local mechanisms have evolved, forming a hierarchy of TH regulation. These mechanisms include (i) the classical negative feedback regulation through the hypothalamic–pituitary‐thyroid (HPT) axis, (ii) buffering of free THs by the TH binding proteins (THBPs) in the blood circulation, and (iii) local feedback and feedforward regulations of THs within target tissues by deiodinases (DIOs) and other relevant metabolic enzymes [16, 17, 18, 19, 20].

**FIGURE 1:**
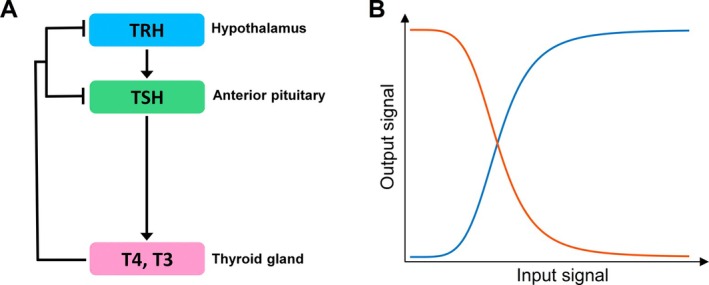
(A) Schematic illustration of the HPT negative feedback loop. Pointed arrowhead: Stimulation, blunted arrowhead: Inhibition. (B) Stimulatory (blue) and inhibitory (orange) ultrasensitive responses presenting as sigmoidal curves on dual‐linear scale.

The long‐term systemic TH homeostasis relies primarily on the classical negative feedback loop (NFL) regulation of the neuroendocrine HPT axis (Figure 1A) [21, 22]. The parvocellular neurons in the hypothalamic paraventricular nucleus (PVN) synthesize thyrotropin‐releasing hormone (TRH) and project to the median eminence (ME), where TRH is released and transported via the portal capillaries to the anterior pituitary (AP). TRH stimulates the thyrotropes in the AP to synthesize and secrete thyroid‐stimulating hormone (TSH) into the systemic circulation. Upon reaching the thyroid gland, TSH stimulates the synthesis and secretion of T4 and T3. This sequential TRH➔TSH➔THs action comprises the stimulatory arm of the NFL of the HPT axis. Circulating THs then reach the brain tissue through cell membrane transporters such as MCT8/OATP1c1 and through transthyretin in the choroid plexus [23], where T4 is converted into T3. T3 inhibits the synthesis, maturation, and release of TRH in the hypothalamus, as well as the synthesis and release of TSH in the AP [24, 25, 26]. These negative actions of THs on TRH and TSH collectively comprise the inhibitory arm of the NFL.

To maintain the circulating TH levels within a narrow physiological range, some quantitative signaling properties are required for the NFL of the HPT axis. In general, for a negative feedback system to achieve near‐perfect setpoint control, it requires that the feedback have a high loop gain unless the feedback is integral [27, 28, 29, 30, 31, 32]. A high loop gain can be realized through signal amplification, which is defined as the following: a small percentage change in the level of the input signaling molecule is amplified to produce a larger percentage change in the level of the output signaling molecule. Be noted that this percentage amplification is conceptually different than the abundance or concentration amplification also encountered in biology literature, where a molecular species existing in a low molar concentration range regulates changes in another species existing in a much higher molar concentration range. The terminology for percent‐wise biochemical signal amplification is *ultrasensitivity*, or cooperativity as often referred to in the literature, which can be achieved by multiple signaling motifs and is often empirically described by Hill function [33, 34, 35]. Visually, an ultrasensitive response, either stimulatory or inhibitory, presents as a sigmoidal input/output relationship on a dual‐linear scale, and signal amplification occurs on the steep segments of the curves (Figure 1B).

A high loop gain can be achieved by having signal amplification concentrated in either the stimulatory or inhibitory arms of the NFL or distributed evenly through both arms [30]. For the HPT axis specifically, the amplifier can be located within the brain, that is, the hypothalamus and AP, to amplify the inhibitory T4/T3 signal, and/or within the thyroid gland to amplify the stimulatory TSH signal. From an endocrine systems biology perspective, the location of the HPT signal amplifier is critically important, which can have significant implications for TH homeostatic control and biological fitness. The goals of this article are to (i) review the scientific evidence supporting that the location of the HPT signal amplifier is in the brain, (ii) illustrate, with the aid of simple mathematical models, that placing the amplifier in the brain not in the thyroid gland is a nature's design principle that is evolutionarily more advantageous and robust against frequent, undesirable perturbations, and (iii) synthesize the molecular neuroendocrine literature on the signal transduction and gene regulatory pathways in the hypothalamus and AP that function as potential ultrasensitive signaling motifs to collectively amplify the inhibitory actions of T3 on TRH and TSH.

## 2 Anatomical Location of Amplifier and Feedback Gain—Insight From the TSH vs. fT4 Relationship in Humans

### 2.1 The Inverse TSH‐fT4 Relationship

**FIGURE 2:**
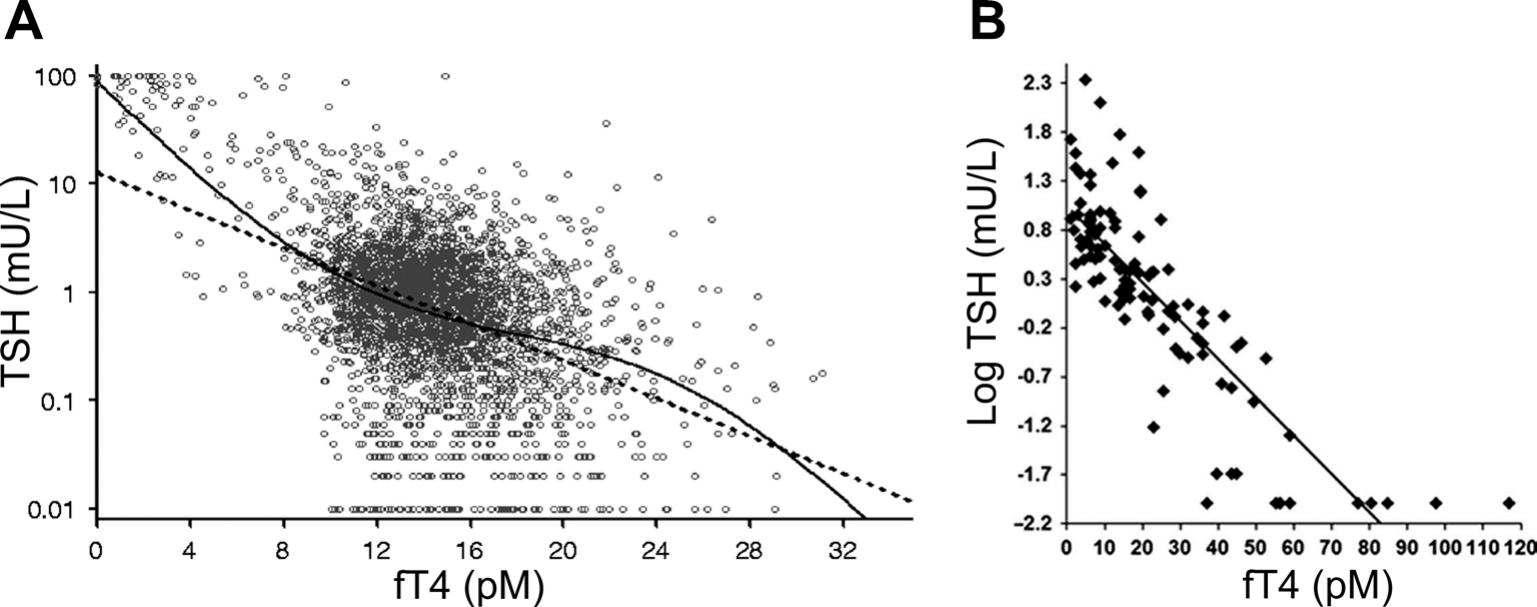
The inverse relationship between logTSH and fT4 in humans. (A) LogTSH and fT4 levels in 3223 Germans [36]. Dashed line: Linear fitted curve, solid line: A nonlinear fitted curve based on the error function. Image was adapted from Hoermann et al. [36] with permission from Oxford University Press. (B) LogTSH and fT4 levels in 109 blood samples received at the NIH Clinical Center where fT4 was measured by LC–MS/MS [37] fitted with a linear function. Image was adapted from van Deventer et al. [37] with permission from Oxford University Press.

In human populations, circulating TSH and TH levels vary across individuals. The reference range of TSH spans more than one order of magnitude, whereas that of fT4 or fT3 is much narrower, spanning about 2–3 fold [9, 10]. Despite this contrast, it has long been established that the TSH and fT4 levels exhibit an inverse relationship (Figure 2A) [36, 37, 38, 39, 40, 41, 42, 43, 44, 45]. This inverse correlation appears even stronger when fT4 is measured by using plasma ultrafiltration followed by liquid chromatography–tandem mass spectrometry (LC–MS/MS) (Figure 2B) [37, 42]. The inverse relationship seems to be conserved under different biological conditions, including gender, time of the day, life stage (except pregnancy), and in some disease conditions such as non‐HPT related cancers [41, 42, 46].

### 2.2 The logTSH‐fT4 Relationship

The TSH‐fT4 relationship in humans also has important quantitative features. Many early studies showed that the relationship could be log‐linearly approximated, where logTSH is inversely associated with fT4 in a linear fashion [37, 38, 42, 44, 47]. More recent studies challenged this simplified view, arguing that the slope can be different on different segments of the curve. Using clinical data of patients with thyroid conditions, Hoermann et al. found that the inverse relationship between logTSH and fT4 can be better described by a nonlinear curve with 3 distinct segments [36]. The middle segment covering most of the fT4 reference range has a slope that is less steep than the two outer segments that extend to the hyperthyroid and hypothyroid regions. Clark et al. reported a similar trend of slope change in both men and women > 65 years old by using a 4th‐order polynomial function [41]. The logTSH‐fT4 relationship in a community‐based cohort was significantly better fitted with a number of nonlinear models which all have a similar trend of slope change as that reported by Hoermann et al. [43]. Using a large sample of 152 261 subjects collected over 12 years, Hadlow et al. found that the logTSH‐fT4 relationship can be best described by two overlapping negative sigmoid curves [39].

While these cross‐sectional studies indicate that the logTSH‐fT4 relationship can be nonlinear, in a study of 13 379 individuals, each having at least six measures of serum TSH and fT4 levels at different times, Rothacker et al. reported that most of the intra‐individual TSH‐fT4 relationships are better fitted with log‐linear functions than nonlinear functions (including polynomial up to the 4th‐order and sigmoid function), especially in those with > 10 measurements [40]. Geode and colleagues also achieved a good fit for TSH and fT4 using exponential functions (equivalent to log‐linear) in a small study of thyroid patients [48] and a further improved fit to thyroidectomized patients supplemented with L‐T4 when the negative feedback from fT3 to TRH is included in their model [49]. Log‐linear associations between TSH and fT3 with varying slopes were also reported in individuals whose fT3 was above the upper reference level resulting from exogenous T3 administration [50]. Moreover, in contrast to the above‐mentioned cross‐sectional studies [36, 41], Rothacker et al. found that the slopes of individuals within the mid‐reference range of fT4 were the highest, while the slopes in those falling on either the low or high ends of fT4 were less steep [40]. That the TSH‐fT4 relationship is log‐linear in individuals but with differential slopes across the fT4 range may reconcile with the nonlog‐linear appearance observed at the population level as individuals are aggregated to form the population average.

The shape and position of the TSH‐fT4 relationship in individuals are subject to modulation by many factors. Some of the variations of TSH and fT4 could be attributed to the time of the day at which samples were collected, hysteretic recovery of TSH after thyrotoxicosis normalization, and laboratory measurement and reporting uncertainties, according to an analysis by Goede and Leow [51]. Genetic, epigenetic, age, sex, circadian rhythm, dietary iodine, and environmental factors may all contribute to the interindividual and intraindividual variations and therefore influence the TSH‐T4 relationship [39, 41, 43, 52, 53, 54].

### 2.3 Amplifier Location and Feedback Gain in the HPT Axis

In theory, interindividual variabilities in TSH and fT4 levels can be caused by biological and environmentally related variations in all organs and tissues that participate in regulating the HPT axis, including the hypothalamus, AP, thyroid, and those responsible for hormone metabolism and clearance. The question is which organ(s) contribute the most variabilities. Given the negative feedback nature of the HPT axis, where TSH stimulates T4 and T4 inhibits TSH, the inverse relationship between the two strongly suggests that their interindividual variabilities must originate *mainly* from variations in the processes that *directly* alter fT4, rather than TSH [55]. Therefore, when fT4 is decreased (or increased) as the initiating event, it causes TSH to increase (or decrease), resulting in an inverse relationship between the two hormones. Such initiating events can impinge in the thyroid gland to alter T4 synthesis and secretion, and/or in extrathyroidal organs such as the liver to alter T4 metabolism and clearance. Although variations at the hypothalamus and AP certainly contribute to the interindividual variabilities of TSH and fT4, they are unlikely to be the main source. Were this the case, a rising (or declining) TSH level would have caused an increase (or decrease) in the fT4 level, resulting in a positive relationship, as would occur during allostatic regulation, for instance, in the case of concurrent rising of fT4 and TSH driven by body composition changes through leptin [56]. Likewise, a positive relationship has been observed between adrenocorticotropic hormone (ACTH) and cortisol in the hypothalamic–pituitary–adrenal (HPA) axis in human populations, an endocrine system that is often under allostatic regulation [57, 58].

As the circulating fT4 varies within a range of 2–3 fold, TSH varies in the opposite direction by more than two orders of magnitude in humans (Figure 2). Quantitatively, this speaks of signaling amplification in inhibitory sense—a small percentage increase (decrease) in fT4 levels results in a much larger percentage decrease (increase) in TSH levels. Given that the negative feedback action of T4 on TSH occurs in the brain, this amplified inhibition strongly suggests that the signal amplifier in the HPT axis must be in the hypothalamus and/or AP, not in the thyroid. Were it located in the thyroid, the degrees of variations of fT4 and TSH would have been comparable, that is, they would vary in a similar fold range albeit still in opposite directions, according to control theory [30].

Mathematically, when fT4 doubles and TSH correspondingly decreases by a certain fold, say X, the logarithmic gain *n* = log_2_(X). For instance, when X = 64, *n* = 6. Several human studies suggest that the aggregate logarithmic gain of the amplifier in the brain may range between 5 and 7. In a cross‐sectional study involving over 500 people, on the main slope of the logTSH‐fT4 index (FT4I) curve, a doubling of the serum fT4 level was associated with an approximately 160‐fold reduction in serum TSH level, suggesting n is slightly > 7 [38]. In two studies using LC–MS/MS to measure fT4, when the fT4 level varied within 2‐fold, TSH exhibited a > 100‐fold range [37, 42], suggesting *n* > 6. In another study where the average steepness of the logTSH‐fT4 curve is the highest around the median fT4 levels, TSH changed by about 50 fold when fT4 varied by 2‐fold, suggesting 5 < *n* < 6 [40].

Within individuals, where free THs and TSH fluctuate much less than the population reference ranges [11], the steepness of the logTSH‐fT4 curve may be even more pronounced. In a study where individuals received T4 to suppress TSH and had reached new steady states, the slopes of the TSH‐FT4I curves in some were steeper than the aggregate slope, corresponding to about a 300‐fold reduction in serum TSH for a doubling of FT4I, suggesting the amplifier's gain in individuals can be even greater than estimated using population data which may obscure and misinform the individual slopes [38]. In hypothyroid patients receiving T4 therapy, multiple intra‐individual measurements of fT4 and TSH also revealed similar or even greater gains in the lower ranges of individual TSH‐fT4 curves [48, 59].

Animal studies also showed that T3 inhibits TRH and TSH in an amplified manner. In hypothyroid male mice, i.p. injection of T3 or Sob‐AM2, a CNS‐penetrating prodrug of thyromimetic sobetirome, can produce nearly switch‐like dose response of hypothalamic TRH mRNA inhibition in 6 h [60]. In the same study, T3 and sobetirome can also induce steeply sigmoidal inhibition of TSHβ mRNA in the pituitary. Taken together, it appears that the T3 signal is amplified to inhibit TRH and TSH transcription.

In summary, the overall relationship between the circulating TSH and fT4 levels in a human population has several key features: (i) they are inversely associated, (ii) the inverse relationship can be quantified largely as log‐linear or in some nonlinear forms, and (iii) small fractional changes in fT4 are inversely associated with much greater fractional changes in TSH. These features, along with animal evidence, provide critical insights into the signal amplification scheme within the HPT axis, suggesting that the amplifier is located in the brain where it amplifies the negative feedback signal of T4 to inhibit TSH production and/or release. In contrast, the TSH‐stimulated signal transduction and gene regulatory networks in the thyrocytes do not appear to be conducive to signal amplification [61], and there is no evidence suggesting that T4 and T3 production and secretion respond to TSH in an ultrasensitive manner.

## 3 Ultrasensitive Mechanisms of T3‐Mediated Signal Amplification in the Hypothalamus and Anterior Pituitary

As argued above, a centrally located feedback signal amplifier is expected to exist in the HPT axis; however, the nature of this amplifier, including its molecular composition and exact locations, is largely uncharacterized. To amplify biochemical signals percentage‐wise, molecular ultrasensitive response motifs (URM) are required [33, 34, 35, 62]. At least six URMs representing unique molecular interactions have been identified, including positive cooperative binding, homomultimerization, multistep signaling, molecular titration (or sequestration), saturable covalent modification cycle, and positive feedback. When embedded in appropriately structured biological circuits, these URMs provide essential signal amplification enabling cellular fate decision‐making via multistability, homeostasis, and biological rhythm [28, 33]. In this section, we set out to review the molecular neuroendocrine literature on the signal transduction and gene regulatory pathways in the hypothalamus and AP and synthesize the findings to assert that multiple URMs operating collectively serve as the potential biochemical amplifier that mediates the inhibitory actions of T3 on TRH and TSH.

In the brain, T4 needs to be converted to T3 to inhibit the transcription, synthesis, posttranslational maturation, release, stability, and action of TRH and/or TSH [21, 63, 64, 65]. The inhibitory actions of T3 are mediated via the nuclear thyroid hormone receptors (TRs). TRs are encoded by two genes, *THRA* and *THRB* [66, 67]. The *THRA* locus encodes mainly TRα1 and TRα2 through mRNA alternative splicing, whereas the *THRB* locus encodes TRβ1 and TRβ2 by using different promoters [68, 69, 70]. The TRα1 and TRβ1 isoforms are expressed ubiquitously throughout the body [71]. It is TRβ2, which is expressed specifically in the hypothalamus and AP, that acts as the primary mediator of T3 feedback inhibition [72, 73, 74, 75, 76, 77], however, a contributory role of TRα cannot be completely ruled out [78]. TRα2 does not appear to bind T3 and may inhibit the function of TRs through exerting dominant‐negative activity over TRα1 and TRβ [79]. Heterodimerizing with retinoid X receptor (RXR), TRs exert their genomic action mainly by binding to the TH response elements (TREs) in the promoters of target genes. Unlike most nuclear hormone receptors, TRs can act even in the absence of T3, as aporeceptors [80].

### 3.1 Multistep Inhibitory Actions of T3 in TRH Neurons in PVN

#### 3.1.1 TH Transportation Into the Hypothalamus and Role of DIO2

T4 and T3 in the blood circulation are delivered to the brain through membrane‐bound transporters and transthyretin (TTR). Here TTR is synthesized locally in the choroid plexus, which binds T4 and T3 and is then secreted into the cerebrospinal fluid (CSF) [23, 81]. While the small portion of T3 that comes from the circulation can directly inhibit TRH, the T4‐exerted negative feedback action requires that T4 is first converted to T3 locally by DIO2 [63, 82, 83, 84, 85]. DIO2 is expressed in astrocytes, not in neurons, throughout the brain including the hypothalamus [86]. DIO2 expression is highly concentrated in specialized ependymal cells, known as tanycytes that line the floor and infralateral wall of the third ventricle in the medial basal hypothalamus (MBH) [86, 87, 88, 89, 90]. Via membrane transporters including organic anion‐transporting polypeptide (OATP1C1) and monocarboxylate transporter 8 (MCT8), T4 and T3 in the blood and CSF are taken up by these cells [90, 91, 92]. In tanycytes, T4 is converted by DIO2 to T3, which is then released from the apical side of tanycytes into the CSF for delivery to the PVN [21, 85]. T3 may also be released from the tanycytic end feet in the ME, where it can be taken up by the axonal terminals of the TRH neurons and transported retrogradely to the soma in the PVN to regulate TRH [93, 94]. T3 of tanycytic origin can also reach the AP via the portal vessels. Compared with DIO2 in other brain regions and peripheral tissues, DIO2 in the tanycytes here is not or only modestly regulated by local T4 and T3 [19, 85, 95]. For instance, animal studies show compared with DIO2 in the cerebral cortex, hippocampus, and brown adipose tissues, where its ubiquitination is highly upregulated and thus its abundance/activity is downregulated by T4, DIO2 in the hypothalamus is poorly ubiquitinated and remains largely unresponsive to T4 [96]. Though fasting can lead to upregulated expression and activity of DIO2 in the rat hypothalamus, such changes cannot be altered by T4 [95]. This lack of regulation of DIO2 by THs in tanycytes allows the concentrations of the locally produced T3 to change faithfully in response to T4 taken up from the circulation to regulate TRH.

#### 3.1.2 T3 Inhibits TRH Transcription

**FIGURE 3:**
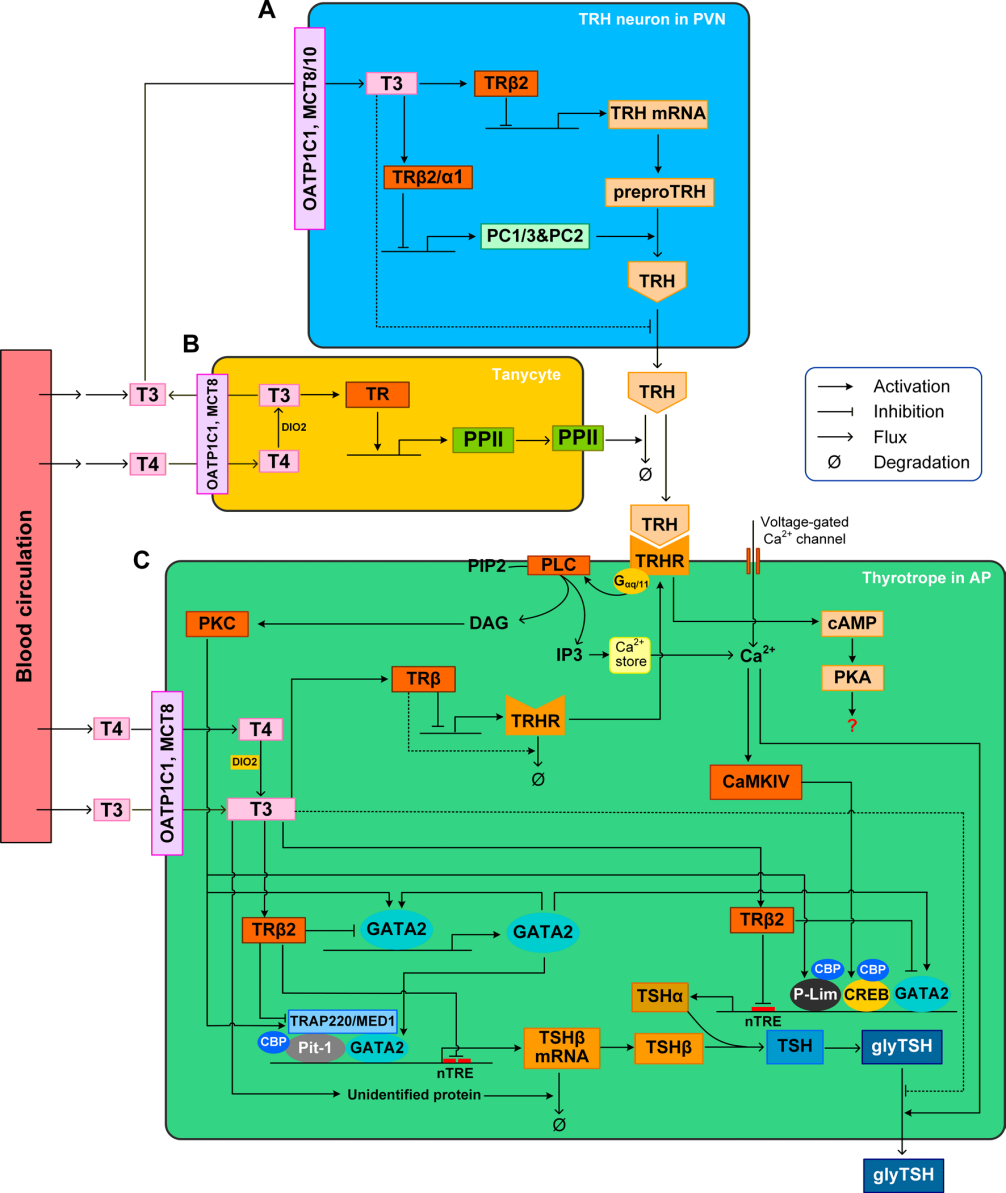
Schematic illustration of the major molecular signaling pathways mediating the negative feedback regulation of TRH and TSH by THs in the hypothalamus and AP. The pathways are composite across species. (A) T3 sourced from the tanycytes in (B) is transported via MCT8/10 and OATP1C1 into the TRH neurons in the PVN, where T3 represses via TRβ2 the transcription of TRH and via TRβ2 or TRα1 the transcription of PC1/3 and PC2 which convert preproTRH to mature TRH. (B) T4 from the blood circulation and CSF is transported via MCT8 and OATP1C1 into the β2‐tanycytes located at the floor of the third ventricle. In the tanycytes, T4 is converted to T3 by DIO2. T3 induces via TR the transcription of PPII, which is then translocated to the cell surface of the tanycyte's end feet in the ME. Surface PPII degrades the TRH molecules released by the axonal terminals of the TRH neurons. T3 produced in the tanycytes is also transported out of the cells and is the main source of T3 reaching the TRH neurons in the PVN in (A). (C) TRH‐stimulated signal transduction and transcriptional induction of TSHα and TSHβ as well as T3‐mediated inhibition of TSH in thyrotropes in the AP. After binding to TRHR, TRH activates Gα_q/11_ which in turn activates PLC to decompose PIP_2_ into DAG and IP_3_. DAG activates PKC which activates (i) TRAP220, (ii) P‐Lim, and (iii) GATA2 to induce the transcription of TSHβ, TSHα, and GATA2 respectively. Through mobilizing internal Ca^2+^ store by IP_3_ and voltage‐gated Ca^2+^ channel, intracellular Ca^2+^ increases to activate CaMKIV which in turn activates CREB to induce TSHα transcription. GATA2 is transcriptionally autoregulated in a positive manner, and GATA2 also induces the transcription of TSHβ and TSHα. TRHR activates cAMP and PKA also but their role in regulating TSH is unclear. Circulating T4 is transported into the thyrotropes via MCT8 and OATP1C1 where T4 is converted by DIO2 to T3 serving as the main source of T3 in addition to the circulating T3. T3 inhibits TSH through several pathways. Through TRβ2, T3 represses TSHβ transcription either by directly binding to two nTREs in exon 1 or via inhibitory interaction with TRAP220. T3 can also destabilize TSHβ mRNA via an unknown protein. Through TRβ2, T3 represses TSHα transcription by directly binding to an nTRE near the transcription start site. Through TRβ, T3 represses TRHR transcription. T3 can also inhibit the release of glycosylated TSH (glyTSH) through an unknown mechanism. Pointed solid arrowhead: Activation, blunted arrowhead: Inhibition, pointed open arrowhead: Flux or conversion, dashed line: Mechanism unknown.

THs negatively regulate TRH gene expression in the PVN neurons (Figure 3A) [97, 98]. Exogenous administration of T4 and/or T3 caused a decrease, whereas hypothyroid conditions caused an increase in TRH expression [83, 99, 100, 101]. The inhibitory action of T3 on TRH transcription is mainly mediated by TRβ2 [102], which is expressed in the TRH neurons in the PVN [103, 104, 105]. In TRβ2‐specific knockout mice, basal TRH mRNA expression, as evaluated with in situ hybridization in the PVN, was significantly elevated and its level was not responsive to either hypothyroid or hyperthyroid conditions [76]. Similarly, hypothalamic injection of siRNA against TRβ2 mRNA also abrogated T3 repression of TRH transcription in mice [106].

Three distinct TR‐binding half‐sites, site 4 (−55 to −60 bp), site 5 (+14 to +19 bp), and site 6 (+37 to +42 bp), have been identified in the human *TRH* gene promoter, which cooperate to mediate T3‐dependent repression of TRH transcription [107]. Site 4 is a negative TRE (nTRE) that can bind both TR homodimer and TR‐RXR heterodimer, whereas sites 5 and 6 can only bind TR monomers. Similarly, it was demonstrated in rat fetal hypothalamic cells that TRβ2 can bind to site 4 and mediate the repressive effect of T3 on TRH mRNA expression [108]. In contrast, in the mouse TRH promoter only T3‐TR monomers are able to bind to site 4 and no monomers can bind to sites 5 and 6, although all 3 sites are required for the repression exerted by T3 [109]. The repression mediated by TRβ2 appeared to involve the recruitment of histone deacetylases [110], which require amino acids 89–116 in the N‐terminal of TRβ2 in humans [111]. Taken altogether, despite there being species differences in the TR forms and TRE sites involved, T3 directly inhibits the transcription of TRH.

#### 3.1.3 T3 Inhibits TRH Posttranslational Maturation

The mature TRH is a tripeptide consisting of 3 amino acid residues (pyro‐Glu‐His‐Pro‐NH2), which is derived from a larger inactive precursor peptide, preproTRH. Rat preproTRH is a 29 kDa protein composed of 255 amino acids, which contains an N‐terminal 25‐amino‐acid signal sequence that targets the protein to the secretory pathway [112, 113]. When the preproTRH enters the endoplasmic reticulum, this signal sequence is first cleaved to produce pro‐TRH [114, 115]. Containing multiple copies of TRH and pro‐TRH‐derived peptides, pro‐TRH is further processed to produce mature TRH [116, 117]. The rat and human pro‐TRH peptides contain 5 and 6 copies of TRH, respectively [112, 118].

Cleaving pro‐TRH into multiple mature TRH molecules requires prohormone convertases (PC), which are key endoproteolytic enzymes expressed in the TRH neurons [116, 117]. Belonging to a family of seven subtilisin/Kexin‐like endoproteases, the principal PCs that regulate the secretory pathways in neuroendocrine cells are PC1 (also referred to as PC3) and PC2 [119, 120, 121]. They are responsible for the cleavage at the C‐terminal side of the paired basic residues flanking the TRH progenitor sequence to produce shorter intermediate peptides [122]. Double in situ hybridization indicated that in the rat PVN, PC2 mRNA is expressed in 60%–70% of the TRH neurons, and PC1/3 mRNA in 37%–46% of TRH neurons [123]. While PC1/3 and PC2 act on overlapping cleavage sites in pro‐TRH, PC1/3 appears to play a more critical role in producing bioactive TRH. This was demonstrated in knockout mice, where T3 was downregulated more significantly when the PC1/3 gene was deleted than when the PC2 gene was deleted [124]. Peptides derived from the N‐ and C‐terminal sides of pro‐TRH can be differentially sorted and secreted, but it is unclear how PC1/3 and PC2 differ in this role [125]. Following the cleavages catalyzed by PC1/3 and PC2, carboxypeptidase E (CPE) removes the two basic residues Lys and Arg on the C‐terminal of the Gln‐His‐Pro‐Gly progenitor sequence [126]. Lastly, the Gly residue is amidated by peptidylglycine α‐amidating monooxygenase and the Gln residue is cyclized to pGlu, yielding the active TRH [117].

The maturation of TRH catalyzed by PCs can be inhibited by T3 (Figure 3A). Using transiently transfected cell models, early studies showed that T3 can repress the human PC2 (hPC2) promoter activity via TRα1 or TRβ1 through binding to nTREs [127]. Subsequent in situ hybridization studies in the rat brain demonstrated that hypothyroid conditions upregulated and hyperthyroid conditions downregulated PC1/3 and PC2 mRNA expression in the PVN [128, 129]. For human PC1 (hPC1), the downregulation of its promoter activity by T3 required two putative nTREs located in separate promoter regions [128], while for hPC2, the more distal nTRE appears to be more important in mediating the inhibition by T3 [129]. In the presence of T3, TRα1/RXRβ heterodimers can bind to these putative sites. In rats, PC1/3, PC2, and TRH peptides were upregulated in the TRH neurons in the PVN in propylthiouracil (PTU)‐induced hypothyroid conditions [130, 131]. In summary, through repressing the transcription of PCs, T3 can inhibit the posttranslational conversion of pro‐TRH to mature TRH.

#### 3.1.4 TH Inhibits TRH Release

In addition to regulating TRH transcription and maturation, THs also appear to inhibit TRH release by the PVN neurons (Figure 3A). PTU treatment or thyroidectomy‐induced hypothyroid conditions in rats resulted in significantly increased release of TRH into the hypophyseal portal blood, with a concomitant decrease in TRH content in the ME [132]. Conversely, T4 treatment decreased the TRH release into the portal blood [133]. It cannot be completely ruled out that the altered TRH amount detected in the portal blood is due to T3‐regulated extracellular degradation of TRH released in the ME as described below. However, further studies showed that in rats treated with PTU, while the TRH peptide content in the PVN was upregulated, the content in the ME was downregulated, suggesting increased release of TRH under hypothyroid conditions; conversely, when the animals were treated with T4, the TRH peptide in the PVN was downregulated, while its content in the ME was upregulated, suggesting reduced release of TRH [130]. Taken together, although the molecular mechanism has yet to be elucidated, lines of evidence support that THs may directly inhibit the release of TRH in the ME.

#### 3.1.5 Summary—Amplified Inhibition of TRH by T3 via Multistep Signaling

**FIGURE 4:**
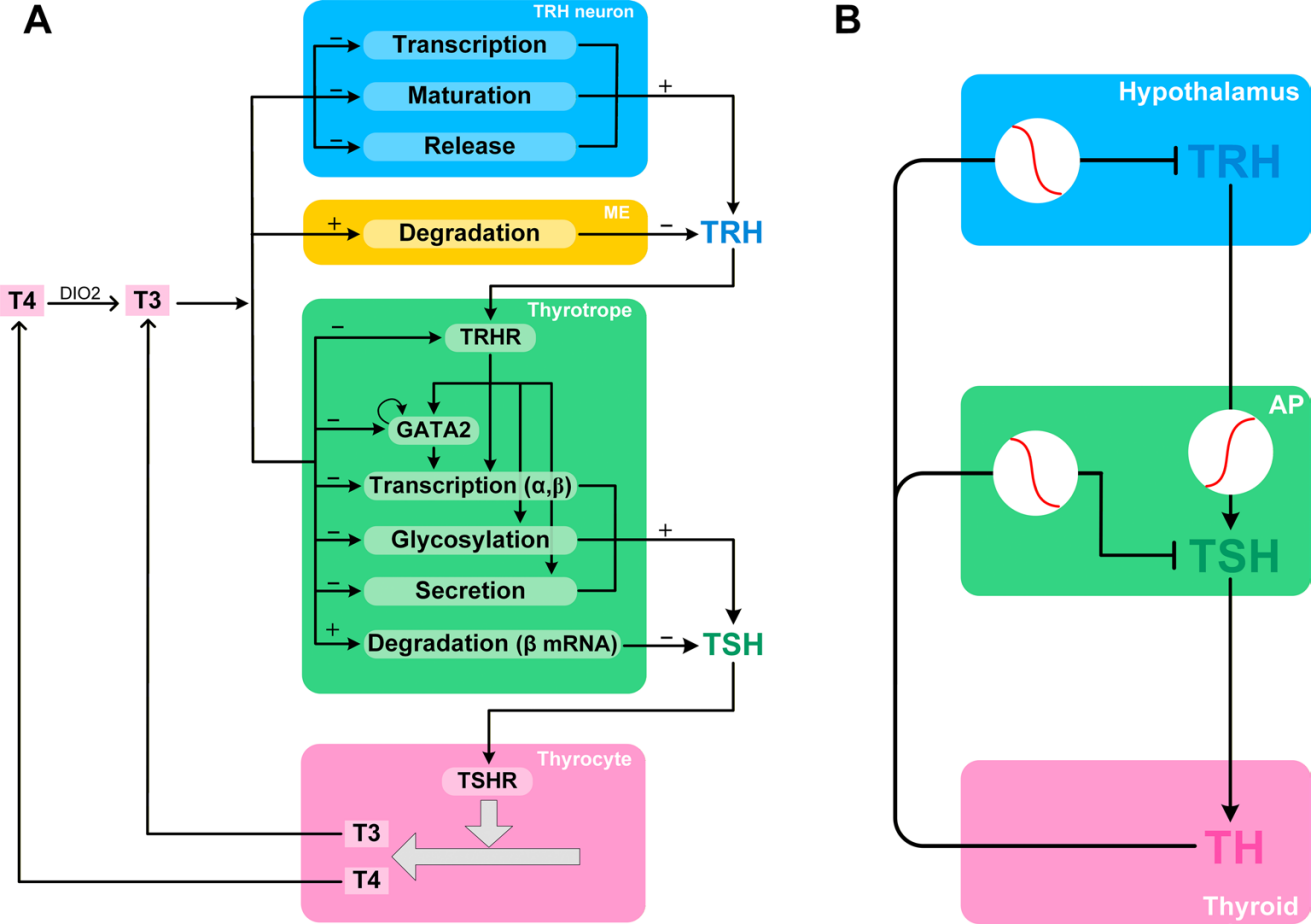
A summary of the multistep signaling pathways mediating the amplified feedback inhibition of TRH and TSH by T3. (A) In the TRH neuron, T3 inhibits the transcription, maturation, and release of TRH. In the ME, T3 promotes the degradation of TRH. In the thyrotrope, TRH stimulates TSH via TRHR by increasing the transcription of GATA2, TSHα, and TSHβ, and glycosylation and secretion of TSH. T3 inhibits the transcription of TRHR, GATA2, TSHα, and TSHβ, and glycosylation and secretion of TSH, and promotes the degradation of TSHβ mRNA. These multistep signaling pathways converge to underpin a highly ultrasensitive response of TRH and TSH to T3 inhibition. (B) Illustration of locations of signal amplification in the HPT feedback loop. The red rising sigmoidal curve in a white circle denotes amplified stimulation; the red falling sigmoidal curve in a white circle denotes amplified inhibition.

In summary, T3 can inhibit TRH in multiple concomitant ways, at the steps of (i) transcription, (ii) maturation, and (iii) release (Figure 4). Given that multistep signaling is a common mechanism of ultrasensitivity [33], when these parallel actions of T3 converge synergistically, ultrasensitivity can potentially arise, producing amplified inhibition of TRH by T3, with a theoretical logarithmic gain of 3. It was demonstrated in methimazole and perchlorate‐induced hypothyroid male mice that intraperitoneal (i.p.) injection of T3 or Sob‐AM2, a CNS‐penetrating thyromimetic, can produce a highly steep inhibitory dose–response in TRH mRNA levels [60]. This suggests that, among the three T3 signaling steps above, transcriptional regulation of TRH alone is already ultrasensitive, albeit the underlying mechanism remains to be determined.

### 3.2 T3 Stimulates TRH Degradation in ME

#### 3.2.1 Tanycytic PPII Catalyzes TRH Degradation in ME

After TRH is released from the axonal terminals into the interstitial space in the ME, it diffuses to the fenestrated capillaries of the hypophyseal portal vessels. Its bioavailability to the AP is regulated by β2‐tanycytes which control the postsecretory metabolism of TRH in the ME [134]. As a subtype of tanycytes, β2‐tanycytes line the floor of the third ventricle and project their long, basal processes to the external layer of the ME. Their end feet intermingle with the varicosities of the axonal terminals of the TRH neurons and terminate proximal to the portal capillaries [89, 135, 136]. β2‐tanycytes regulate the bioavailability of TRH to the portal vasculature through both physical and biochemical means. (i) They use their end feet as a physical barrier to block the access of TRH molecules to the capillaries, and (ii) they express a membrane‐bound ectoenzyme, known as pyroglutamyl peptidase II (PPII) or TRH‐degrading ectoenzyme (TRH‐DE), to degrade the tripeptide (Figure 3B) [134]. PPII protein is a zinc‐dependent metallopeptidase with a large extracellular domain which specifically catalyzes the hydrolysis of the pGlu‐His bond of TRH [136]. Therefore, PPII can regulate the TRH concentration in the interstitial space of the ME, controlling the amount of TRH reaching the hypophyseal portal vessels.

#### 3.2.2 TH Upregulates PPII Expression and Activity in Tanycytes

PPII mRNA is expressed in tanycytes, particularly in β2‐tanycytes [136], which can be regulated by THs (Figure 3B). It was demonstrated in rats that i.p. administration of T4 dramatically increased the PPII mRNA expression in β2‐tanycytes and its enzymatic activity, and inhibiting PPII activity increased the recovery of TRH from the ME [136]. Similarly, in hypothyroid mice, both T4 and T3 treatments induced PPII mRNA expression in β‐tanycytes in 5 h, before any TRH mRNA changes could be detected and the effect of T4 was DIO2‐dependent [137]. Conversely, hypothyroidism induced by methimazole markedly downregulated PPII mRNA in β2‐tanycytes in rats, which can be reversed with T4 treatment [138]. TRβ2 appears to be abundantly expressed in the third ventricle floor in rats [139], where tanycytes are located. Recently, it has been demonstrated, using RNAScope, that TRα2 mRNA is more abundantly expressed than TRβ in these tanycytes in rodents [140]. It remains to be determined how these TR isoforms mediate the transcriptional regulation of PPII by THs. Regardless, it is evident that T3 induces PPII expression on the cell surface of β2‐tanycytes to promote TRH degradation in the ME, which provides another route to mediate the feedback action of THs (Figure 4), in addition to the multistep ultrasensitive T3 inhibition of TRH in the PVN neurons described above.

### 3.3 Multistep TRH Stimulation and T3 Inhibition of TSH in Thyrotropes in AP

#### 3.3.1 Multistep Stimulatory Actions of TRH in Thyrotropes

In mammals, the biological actions of TRH are mediated by two membrane‐bound TRH receptors (TRHR), TRH‐R1 and 2, which are encoded by two separate genes and differentially expressed in various brain regions and peripheral tissues [141]. Only TRH‐R1 is expressed in the pituitary to mediate TRH signaling in the thyrotropes [142, 143, 144]. As a member of the G protein‐coupled receptor (GPCR) superfamily, TRH‐R1 mediates the stimulatory action of TRH by activating the transcription, glycosylation, and release of TSH subunits. The binding of TRH‐R1 by TRH activates Gα_q/11_ to trigger the phospholipase C (PLC) and protein kinase C (PKC) pathway (Figure 3C) [141, 145, 146]. PLC catalyzes the breakdown of (PIP_2_) in the cell membrane into inositol‐1,4,5‐trisphosphate (IP_3_) and diacylglycerol (DAG). It was observed, in mouse thyrotropic pituitary (TtT) cells, that IP_3_ can mobilize Ca^2+^ from the internal store, causing TSH release [147]. Ca^2+^ release from the internal store, along with activation of PKC by DAG, is also required for TRH‐stimulated TSHβ promoter activity observed in transfected GH_3_ cells [148, 149]. In comparison, in primary pituitary cells from CD‐1 rats, TRH‐stimulated TSHβ gene induction appears to require Ca^2+^ influx through the L‐type voltage‐gated channels and activation of PKC [150]. While canonically PKC is activated by DAG and Ca^2+^, it has been recently demonstrated that PKC is inhibited by casein kinase 1α (CK1α) in mouse pituitary cells, and TRH can inhibit CK1α to activate PKC thereby inducing TSHβ gene expression [151]. TRH‐stimulated TSH production may also be mediated via phosphorylated calmodulin‐dependent kinase IV (CaMKIV) and CaMKII in primary pituitary cells from CD‐1 rats, where CaMKIV can phosphorylate and activate CREB [152].

##### 3.3.1.1 TRH Stimulates TSHα and TSHβ Transcription

TSH is a 28–30 kDa glycoprotein hormone containing two apoproteins, a commonly shared α subunit and a TSH‐specific β subunit, which are products of two separate genes [153]. TSHα is encoded by the chorionic gonadotropin‐α (CGA) gene, common to several other glycoprotein hormones, including luteinizing hormone, follicle‐stimulating hormone, and chorionic gonadotropin [154]. The TSHβ subunit is unique, conferring specificity to TSH for receptor binding and biological responses [155]. TRH can stimulate the transcription of both subunit genes [156].

###### 3.3.1.1.1 TSHα

The human TSHα gene promoter contains a GATA‐responsive element (GATA‐RE), which can be transactivated by GATA2 [157]. TRH can stimulate TSHα gene promoter activity in a GATA2‐dependent manner [158]. Downstream of the GATA‐RE, the human TSHα gene promoter also contains two CREs and a binding site for P‐Lim (Lhx3a), a LIM homeodomain transcription factor (Figure 3C) [159]. CREB can bind to the CRE sites [160]. TRH stimulates phosphorylation of CREB, leading to recruitment of CREB binding protein (CBP) to both CREB and P‐Lim to synergistically enhance TSHα transcription [161]. The physical interactions of CBP with CREB and P‐Lim seem to involve different domains of the CBP protein. In a rat pituitary cell line α‐23, it was demonstrated that cAMP activated TSHα promoter activity, which involved recruiting phosphorylated CREB, CBP, and P300/CBP‐associated factor (PCAF) to the CRE sites, leading to increased H4K5 and H4K8 acetylation and decreased H4K20 trimethylation [162]. Although cAMP is elevated by TRH stimulation, the phosphorylation of CREB does not appear to involve PKA; rather, it may require CaMKIV [152]. The involvement of PKA was only suggested in TRH‐stimulated GH3 cells transfected with TRHR, where phosphorylation of serine 133 (S133) of CREB was detected [161]. However, since S133 can be phosphorylated by a variety of kinases [163], observing a concurrent increase in cAMP is no guarantee that PKA is the kinase responsible for the phosphorylation of S133 of CREB. In a mouse thyrotrope tumor cell line MGH101A, TSHα mRNA expression was stimulated by cAMP as well as by phorbol esters, suggesting that PKC may be involved [164]. The involvement of PKC in the gene transcription of TSHα was subsequently demonstrated, albeit in non‐thyrotrope lines, including LβT2 gonadotrophs [165], and rat pituitary GH4C1 cell line, where phosphorylation of the N‐terminal Lim1 domain of P‐Lim by PKC was suggested to mediate TRH‐stimulated recruitment of CBP to P‐Lim on the TSHα gene promoter [166]. Lastly, TSHα can also be activated by LIM/homeobox protein Lhx2 in thyrotropes [167].

###### 3.3.1.1.2 TSHβ

The transcriptional regulation of TSHβ requires several transcription factors (Figure 3C), including both the hematopoietic transcription factor GATA2 and pituitary‐specific transcription factor (Pit‐1) [168, 169]. The upstream sequence of the TSHβ gene contains a P1 region (−133 to −88) hosting several Pit‐1‐binding sites [170, 171]. It was demonstrated in monkey kidney CV1 cells that TRH‐stimulated TSHβ promoter activity required CBP, and the activity can be synergized by cotransfecting Pit‐1 which recruited CBP [161]. Phosphorylation of Pit‐1 by either PKC or PKA can enhance its binding to the human TSHβ promoter containing the Pit‐1‐binding element [172]. Proximally downstream to the P1 region also exist two GATA2‐binding sites [168]. While either Pit‐1 or GATA2 alone produced some basal promoter activity, cotransfection of both into CV‐1 cells synergistically enhanced mouse TSHβ promoter activity [168, 173, 174]. This synergy depended on the interactions of Pit‐1 and GATA2, sitting on the TSHβ promoter, with TR‐associated protein 220 (TRAP220)/MED1. TRAP220/MED1 is a component of the SRB/MED‐containing cofactor (SMCC) complex that regulates the function of RNA pol‐II and its deficiency led to pituitary hypothyroidism and growth retardation [174, 175, 176, 177]. In addition, a suppressor region immediately downstream of the GATA2 binding sites, which can be occupied and masked by Pit‐1, is also likely involved in the synergistic action between GATA2 and Pit‐1 [178]. Activation of the GATA2‐dependent TSHβ promoter activity by TRH is mediated by PKC, which promotes the DNA binding of GATA2 via the zinc finger region [158]. Induction of the TSHβ gene by TRH may also involve the activation of transcription factor NR4A1 through a PKC and ERK1/2‐dependent pathway [179]. In the thyrotrope‐derived TαT1 cell line, treatment with TRH or cAMP can induce the expression of LIM/homeobox protein Lhx2, and overexpressing Lhx2 stimulated the activity of a TSHβ reporter gene [180]. Lhx2 can bind to the −118 to −108 and −86 to −68 regions of the TSHβ promoter, both of which are required for Lhx2‐stimulated reporter gene activity. Because the Lhx2 binding sites overlap with the Pit‐1 and GATA2 binding regions, Lhx2 may compete with Pit‐1 and GATA2 for DNA binding and association with MED220 [180].

##### 3.3.1.2 Positive Autoregulation of GATA2

The GATA2 gene can be transactivated by its own translational product, the GATA2 protein, via multiple GATA‐REs in the gene promoter, which has been demonstrated in a number of cells and tissues, including trophoblasts [181], early hematopoietic cells [182], and midbrain [183]. Hence, GATA2 expression is controlled by an autoregulatory positive feedback loop (PFL). It was recently proposed that this autoregulatory PFL may also operate in thyrotropes (Figure 3C) [184]. Mouse thyrotrope TαT1 cells stimulated by TRH exhibited a tendency of increased GATA2 mRNA levels [180]. 12‐O‐tetradecanoylphorbol‐13‐acetate (TPA), a PKC activator, enhanced the expression of GATA2 protein in gonadotroph‐derived LβT2 cells, suggesting that GATA2 gene expression induced by TRH may be mediated by the PKC pathway to stimulate TSH transcription [158]. Since PFL is one of URMs, this positive autoregulation of GATA2 has the potential to steepen the TSH response to TRH given that both TSHα and TSHβ are target genes of GATA2 as described above, thus enhancing the feedback amplification.

##### 3.3.1.3 TRH Regulates TSH Glycosylation

TSH belongs to the family of glycoprotein hormones, with the glycan moiety representing 15%–25% of its molecular weight [185]. Human TSH (hTSH) contains three asparagine (Asn) N‐glycosylation sites [185, 186]. TSHα has two and TSHβ has one N‐linked carbohydrate chain. TSH glycosylation plays important roles in TSH assembly, secretion, biological activity, and clearance [187, 188]. Specifically, N‐glycosylation of TSHα modulates the signal transduction activity of TSH upon its engagement with TSHR, and de‐N‐glycosylation improves TSH activity. TSHβ glycosylation is important for the production of the TSH heterodimer [189]. De‐sialylation of recombinant hTSH increased its bioactivity in vitro, while the lack of sialic acids decreased TSH activity in animal models. Resialylation of the terminal Gal reversed these effects. Removal of terminal GlcNAc reduced the activity of recombinant hTSH in vivo [190].

The glycosylation pattern of TSH can be regulated by TRH [188]. In cultured rat pituitaries, TRH can stimulate the incorporation of glucosamine into TSH [191, 192]. In vivo treatment of newly thyroidectomized rats with TRH increased the content of specific high‐mannose species of oligosaccharides along with the addition of glucose residues, particularly in TSHβ, suggesting that TRH can regulate the kinetics of early carbohydrate processing [193]. It was shown in dispersed mouse thyrotrophic tumor cells that the initial glycosylation with high‐mannose oligosaccharides can promote α‐β subunit association and may protect against intracellular aggregation and degradation of TSH subunits [194]. Moreover, TRH can alter the carbohydrate structure of TSH to less complex forms, that is, more biantennary and/or truncated [188, 195]. Despite these early studies, the mechanism of TRH‐regulated glycosylation of TSH is largely unknown.

##### 3.3.1.4 Summary—Multistep Transcriptional and Posttranslational Pathways Underpinning Potential Ultrasensitive Stimulation of TSH by TRH

In summary, by activating TRHR, TRH can stimulate TSH via multiple pathways, including (i) activation of several transcription factors that coordinately induce the transcription of both TSHα and TSHβ subunits, (ii) autoregulation of GATA2 as a FPL to further enhance TSHα and TSHβ transcription, and (iii) glycosylation of TSH protein (Figure 4). The convergence of these steps can be synergistic and thus potentially ultrasensitive, producing an amplified TSH response to TRH stimulation. In keeping with this, it was demonstrated in rats that i.v. injection of TRH produced a sigmoidal dose–response of plasma TSH that spans only one order of magnitude of the TRH dose to stimulate TSH from 10% to 90% of the maximal TSH level, steeper than a typical Michaelis–Menten response that requires an 81‐fold dose change [196].

#### 3.3.2 Multistep Inhibitory Actions of T3 in Thyrotropes

It has long been observed that elevating the circulating TH levels can rapidly inhibit the pituitary responsiveness to TRH and reduce the serum TSH concentrations [197, 198]. While the decrease in serum TSH is in part owing to the T3‐mediated feedback inhibition of TRH synthesis and release as described above, T3 also exerts a direct inhibitory action on TSH in the AP. In TRH knockout mice, methimazole‐induced reduction of T4 caused increases in serum TSH levels, suggesting a direct effect of THs on TSH secretion [199]. In thyroidectomized hypothyroid rats injected with T3, the pituitary nuclear T3 levels were found to be inversely correlated with the circulating TSH levels [18]. The direct pituitary action is also evidenced in cultured rat or mouse pituitary cells where T3 inhibited the TSH release in a concentration‐dependent manner and the release was completely blocked at high T3 concentrations [200, 201, 202]. T3 in the AP appears to come from two sources. (i) As aforementioned, T3 can originate from T4 deiodination in the tanycytes in the MBH, from which it is transported into the pituitary through the hypophyseal portal vessels. (ii) T3 is also produced locally in the AP where DIO2 is highly expressed [87, 88], including thyrotropes [203]. In pituitary cells‐specific DIO2 KO mice, the circulating TSH and T4 levels increased with normal T3 and downregulated TRH mRNA levels [19].

##### 3.3.2.1 T4 Regulates DIO2 Activity in Thyrotropes

Compared with the DIO2 in the tanycytes in the hypothalamus, which is poorly ubiquitinated and largely unregulated by T4 [96], the DIO2 in the AP seems to be regulated by the circulating T4 level. In rats treated with EMD 21388, which inhibits T4‐TTR binding causing a transient rise in serum fT4, DIO2 activity was downregulated in the pituitary without significant T3 changes despite decreased serum TSH levels [204]. DIO2 mRNA in the AP was upregulated by hypothyroid and downregulated by hyperthyroid conditions in rats [88, 205]. Hypothyroidism increased DIO2 mRNA expression in TSH‐containing thyrotropes in rats [203]. DIO2 activity, measured as conversion of T4 to T3, seemed to be upregulated in the pituitary and other brain regions in adult rats under hypothyroid conditions induced by PTU, methimazole, thyroidectomy, and iodine deficiency, while the local T3 concentrations decreased, albeit not statistically significant [206]. In murine‐derived thyrotrope cells, TαT1, despite that T4 induced ubiquitination‐mediated DIO2 degradation and downregulation of its enzymatic activity, the conversion of T4 to T3 still increased as T4 concentration increased, with simultaneously observed repression of TSHβ mRNA expression [203]. In this study, the responsive range seemed to be within the euthyroid and hypothyroid T4 concentrations, while at higher T4 concentrations, T3 production did not increase much further and the decline of TSHβ also reached a plateau. The authors attributed the T3 production increase to the argument that DIO2 is synthesized at a high rate surpassing the maximal ubiquitination rate that can be induced by T4. More recently, Batistuzzo et al. showed, with mouse pituitary explants, that although T4 reduced DIO2 activity, the reduction was insufficient to offset the effect of increased T4 concentration, such that as fT4 doubled from 10 to 20 pM, T3 production still increased by 1.4 fold [207]. Taken together, it appears that although T4 can downregulate DIO2 activity in thyrotropes, the local T3 concentration can still respond to the circulating T4 level, albeit with a reduced sensitivity.

##### 3.3.2.2 T3 Inhibits TSHα and TSHβ Transcription via TRβ and nTRE

###### 3.3.2.2.1 TSHα

T3 can directly inhibit TSHα transcription via TRβ, which interacts with an nTRE near the transcription start sites on both human and rat TSHα genes (Figure 3C) [208, 209]. Using transient transfection, Langlois et al. found that the human TRβ2 isoform plays a unique role by exhibiting ligand‐independent (constitutive) activation and T3‐dependent repression of the TSHα gene, and this function requires the N‐terminal amino acids 89–116 of TRβ2 [111]. The negative transcriptional regulation of the TSHα gene by T3 involves a mechanism counterintuitive to the classical role of corepressors. In the absence of T3, a corepressor complex, containing NCoR/SMRT, transducin β‐like protein 1 (TBL1), and histone deacetylase 3 (HDAC3), is associated with the TR‐RXR heterodimer bound to the TSHα gene promoter. When T3 is present, components of this corepressor complex dissociate from the promoter, resulting in increased H3K9 and H3K18 acetylation, decreased H3K27 acetylation, and increased H3K4 and H3K27 trimethylation, which lead to suppression of TSHα gene transcription [162, 210].

###### 3.3.2.2.2 TSHβ

In the rat, a single TSHβ gene is transcribed into two mRNA isoforms, the long mRNA1 and short mRNA2, by using two alternative promoters. Only mRNA2 was significantly regulated by THs [211]. In cultured TtT 97 cells which originated from a thyrotropin‐producing mouse pituitary tumor, T3 can inhibit the TSHβ promoter activity in a concentration‐dependent manner [212]. In radio‐thyroidectomized mice, exogenous T3 decreased pituitary TSHβ mRNA levels to a greater extent than TSHα mRNA levels [213]. Deletion analysis suggested that T3‐mediated inhibition of TSHβ transcription may involve DNA segments near its transcription start site (Figure 3C). For the rat TSHβ gene, a 57‐bp DNA fragment containing 17 bases of 5′‐flanking sequence, exon 1, and 13 bases of the first intron may host the cis‐regulating elements that mediate the inhibition by T3 [214]. Later studies further narrowed the negative regulatory element down to the 17‐bp motif between +11 and + 27 at the 3′ end of exon 1, which can be bound by TR [215]. Subsequently, a nonpalindromic half‐site motif of consensus TRE was identified as the nTRE in the region [216]. In TtT 97 cells, the T3‐regulatory site appears to be within the 46 bp of the 5′‐flanking region and 3 bp of the first exon [212]. For the human TSHβ gene, the transcriptional repression by T3 appears to involve the +9 and + 37 bp of the first exon which can be bound by TRβ [217]. It was further demonstrated with detailed functional and structural scan mutation analysis that the first exon of human TSHβ contains two TRβ‐binding sites at +3 to +13 bp and + 28 to +37 bp, each containing one or two of the consensus half‐site nTRE [218]. The upstream one can be bound by a TRβ dimer with higher affinity, while the downstream one by a TRβ monomer with lower affinity. To distinguish the roles of TR isoforms, Chiamolera et al. found that T3‐mediated downregulation of TSHβ in TαT1.1 cells, a unique mouse pituitary thyrotrope cell line, can be abolished by either simultaneous knockdown of both TRβ and TRα for T3 concentrations up to 100 nM or single knockdown of TRβ for T3 concentrations at 10 not 100 nM using shRNA‐expressing adenoviruses, while the knockdown of TRα alone had no effect [219]. The study suggested that TRα may mediate the inhibition of TSHβ when T3 is at high concentrations. However, TRα has also been suggested to play a positive role in regulating TSHβ, as shown in TRα knockout mice which are hypothyroid with significantly lower pituitary TSHβ mRNA levels and die prematurely in 5 weeks after weaning, suggesting that TRα may turn on “adult‐type” production of THs during weaning [220]. Beside through nuclear TRs, it has also been shown that L‐T4 can activate the integrin αVβ3 membrane receptor with a rapid nongenomic effect to inhibit PKC/ERK/CREB signaling, thus repressing TSHβ transcription [151].

##### 3.3.2.3 T3 Inhibits TSHα and TSHβ Transcription via TRβ2 and GATA2

Besides directly binding to nTREs via TRβ2 to inhibit TSHα and TSHβ transcription, T3 can inhibit the two subunit genes indirectly by (i) interfering with the transcriptional activity of GATA2 and (ii) repressing the transcription of the GATA2 gene itself (Figure 3C). T3 inhibited the TSHβ gene promoter activity driven by Pit1 and GATA2 coexpressed in CV1 cells, and the inhibition is mediated most strongly by TRβ2, not other TR isoforms [173]. TRβ2 is highly expressed in TαT1 thyrotrope cells compared with other TR isoforms [173]. When T3 binds to TRβ2, its ligand‐binding domain interacts with the LXXLL motif in TRAP220, destabilizing the interaction between the TRAP220 and the Pit1‐GATA2 complex [175]. Histone deacetylase 3 (HDAC3) is also recruited or histone acetyltransferase (HAT)‐related molecules are dissociated, resulting in deacetylation of histone H4, which in turn inhibits TSHβ transcription. In this scenario, the nTREs may be dispensable for the repression of TSHβ by T3 [175]. In a ligand‐independent manner, TRβ2 can interact via its DNA‐binding domain (DBD) with the zinc finger of GATA2 bound to the TSHβ promoter [175, 221]. It was further shown that T3 may promote the dissociation of TR from the nTRE and enhance GATA2 binding to GATA2‐RE by binding to GATA2‐bound TR, resulting in transcriptional repression of target genes [221].

More recently, it was demonstrated that the repression of TSHα and TSHβ by T3 also involves transcriptional downregulation of GATA2 itself. The positive autoregulation of GATA2 can be inhibited by T3‐liganded TRβ2 via the interaction between its DBD domain and the zinc finger of GATA2 [184]. This was demonstrated by (i) the repression of the reporter gene activity by T3 in CV1 cells expressing TRβ2 and harboring plasmids containing multiple GATA2‐REs from the GATA2 gene promoter, and (ii) the observation that T3 inhibited GATA2 mRNA and protein expression in LβT2 gonadotroph cells. Since GATA2 positively regulates TSHα and TSHβ transcription, the inhibition of GATA2 by T3 is a key mechanism underpinning the suppression of both genes by T3 [184].

##### 3.3.2.4 T3 Induces TSHβ mRNA Destabilization

In addition to transcriptional repression, T3 can also regulate TSHβ mRNA posttranscriptionally through altering its stability (Figure 3C). Both in rat pituitary culture and in vivo, T3 treatment reduced the half‐life of TSHβ mRNA, which may have resulted from the shortening of its poly(A) tail, while TSHα mRNA was not affected [222]. A similar observation was also made in murine thyrotrophic TtT97 tumor cells [223]. In both species, the half‐life was reduced from about 24 h to 6–9 h and the length of the poly(A) tail was shortened from 160 to 180 to about 30 nt. Hypothyroidism induced by thyroidectomy in rats can increase the poly(A) tail length of the TSHβ mRNA, while T3 treatment of these hypothyroid rats led to shortening of the tail, and these changes can occur as fast as in 30 min [224]. A cytoplasmic protein of approximately 80–85 kDa was identified in rat pituitaries, which binds to the TSHβ mRNA 3′‐UTR region containing a consensus sequence and could be responsible for T3‐induced fast mRNA turnover [225]. T3‐regulated binding of poly(A) binding protein (PABP) and TSHβ BP2 to the 3′‐UTR of TSHβ mRNA were suggested to play a role in this regard [226, 227]. In the study by [224], the recruitment of the TSHβ mRNA to ribosomes in hypothyroid rats was also inhibited by T3, suggesting that TSHβ can be regulated at the translational level as well.

##### 3.3.2.5 T3 Inhibits TRHR Transcription

T3 can decrease the specific binding of TRH in rat pituitary cells in a concentration‐dependent manner, but it was unclear whether this is due to a decrease in the TRHR level or of its binding affinity [228]. Using mouse pituitary thyrotropic tumor cells, Gershengorn reported that T3 downregulated TSH release and TRHR abundance in a concentration‐dependent manner, while the affinity for TRH remained unchanged [202]. Yamada et al. reported elevated TRHR mRNA levels and TRHR binding in the AP along with rising circulating TSH in thyroidectomy‐induced hypothyroid rats [229]. However, in GH3 cells, T3 treatment did not induce changes in TRHR mRNA levels [229]. In SD rats, the TRHR mRNA in the AP was reduced to 35% of the control level 4 h after i.p. injection with T3, and was increased by 2 fold 4 days after PTU treatment, suggesting that T3 inhibits TRHR transcription [230]. Chiamolera et al. found in TαT1.1 mouse pituitary thyrotrope cells that TRβ knockdown can block T3‐induced downregulation of TRHR mRNA, suggesting that T3 may act through TRβ to suppress TRHR expression (Figure 3C) [219].

##### 3.3.2.6 TH Regulates TSH Glycosylation

Several studies demonstrated that the degree of sialylation of TSH is elevated in hypothyroid patients and hypothyroid rats [231, 232]. Wide and Eriksson reported that in patients of severe primary hypothyroidism, circulating TSH is more sialylated as well as less sulfonated and N‐glycosylated, and L‐T4 treatment can reverse this glycan pattern [233]. Secretion of highly sialylated TSH may result from augmented α‐2,6‐sialyltransferase activity in thyrotropes [234]. It is unclear how much of the glycosylation change is due to a direct effect of T3 (and by which mechanism) in the thyrotrope or an indirect effect via TRH. Enhanced sialylation prolongs the half‐life of TSH yet reduces its intrinsic bioactivity. The reason for the increased stability of highly sialylated TSH is that it can evade hepatic clearance mediated by the SO3‐N‐acetylgalactosamine‐receptor [235]. Simultaneously stabilizing TSH and reducing its biological activity is counterintuitive as they have opposite effects on the overall activity of TSH; thus, the significance of such opposing dual‐regulation of TSH is unclear.

##### 3.3.2.7 T3 Upregulates PPII Expression in AP

While PPII expressed on the surface of the tanycytes in the ME can cleave TRH to reduce its availability to the AP, PPII is also expressed on the adenohypophyseal cells to degrade the TRH molecules reaching the pituitary, at least in rats [236]. PPII activity can be robustly upregulated by T3 in rat adenohypophysis, and hypothyroidism resulted in an opposite effect [236, 237]. PPII mRNA levels in the AP were markedly increased in rats after a single injection of T3, whereas PTU treatment reduced the levels of PPII transcripts [230, 238]. In SD rats, after a single injection of T4, the PPII activity in the AP increased [136]. In situ hybridization combined with immunocytochemistry in rats showed that two thirds of PPII mRNA‐expressing cells in the AP are lactotrophs, and one third are somatotrophs while thyrotropes do not appear to express PPII mRNA [236, 239]. PPII expressed in the lactotrophs can be upregulated by T3 and downregulated by TRH [240]. This suggests that PPII is involved in TH regulation of prolactin secretion stimulated by TRH. It is unclear how much the induced PPII on the surface of these two pituitary cells by THs can affect the local concentrations of TRH at the surface of the neighboring thyrotropes.

##### 3.3.2.8 Summary—Amplified Inhibition of TSH by T3 via Multistep Signaling

In summary, T3 can act in the AP to inhibit TSH via multiple pathways, including (i) directly or indirectly through GATA2 repressing the transcription of both TSHα and TSHβ subunits, (ii) destabilizing TSHβ mRNA, (iii) altering glycosylation of TSH protein, (iv) repressing TRHR to inhibit TRH‐stimulated downstream signal transduction (Figure 4). The convergence of these steps can be synergistic and thus potentially ultrasensitive, producing amplified inhibition of TSH by T3.

### 3.4 Experimental Evidence of Ultrasensitive Responses in Signaling Pathways Mediating T3 Inhibition of TRH and TSH

In the absence of T3, the TR homodimer can bind to different arrays of TREs with some degree of cooperativity [241]. The DBD‐LBD construct of TRβ1 can bind to the nTREs on the TSHβ gene with a Hill coefficient of 3.5 [221]. In the presence of T3, the Hill coefficient is reduced to 1.7 with the binding affinity also weakened. When RXR is also present, the Hill coefficient is 1.8 with slightly improved affinity. The GATA2 zinc finger or GATA2 zinc finger plus the TRβ1 DBD‐LBD construct can bind to the GATA‐RE of the TSHβ gene with Hill coefficient > 3 and a high Kd; however in the presence of T3, the binding affinity is enhanced by nearly 20 fold or greater while the Hill coefficient is reduced to 1 [221]. T3 inhibits the expression of GATA2 mRNA in LβT2 gonadotroph cells in a sigmoidal manner without the Hill coefficient value reported [184]. The authors argued that the positive autoregulatory loop of GATA2 has a nonlinear role in mediating the log‐linear inverse relationship between TSH and fT4 observed clinically. In hypothyroid male mice, i.p. injection of T3 or Sob‐AM2, a CNS‐penetrating prodrug of thyromimetic sobetirome, can produce nearly switch‐like dose response of hypothalamic TRH mRNA inhibition in 6 h [60]. In the same study, T3 and sobetirome can also induce steeply sigmoidal inhibition of TSHβ mRNA in the pituitary. Taken together, it appears that multiple intermediate and terminal events in the signaling pathways of feedback inhibition of TRH and TSH exhibit sigmoidal responses, which amplify the feedback signal mediated by T3.

The feedback amplification gain may be compromised in pathological conditions. In families with autosomal dominant mutations of the TRβ gene, amino acid substitutions owing to point mutations, such as E460K or A317T, result in reduced binding affinity of T3 for TRβ2. As a result, the sensitivity of TSH to T4 inhibition is reduced, manifested as a shift of the logTSH‐fT4 curve to high fT4 levels on the right [242]. This shift, however, also appears to be accompanied by a shallowing of the curve, suggesting a reduction in the overall feedback gain. In theory, individuals with lower feedback gains are likely to be more susceptible to thyroid perturbations.

### 3.5 Summary

The T3‐mediated negative feedback control is achieved at both the hypothalamus and AP. At the molecular level, the amplified inhibition of TSH by T3 is underpinned by multiple pathways, directly in the thyrotropes and indirectly via the TRH neurons and β2‐tanycytes. These pathways include transcriptional and posttranslational regulations of the synthesis, maturation, degradation, and release of TRH and TSH (Figure 4). Conceptually, the feedback amplifier in the brain is composed of three interconnected subamplifiers—amplifying respectively (i) the TH signal to inhibit TSH, (ii) the TH signal to inhibit TRH, and (iii) the TRH signal to stimulate TSH (Figure 4B). The three subamplifiers operate collectively to sustain a high feedback loop gain of the HPT axis.

## 4 Functional Significance of Central Location of Signal Amplification

**FIGURE 5:**
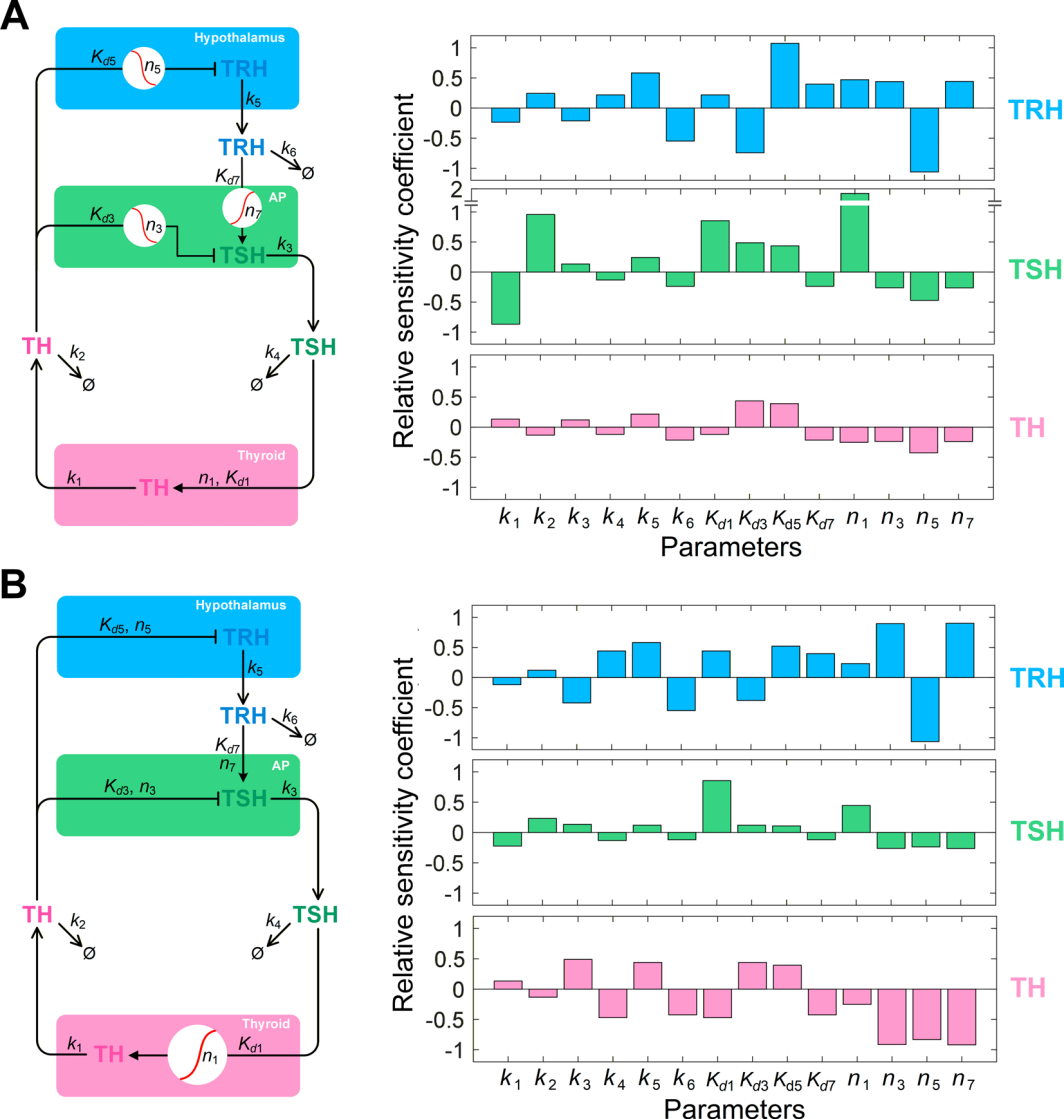
Mathematical models comparing two different amplification designs in the feedback loop of the HPT axis. (A) *Design A*: The amplifier is in the brain collectively contributed by one and two subamplifiers in the hypothalamus and AP respectively (left panel); parameter sensitivity analysis for steady‐state *TRH*, *TSH*, and *TH* levels as indicated (right panel). (B) *Design B*: The amplifier is primarily in the thyroid (left panel); parameter sensitivity analysis for steady‐state *TRH*, *TSH*, and *TH* levels as indicated (right panel). The red rising sigmoidal curve in a white circle denotes amplified stimulation; the red falling sigmoidal curve in a white circle denotes amplified inhibition. Local sensitivity analysis was conducted, where one parameter is varied at a time by 10% above and below its default value and the model was run until the new steady state was achieved. For both models, *k*_1_ = 0.722, *k*_2_ = 0.0048, *k*_3_ = 69.3, *k*_4_ = 0.693, *k*_5_ = 69.3, and *k*_6_ = 6.93. For *Design A*, *K*_*d*1_ = 9, *K*_*d*3_ = 8.66, *K*_*d*5_ = 5, *K*_*d*7_ = 3, *n*_1_ = 1, *n*_3_ = 4, *n*_5_ = 2, and *n*_7_ = 2. For *Design B*, *K*_*d*1_ = 1.7321, *K*_*d*3_ = 1.6667, *K*_*d*5_ = 1.6667, *K*_*d*7_ = 9, *n*_1_ = 4, *n*_3_ = 1, *n*_5_ = 1, and *n*_7_ = 1. Time unit: Hour.

An intriguing question is why nature's design of the HPT axis places the signal amplifier in the brain (herein referred to as *Design A*), as opposed to an alternative design that places the amplifier in the thyroid (*Design B*). We argue that *Design A* can more effectively resist perturbations at multiple sites of the HPT axis to keep THs close to the physiological operating levels and thus is evolutionarily more advantageous to maintain TH homeostasis than *Design B*. Below, we used simple mathematical models of the HPT feedback loop to compare the performances of the two alternative designs (Figure 5). The model for each design has three variables, *TRH*, *TSH*, and *TH* (representing T4 primarily), which are described by a common set of ordinary differential equations (ODEs):

(1) $$\frac{\text{dTRH}}{\mathrm{dt}}=k_{5}\cdot \frac{{K_{d5}}^{n_{5}}}{{K_{d5}}^{n_{5}}+{\mathrm{TH}}^{n_{5}}}-k_{6}\cdot \mathrm{TRH}$$

(2) $$\frac{\text{dTSH}}{\mathrm{dt}}=k_{3}\cdot \frac{{K_{d3}}^{n_{3}}}{{K_{d3}}^{n_{3}}+{\mathrm{TH}}^{n_{3}}}\cdot \frac{{\mathrm{TRH}}^{n_{7}}}{{K_{d7}}^{n_{7}}+{\mathrm{TRH}}^{n_{7}}}-k_{4}\cdot \mathrm{TSH}$$

(3) $$\frac{\mathrm{dTH}}{\mathrm{dt}}=k_{1}\cdot \frac{{\mathrm{TSH}}^{n_{1}}}{{K_{d1}}^{n_{1}}+{\mathrm{TSH}}^{n_{1}}}-k_{2}\cdot \mathrm{TH}$$

*k*_1_, *k*_3_, and *k*_5_ represent the maximal production rates of *TH*, *TSH*, and *TRH*, respectively; *k*_2_, *k*_4_, and *k*_6_ represent the first‐order rate constants for the clearance of *TH*, *TSH*, and *TRH* in the circulation, respectively. There are 4 Hill functions, where *K*_*d*1_, *K*_*d*3_, *K*_*d*5_, and *K*_*d*7_ represent the affinity constants for the stimulation of *TH* production by *TSH*, inhibition of *TSH* by *TH*, inhibition of *TRH* by *TH*, and stimulation of *TSH* by *TRH*, respectively; *n*_1_, *n*_3_, *n*_5_, and *n*_7_ are the corresponding Hill coefficients.

The major difference between the two designs lies primarily in the values of the Hill coefficients. When a Hill coefficient *n* is much greater than unity, then the corresponding Hill function provides some degree of percentage signal amplification where *n* represents the maximal logarithmic gain; conversely, when *n* is equal to or less than unity, then there is no signal amplification. *Design A* is an implementation of Figure 4B, which contains three subamplifiers (Figure 5A, left panel), one in the hypothalamus and two in the AP (indicated by the red sigmoidal curve in a white circle with *n*_3_, *n*_5_, and *n*_7_), which collectively provide amplified feedback inhibition of *TSH* by *TH*. The values of *n*_3_, *n*_5_, *n*_7_ are set to 2, 2, and 4, which produce an overall maximal feedback gain of 8 (2*2 + 4 = 8) according to signal transfer theory when the three Hill functions are far from being saturated [243]. The *TH* production by *TSH* in the thyroid is not amplified as *n*_1_ is set to 1, reducing the Hill function to Michaelis kinetics. Therefore, the overall feedback loop gain of the HPT axis in *Design A* is about 8.

*Design B* (Figure 5B, left panel) is different from *Design A* in that (i) the amplification is primarily in the thyroid, where the stimulation of *TH* production by *TSH* is amplified with *n*_1_ set to a value much greater than 1; (ii) *n*_3_, *n*_5_, and *n*_7_ are all set to 1 to reduce the Hill functions to Michaelis kinetics so that there is no amplification in each of the three individual signaling steps. However, it should be noted that since *TSH* is inhibited by *TH* both directly and indirectly through the *TRH* pathway, this dual‐step signaling also provides a small degree of amplification with a gain of about 2. By setting *n*_1_ = 4, the overall loop gain of the HPT axis in *Design B* is about 8, comparable to *Design A*.

In both models, *TH*, *TSH*, and *TRH* are cleared at vastly different rates, with *k*_2_, *k*_4_, and *k*_6_ set to values corresponding to half‐lives of 6 min, 1 h, and 6 days, respectively, according to the literature for these three human hormones [244, 245, 246]. This large time scale separation ensures that for a feedback loop like the HPT axis with a high loop gain, the system is still stable, not falling into oscillation [247]. *K*_*d*1_, *K*_*d*3_, *K*_*d*5_, and *K*_*d*7_ are set for each model such that at the basal steady‐state levels of the three hormones, each Hill or Michaelis step is at 10% saturation. *k*_1_, *k*_3_, and *k*_5_ are finally set so that the basal steady‐state levels of *TH*, *TSH*, and *TRH* are at 15, 1, and 1 respectively (arbitrary unit). The model code in MATLAB and Python is available at https://github.com/pulsatility/2025‐signal‐amplification‐in‐HPT‐axis.

For the two designs, we conducted a sensitivity analysis to compare how sensitive the steady‐state levels of the three hormone variables, especially *TH*, are to perturbations of the model parameters. The sensitivity analysis showed that for *Design A*, *TH* is highly insensitive to nearly all 14 parameters except *K*_*d*3_, *K*_*d*5_, and *n*_5_, which is in the brain (Figure 5A, right panel). The relative sensitivity coefficients of many parameters, especially those in the peripheral such as *k*_1_ and *K*_*d*1_, and those in the circulation such as *k*_2_, *k*_4_, and *k*_6_, are close to 0.1, indicating that as the parameter is perturbed by a certain percentage, for example, 10%, the steady‐state TH level only changes by a much smaller percentage, that is, 1%. In comparison, *TRH* and *TSH* are sensitive to a number of parameters. In contrast, for *Design B*, *TH* is quite sensitive to nearly all parameters except *k*_1_, *k*_2_, and *n*_1_, and so is *TRH*, while *TSH* is insensitive to many parameters (Figure 5B, right panel). Based on the result of the sensitivity analysis, it can be evidently argued that for the objective of keeping *TH* within a narrow physiological range, *Design A* is superior to *Design B* since the former is more robust against perturbations initiated at multiple sites in the HPT axis. As far as allostatic regulation is concerned, where *TRH* needs to be centrally regulated by neural signaling from other parts of the hypothalamus, for *Design A*, one way to achieve this goal is through regulating *K*_*d*5_, that is, the sensitivity of *TRH* to *TH* inhibition, or *n*_5_ in the TRH neurons, as these two are moderately sensitive parameters for altering *TH* (Figure 5A, right panel).

Admittedly, the models we used here are simplified, representing a barebone of the HPT axis without the complexities often found in full‐fledged models in many original research papers. Examples of limitations are not treating T4 and T3 separately and lack of their conversions and differential regulations. However, given the illustrative purpose of the current models, more complex models are beyond the scope of this review as the current models are sufficient to illustrate the performance difference between central vs. peripheral locations of the signal amplifier.

## 5 Discussion

### 5.1 Setpoint and Variability of the HPT Axis

In an electronic circuit, a setpoint, for example, the room temperature controlled by a thermostat, can be set by tuning the parameter value of a particular component, such as a resistor, on the circuit board. It is not entirely clear how similar control of a biological variable can be achieved in a biological system. The concept of setpoint, when invoked in the realm of neuroendocrinology, tends to be construed as an intrinsic property belonging to the hypothalamus, which acts as the sole determinant that dictates the steady‐state levels of the terminal effector hormones such as THs. Based on the inverse relationship between the circulating TSH and fT4, Fitzgerald and colleagues have argued against a centrally dictated setpoint model of the HPT axis [55, 248], and applied similar logic to other endocrine systems such as calcium and glucose regulations [249]. They argued that the operating fT4 level is collectively determined by both arms of the NFL of the HPT axis.

Therefore, variations in all participating organs/tissues of the HPT axis may affect the operating TH levels, albeit with different sensitivities, which depend on where the signal amplifier is located within the NFL. Genetic, epigenetic, age, sex, circadian rhythm, dietary iodine, and environmental factors may all contribute to the interindividual and intraindividual variations [39, 41, 43, 52, 53, 54]. Genetic and epigenetic factors play important roles in shaping the TH setpoints during fetal development and maturation of the HPT axis [52]. Using large cohorts of monozygotic and dizygotic twins and general populations, many studies have shown that heredity can contribute up to 70% of the variabilities in either free THs, TSH, or T4xTSH product [54, 250, 251, 252]. Large‐scale and genome‐wide association studies have identified a number of single‐nucleotide polymorphisms (SNPs) in candidate genes, which may be responsible for the variations in TSH and fT4 setpoint [53, 253, 254, 255]. These genes include those already known to participate in thyroid function, such as *DIO1*, *THRB*, *NF1A*, *TTF2*, and *TPO*, but also many others, including *PDE8B*, *PDE10A*, and *ITPK1* in the TSHR signal transduction pathway, *CAPZB* in the formation of microvilli and filopodia on the thyrocyte surface in the follicular lumen and TG endocytosis, *VEGFA* in angiogenesis, as well as *INSR* and *IGFBP5* in the GH/IGF‐1 signaling pathway. Interestingly, most of these polymorphic genes are expressed in the thyroid, which is consistent with the argument that HPT axis variability originates primarily in the thyroid, as inferred from the observed inverse TSH‐T4 relationship. In a genetic study using two different rat strains, salt‐sensitive Dahl (SS) and Brown Norwegian (BN), it was shown that while the two strains have comparable fT4 levels, the TSH level in BN rats is 3 times higher than in SS rats [256]. The study further demonstrated that the vastly different TSH levels are attributable neither to differences in the biological activity of the TSH molecules nor to differences in the intrinsic responsiveness of the TSHR molecules to TSH. Rather, the variance stems from the fact that TSHR expression in BN rats is only half that of SS rats. Epigenetic modifications including DNA methylation can also contribute to the observed variations in TSH and fT3 [257].

### 5.2 Allostasis—On‐Demand Setpoint Control

The setpoint of the HPT axis may be varied as a result of allostatic regulation in response to the body metabolic demand and physiological state, such as nutritional and energy status, exercise, ambient temperature, circadian rhythm, pregnancy, stress, and other nonthyroidal illness [26, 258, 259]. The regulation can be implemented centrally by altering TRH neuronal activities through integrating various neural and endocrine signals, including glucocorticoids, norepinephrine, leptin, neuropeptide Y (NPY), Agouti‐related protein (AgRP), and α‐melanocyte‐stimulating hormone [25, 63, 260, 261, 262]. When the allostatic command is exerted centrally via the TRH neurons, the TH setpoint can be regulated with ease. This is because the regulatory point is upstream of the amplifier; thus, in theory, it may provide a nearly linear control of TSH and T4, as predicted by feedback control theory [30].

### 5.3 Nature's Design for Biological Robustness

According to control theory, for homeostatic control of a given variable against direct perturbations, the amplification can be located or distributed in any part of the feedback loop as long as the aggregated loop gain reaches the same value. In the case of the HPT feedback system, alternative to the nature's design, that is, having the signal amplifier located in the hypothalamus and AP (Figure 5A), it is possible to have the amplifier located in the thyroid gland such that a small percentage increase in TSH can stimulate a larger percentage increase in T4 and T3 secretion (Figure 5B). According to loop gain theory [263, 264], in both designs, a preamp perturbation initiated in the brain (e.g., by changing *K*_*d*3_) can lead to a nearly linear response in THs, thus THs are potentially sensitive to perturbations initiated in the brain. In comparison, a perturbation initiated in the thyroid (e.g., by changing *K*_*d*1_) results in a much‐muted response of THs in *Design A*, but a sensitive disruption of THs in *Design B*. Therefore, *Design A*, that is, nature's design, is strongly favored for maintaining TH homeostasis because its sensitivity to perturbations is largely constrained to the brain (Figure 5A vs. Figure 5B).

Following system design principles on the tradeoff between robustness and fragility, as worked out by engineers for man‐made devices but equally applicable to natural systems, a system becomes overall more robust when it is configured in a way such that its sensitivity to perturbations is distributed as follows: the controlled variable (such as THs here) is insensitive to unstable components or those that are frequently perturbed by the environment, but only sensitive to components that are inherently stable or infrequently perturbed [265]. The HPT axis seems to adopt this principle. As argued above, with nature's design, THs are sensitive to perturbations initiated in the hypothalamus, where the TRH neurons and other input neurons reside. However, since the hypothalamus is within the blood–brain‐barrier (BBB), it is protected from being accessed by environmental endocrine disruptors. The BBB essentially hides the HTP axis' “fragility” from perturbation by external factors. In comparison, because of their functional needs to pass through peptide hormones, the ME, where the TRH neuron axons terminate, and AP are circumventricular organs (CVO) which are outside of the BBB [266, 267]. It is thus unfortunate that the ME and AP are subject to perturbations by endocrine disruptors in the circulation; however, the disruption is predicted to be moderate. As the most richly perfused organ, the thyroid gland is exposed to all environmental chemicals in the circulation; however, by nature's design, THs are more resilient to these perturbations. Therefore, by encapsulating part of the amplifier as much as possible within the BBB and having the thyroid as a less sensitive target of perturbations, nature's design confers increased overall robustness to the HPT axis.

Theoretically, depending on the manner in which the feedback signal is processed, there could be 3 different types of feedback control, that is, proportional, integral, and derivative (PID) [268]. A PID control system responds to the present, past, and projected future state of the controlled variable, respectively, and a combination of them is often found in man‐made devices to increase performance and robustness. Both proportional and integral feedback have been discovered in biological organisms [29, 30, 32, 269, 270, 271]. An integral feedback control can theoretically achieve perfect setpoint control, thus ideal for hormone homeostasis. Its execution requires either an exact zero‐order or antithetic process, both of which are difficult to achieve in biological systems where such processes tend to be leaky, that is, they only exist in idealized conditions. Therefore, except for a few synthetic biological circuit examples, whether integral control operates in pure form in nature remains to be determined [272, 273]. In contrast, proportional feedback regulations with high loop gains, as we examined here for T3 mediated amplified feedback inhibition of TRH and TSH, are much more common in biological systems [27, 28, 29, 30, 32].

### 5.4 Comparison With Other Endocrine Feedback Systems

The feedback structure and amplification configuration as reviewed here is not limited to the HPT system. Parathyroid hormone (PTH) and serum calcium are also inversely correlated with calcium within a 2‐fold range and PTH spanning about 2 orders of magnitude [274, 275, 276]. This inverse logPTH‐calcium relationship resembles the logTSH‐T4 relationship, highly suggesting the existence of signal amplification in the feedback regulation of PTH to maintain serum calcium homeostasis. In contrast, for an endocrine system that frequently requires allostatic regulation, such as the hypothalamic–pituitary–adrenal (HPA) axis, the circulating adrenocorticotropic hormone (ACTH) and cortisol levels are positively correlated and both vary in a comparable 10‐fold range [57, 58]. This relationship suggests that the variability of the HPA axis stems in the brain and no signal amplification exists in the adrenal gland.

### 5.5 Implementation of Negative Feedback Amplification in Existing Mathematical HPT Models

While for TSH‐stimulated thyroid response, linear or Michaelis–Menten kinetics tend to be used to describe T4 and T3 production [277, 278, 279, 280], the negative feedback in the HPT axis has been modeled in a variety of ways in thyroid modeling literature [281]. The early work by DiStefano III et al. used a negative linear term to describe the TH feedback on TSH [279]. Given the log‐linear relationship between TSH and fT4, DiStefano III and colleagues later adopted a mathematically equivalent inverse exponential function in their models to describe the inhibition of TSH secretion by T3 in the brain [282, 283, 284]:

$$\mathrm{TSH}\propto e^{-\text{T3}}$$

Work by Goede and colleagues also employed the inverse exponential function with slight modification to describe the production rate or steady‐state level of TSH with respect to fT4:

$\mathrm{TSH}\propto e^{-\varphi \text{fT4}},$ where *φ* is the slope of the exponential coefficient [48, 51, 59, 277]. Goede et al. further showed that the log‐linear function can be used to fit clinical TSH‐fT4 data that were repeatedly obtained from the same individuals [48, 59].

For an exponential function like:

$$\mathrm{TSH}\propto e^{-\varphi \mathrm{TH}},$$

the percentage change of TSH in response to a small percentage change of TH, that is, the logarithmic gain, is as follows:

$$\frac{\text{dTSH}/\mathrm{TSH}}{\mathrm{dTH}/\mathrm{TH}}=-\mathrm{\varphi TH}.$$

The gain is not a constant but increases linearly with TH, and whether its absolute value is greater than unity that enables signal amplification depends on both φ and TH values. This contrasts with an alternative linear logTSH‐logTH approach, where the logarithmic gain is constant. Since the TSH level can span a couple of orders of magnitude, and fT4 is generally held within a much narrower range, a logTSH‐logfT4 relationship may not be readily distinct from the logTSH‐fT4 relationship in practice given all the variations in hormone measurements. Further modifications of the log‐linear relationship have also been made. For instance, an inverse exponential‐power function, TSH ∝ e^−*φ*(fT4)2^, was used to describe the feedback [285]. In a recent HPT model, the TRH production is regulated by hypothalamic T3 following an inverse exponential function similar to the one above but a power law term fT4^n^ is multiplied when the blood fT4 rises above a predefined level [286]. Compared with the pure logTSH‐fT4 relationship, the amplification gain in the latter two cases is further increased by the power terms.

DiStefano III et al. used the simple inverse exponential for the feedback function of T3 on TSH to build a THYROISIM app that predicts the potential health risks of over‐the‐counter thyroid supplements [287]. While this works for euthyroid and mildly hypothyroid patients, the predicted TSH levels are unrealistically high for more severe hypothyroid conditions. Therefore, in their most recent personalized version of the model (p‐THYROSIM), the exponential function was replaced by an inhibitory Hill function with the Hill coefficient *n* = 6.29, as we did in Design A in Section 4 [288].

In comparison, Dietrich and colleagues have developed a series of HPT axis models by using the Michaelis–Menten (MiME) kinetics and non‐competitive divisive inhibition (NoCoDI) to describe the T3 feedback inhibition of TRH‐stimulated secretion of TSH [280, 289, 290, 291]. As a matter of fact, the MiME‐NoCoDI is equivalent to the TSH production term in Equation (2) of our model in Section 4 when the two Hill coefficients *n*_3_ and *n*_7_ are set to unity. However, under this condition no amplification can be expected for either the inhibition of TSH by TH or stimulation of TSH by TRH when each regulation is examined alone. But collectively, the two converging regulations can still provide some small degree of amplification. In a more recent model focusing on HPT homeostasis and allostasis, a multiplicative term of fT4 and fT3 was applied to describe the simultaneous inhibition of both TRH and TSH by the two THs [278]. Under this circumstance, some degree of feedback amplification can be expected, which seems to contribute to the insensitivity of fT3 to altered T4 production predicted by the model.

Leow and Goede used the first derivative of the TSH‐fT4 relationship to describe the gain of each of the two arms of the feedback loop and the product of the two gains as the loop gain [59]. They further argued that the maximal loop gain is associated with the maximal curvature point of the TSH‐fT4 curve and designated it as the steady‐state setpoint, a postulation that could be attributed to their choosing fT4 ∝ 1‐e^−*α*TSH^ to describe TSH‐stimulated T4 production. By using a model where the fT4 feedback arm follows an inverse exponential function and the TSH stimulation of T4 arm follows the Michaelis–Menten kinetics, Goede conducted an HPT loop gain analysis by using the product of the two first derivatives of the fT4 vs. TSH relationship in the above two arms [277]. They showed the gain varies depending on the TSH and fT4 values, though it is generally greater than unity. Because the two local gains change in opposite directions as TSH and fT4 vary, there is a maximum gain.

Recently Alon's group has developed an HPT axis model, where, in addition to describing the negative feedback of TH on TRH and TSH as an inverse function of the respective production terms of the two neuroendocrine hormones, TSH‐stimulated thyroid gland growth and TH‐inhibited pituitary thyrotrope growth are implemented [292]. The latter two growth regulations provide integral feedback mechanisms that allow the model to achieve perfect setpoint control against perturbations so long as the thyroid and thyrotrope growth has not reached their respective carrying capacities. In a subsequent study of endocrine system design principles, they further examined the functions of a cascade circuit motif such as the HPT axis [293]. In addition to increasing the speed of response to allostatic demand, they argued that the intermediate organ anterior pituitary is needed to provide molecular abundance amplification. It is because the small number of TRH neurons, due to spatial restriction in the hypothalamus, cannot secrete enough TRH molecules to directly serve the distant thyroid gland. The anterior pituitary, containing nearly two order of magnitude more thyrotropes than TRH neurons, can relay the TRH signal such that enough amount of TSH molecules can be secreted to the systemic circulation to stimulate the thyroid gland. Note that this molecular abundance or concentration amplification from TRH to TSH is different from the percentage amplification as we discussed in this article.

In summary, the way THs exert their feedback inhibition on TRH and TSH has been modeled with a variety of mathematical formulism, each furnishing a different modality of feedback gain if existing. How different implementations can lead to different performances in the homeostatic and allostatic regulation of THs as well as their dynamic response to perturbations at different sites warrants further studies.

### 5.6 Complexities of Regulations of TRH and TSH

Despite most of the molecular signaling events described above working to enhance the feedback gain of the HPT axis to amplify the TSH response to TRH and T4/T3, there are regulatory events in the hypothalamus and AP that are not necessarily concordant with this task. Some of them may attenuate the feedback gain while carrying out other functions, reflecting the complexity of the HPT axis' design.

#### 5.6.1 Downregulation of TRHR by TRH

As with many GPCRs, a number of studies indicated that TRH may stimulate the downregulation of its own receptor, TRHR, via multiple mechanisms. In mouse pituitary thyrotropic tumor cells, TRH downregulated the number of TRHR in a concentration‐dependent manner, although not their affinity for TRH [202]. Downregulation of TRHR and thus desensitization of TRH signaling can be mediated by receptor internalization upon phosphorylation by GPCR kinase 2 (GRK2) and subsequent binding by β‐arrestin [294]. In rat pituitary GH_3_ cells, TRH decreased the TRHR mRNA level. Moreover, in GH_3_ cells stably transfected with mouse TRHR cDNA, TRH increased the rate of TRHR mRNA degradation, by involving PKC as a potential mechanism [295, 296]. Incubating GH4C1 rat pituitary cells with TRH also caused a decrease in TRHR mRNA transcription, and intracellular Ca^2+^ and PKC seemed to play a role in this regard [297]. The functional significance of these transcriptional, posttranscriptional, and posttranslational downregulations of TRHR stimulated by TRH is not clear. Such regulation may be related to maintaining the sensitivity of the TSH response to pulsatile TRH stimulation; however, as a local NFL, it may reduce the amplification gain in the pituitary.

#### 5.6.2 Local Feedforward and Feedback Regulations Between TRH and Tanycytes

Besides TH‐mediated feedback induction of PPII that regulates postsecretory TRH bioavailability in the ME, TRH access to the portal capillaries is also autoregulated, in a feedforward manner. It has been reported in mice that TRH released from the neuronal terminals in the ME activated Gα_q/11_‐coupled TRHR1 in the β‐tanycytes, resulting in increased PLC and IP_3_ production and Ca^2+^ concentration [298]. Activation of this signaling pathway then increased the size of the tanycytic end feet terminating on the surface of the fenestrated portal vessels, thus reducing the transport of TRH to the pituitary. In addition, activation of the TRHR1 pathway in the tanycytes seemed to augment the enzymatic activity of PPII to degrade TRH [298]. Therefore, a tanycyte‐mediated incoherent feedforward regulation appears to exist that reduces the TRH bioavailability for the pituitary when it is too high.

More recently, a reciprocal microcircuit between the β‐tanycytes and TRH axons in the external zone of the ME was reported in mice [299]. The TRH axons release glutamate as a cotransmitter, which can stimulate, via kainite and AMPA receptors, the expression of diacylglycerol lipase α (DAGLα), a primary synthesizing enzyme for endocannabinoid 2‐arachinodonoylglycerol (2‐AG) in tanycytes to induce endocannabinoid synthesis and release. The released 2‐AG binds to type‐I cannabinoid receptor (CB1) on the TRH axons and inhibits TRH release. Therefore, it appears evident that a local autoregulatory negative feedback loop between tanycytes and TRH neurons exists and acts to restrict TRH release in the ME. The physiological functions of this autoregulatory loop and the feedforward regulation described above, both involving β‐tanycytes, are unclear and may be related to TRH pulsatility [298, 299, 300]. TRH‐stimulated TSH pulsatile secretion may be critical to TSHR signal transduction and TH release in the thyroid gland, at least in mice [301]. Conversely, the existence of these local regulations may dampen the overall feedback gain of the HPT axis.

#### 5.6.3 Short Feedback Regulation of Tanycytes by TSH

Most recently, it was shown that tanycytes in the rodent MBH express TSHR and their DIO2, DIO3, and OATP1c1 mRNA levels can be upregulated by TSH [140]. These findings suggest a TSH‐dependent feedback regulation of local T3 production and metabolism in this part of the hypothalamus. As this short feedback‐regulated T3 may transport retrogradely to the PVN to inhibit TRH, it adds further complexity to the HPT axis.

#### 5.6.4 Ultrashort Feedback of TSH

There is also sufficient evidence supporting the existence of a TSH ultrashort feedback loop (also known as Brokken‐Wiersinga‐Prummel feedback loop) that has a critical role in regulating TSH secretion in the pituitary [302]. This autoregulation was demonstrated in studies showing that human TSH (hTSH) can inhibit TRH‐stimulated rabbit TSH (rTSH) secretion as fast as 10 min after its injection in thyroidectomized hypothyroid rabbits as well as in rabbit pituitary cells [303, 304]. Further studies demonstrated that TSHR is expressed in the folliculo‐stellate cells in the human AP and murine folliculo‐stellate cell line [305, 306, 307], which mediates the autoregulation of TSH. The biological function of this ultrashort feedback loop may be to prevent excessively high TSH secretion and generate TSH pulses [302].

### 5.7 Implications in Clinical Endocrinology and Environmental Health

Precision medicine on thyroid patients has been pursued with intent to achieve personalized clinical outcomes [11, 308]. Several attempts have been made through mathematical modeling of the HPT axis with the TH‐mediated feedback loop. For instance, the log‐linear relationship between TSH and fT4 was utilized to model the individual TSH response to LT4 therapy in thyroidectomized patients, which can speed up their dosing adjustment by 1–2 months [309]. The p‐THYROSIM model, which used the Hill function to describe the feedback gain, was developed to optimize T4 dosing for male and female hypothyroid patients customized to their BMIs [288]. An HPT feedback model was used to help determine whether T4 monotherapy or T4 + T3 combined therapy is optimal for different DIO1 and DIO2 genetic polymorphisms [310]. More recently, Geode proposed using the steady‐state approach to optimize LT4 dosing to help patients reach homeostasis within 4 weeks [311]. In the future, as our quantitative understanding of the HPT feedback system considerably improves, a digital twin of the HPT axis of individual thyroid patients can be developed to guide clinical practice with high precision.

Humans are constantly exposed to numerous environmental pollutants, some of which are endocrine‐disrupting chemicals (EDCs) of the thyroid system [312, 313, 314]. Some of the observed variations of THs may result from exposures to these thyroid EDCs. Depending on the structures and physiochemical properties, EDCs may target different proteins as the molecular initiating events (MIEs) in the HPT axis and peripheral TH‐regulated tissues [315, 316]. While most EDCs may act outside of the brain, some may also disrupt the HPT axis centrally. By design, the HPT axis is better equipped to resist perturbations that directly interfere with TH synthesis, secretion, and metabolism as well as TSH clearance, yet it is more prone to perturbations by EDCs that can access the brain and act at MIE sites upstream of the amplifier in the hypothalamus and AP. Therefore, centrally acting thyroid EDCs may be of high concern. Developing quantitative adverse outcome pathway (qAOP) models of the human HPT axis and accounting for individual variabilities with properly modeled feedback gains can mechanistically predict nonlinear dose responses and help support population‐based health risk assessment of thyroid EDCs [317, 318, 319, 320].

## 6 Closing Remarks

Nature seems to follow a design principle that minimizes the perturbation of TH levels by placing the signal amplifier of the HPT feedback loop in the brain. In this review we identify a suite of potential URMs that may collectively amplify the inhibitory actions of THs on TRH and TSH. Often treated in the biology literature as redundancy, these multistep regulatory mechanisms increase biological robustness such that if one pathway fails, the system can still operate largely unaffected. True to this line of thinking, these multistep pathways have also emerged to confer a high loop gain to the HPT axis, where a small percentage change in the circulating fT4/fT3 can be amplified to control a much larger percentage change in the circulating TSH in the opposite direction. The nature's design principle revealed here enhances our cross‐scale understanding of the systems biology of the HPT axis as a dynamic control system. Knowledge gained on the homeostatic and allostatic regulation of THs can promote thyroid medicine and risk assessment of environmental thyroid disruptors with increased precision.

## Author Contributions

Li Jing: conceptualization, writing – original draft (lead), review and editing. Sarahna A. Moyd: writing – original draft, review and editing. Qiang Zhang: conceptualization (lead); formal analysis; writing – original draft (lead), review and editing; supervision; funding acquisition.

## Funding

This research was supported in part by NIEHS Superfund Research grant P42ES004911.

## Disclosure

The authors have nothing to report.

## Ethics Statement

The authors have nothing to report.

## Conflicts of Interest

The authors declare no conflicts of interest.

## Data Availability

Data sharing not applicable to this article as no datasets were generated or analysed during the current study.

## References

[1] R. T. Zoeller and J. Rovet , “Timing of Thyroid Hormone Action in the Developing Brain: Clinical Observations and Experimental Findings,” Journal of Neuroendocrinology 16, no. 10 (2004): 809–818. 15500540 10.1111/j.1365-2826.2004.01243.x

[2] M. Gomberg‐Maitland and W. H. Frishman , “Thyroid Hormone and Cardiovascular Disease,” American Heart Journal 135, no. 2 Pt 1 (1998): 187–196. 9489964 10.1016/s0002-8703(98)70081-x

[3] E. Hatziagelaki , S. A. Paschou , M. Schön , T. Psaltopoulou , and M. Roden , “NAFLD and Thyroid Function: Pathophysiological and Therapeutic Considerations,” Trends in Endocrinology and Metabolism 33, no. 11 (2022): 755–768. 36171155 10.1016/j.tem.2022.08.001

[4] A. Amin , W. S. Dhillo , and K. G. Murphy , “The Central Effects of Thyroid Hormones on Appetite,” Journal of Thyroid Research 2011 (2011): 306510. 21687648 10.4061/2011/306510 PMC3112506

[5] C. E. Combs , J. J. Nicholls , J. H. Duncan Bassett , and G. R. Williams , “Thyroid Hormones and Bone Development,” Minerva Endocrinologica 36, no. 1 (2011): 71–85. 21460788

[6] A. C. Bianco and E. A. McAninch , “The Role of Thyroid Hormone and Brown Adipose Tissue in Energy Homoeostasis,” Lancet Diabetes & Endocrinology 1, no. 3 (2013): 250–258. 24622373 10.1016/S2213-8587(13)70069-X PMC4976626

[7] J. F. Silva , N. M. Ocarino , and R. Serakides , “Thyroid Hormones and Female Reproduction,” Biology of Reproduction 99, no. 5 (2018): 907–921. 29767691 10.1093/biolre/ioy115

[8] R. Mazzilli , S. Medenica , A. M. Di Tommaso , et al., “The Role of Thyroid Function in Female and Male Infertility: A Narrative Review,” Journal of Endocrinological Investigation 46, no. 1 (2023): 15–26. 35945393 10.1007/s40618-022-01883-7 PMC9829629

[9] K. J. Welsh and S. J. Soldin , “DIAGNOSIS OF ENDOCRINE DISEASE: How Reliable Are Free Thyroid and Total T3 Hormone Assays?,” European Journal of Endocrinology 175, no. 6 (2016): R255–r263. 27737898 10.1530/EJE-16-0193 PMC5113291

[10] R. Jain , “Thyroid Profile of the Reference United States Population: Data From NHANES 2007‐2012,” International Archives of Endocrinology Clinical Research 1, no. 1 (2015): 1–8.

[11] S. Andersen , K. M. Pedersen , N. H. Bruun , and P. Laurberg , “Narrow Individual Variations in Serum T4 and T3 in Normal Subjects: A Clue to the Understanding of Subclinical Thyroid Disease,” Journal of Clinical Endocrinology & Metabolism 87, no. 3 (2002): 1068–1072. 11889165 10.1210/jcem.87.3.8165

[12] C. Serrano‐Nascimento and M. T. Nunes , “Perchlorate, Nitrate, and Thiocyanate: Environmental Relevant NIS‐Inhibitors Pollutants and Their Impact on Thyroid Function and Human Health,” Frontiers in Endocrinology 13 (2022): 995503. 36339434 10.3389/fendo.2022.995503 PMC9633673

[13] M. Babić Leko , I. Gunjača , N. Pleić , and T. Zemunik , “Environmental Factors Affecting Thyroid‐Stimulating Hormone and Thyroid Hormone Levels,” International Journal of Molecular Sciences 22, no. 12 (2021): 6521. 34204586 10.3390/ijms22126521 PMC8234807

[14] M. P. Rayman , “Multiple Nutritional Factors and Thyroid Disease, With Particular Reference to Autoimmune Thyroid Disease,” Proceedings of the Nutrition Society 78, no. 1 (2019): 34–44. 30208979 10.1017/S0029665118001192

[15] C. E. Citterio , C. M. Rivolta , and H. M. Targovnik , “Structure and Genetic Variants of Thyroglobulin: Pathophysiological Implications,” Molecular and Cellular Endocrinology 528 (2021): 111227. 33689781 10.1016/j.mce.2021.111227

[16] A. H. van der Spek , E. Fliers , and A. Boelen , “The Classic Pathways of Thyroid Hormone Metabolism,” Molecular and Cellular Endocrinology 458 (2017): 29–38. 28109953 10.1016/j.mce.2017.01.025

[17] C. Luongo , M. Dentice , and D. Salvatore , “Deiodinases and Their Intricate Role in Thyroid Hormone Homeostasis,” Nature Reviews Endocrinology 15, no. 8 (2019): 479–488. 10.1038/s41574-019-0218-2 31160732

[18] J. E. Silva and P. R. Larsen , “Pituitary Nuclear 3,5,3′‐Triiodothyronine and Thyrotropin Secretion: An Explanation for the Effect of Thyroxine,” Science 198, no. 4317 (1977): 617–620. 199941 10.1126/science.199941

[19] T. L. Fonseca , M. Correa‐Medina , M. P. Campos , et al., “Coordination of Hypothalamic and Pituitary T3 Production Regulates TSH Expression,” Journal of Clinical Investigation 123, no. 4 (2013): 1492–1500. 23524969 10.1172/JCI61231 PMC3613903

[20] G. C. Schussler , “The Thyroxine‐Binding Proteins,” Thyroid 10, no. 2 (2000): 141–149. 10718550 10.1089/thy.2000.10.141

[21] C. Fekete and R. M. Lechan , “Central Regulation of Hypothalamic‐Pituitary‐Thyroid axis Under Physiological and Pathophysiological Conditions,” Endocrine Reviews 35, no. 2 (2014): 159–194. 24423980 10.1210/er.2013-1087 PMC3963261

[22] U. Feldt‐Rasmussen , G. Effraimidis , and M. Klose , “The Hypothalamus‐Pituitary‐Thyroid (HPT)‐axis and Its Role in Physiology and Pathophysiology of Other Hypothalamus‐Pituitary Functions,” Molecular and Cellular Endocrinology 525 (2021): 111173. 33549603 10.1016/j.mce.2021.111173

[23] S. J. Richardson , R. C. Wijayagunaratne , D. G. D'Souza , V. M. Darras , and S. L. J. Van Herck , “Transport of Thyroid Hormones via the Choroid Plexus Into the Brain: The Roles of Transthyretin and Thyroid Hormone Transmembrane Transporters,” Frontiers in Neuroscience 9 (2015): 66. 25784853 10.3389/fnins.2015.00066 PMC4347424

[24] E. Fliers , A. Alkemade , W. M. Wiersinga , and D. F. Swaab , “Hypothalamic Thyroid Hormone Feedback in Health and Disease,” in Progress in Brain Research, vol. 153 (Elsevier, 2006), 189–207. 16876576 10.1016/S0079-6123(06)53011-0

[25] M. I. Chiamolera and F. E. Wondisford , “Minireview: Thyrotropin‐Releasing Hormone and the Thyroid Hormone Feedback Mechanism,” Endocrinology 150, no. 3 (2009): 1091–1096. 19179434 10.1210/en.2008-1795

[26] A. N. Hollenberg , “The Role of the Thyrotropin‐Releasing Hormone (TRH) Neuron as a Metabolic Sensor,” Thyroid 18, no. 2 (2008): 131–139. 18279013 10.1089/thy.2007.0251

[27] S. Liu , J. Pi , and Q. Zhang , “Signal Amplification in the KEAP1‐NRF2‐ARE Antioxidant Response Pathway,” Redox Biology 54 (2022): 102389. 35792437 10.1016/j.redox.2022.102389 PMC9287733

[28] J. E. Ferrell, Jr. and S. H. Ha , “Ultrasensitivity Part III: Cascades, Bistable Switches, and Oscillators,” Trends in Biochemical Sciences 39, no. 12 (2014): 612–618. 25456048 10.1016/j.tibs.2014.10.002 PMC4254632

[29] F. Montefusco , A. Procopio , I. M. Bulai , F. Amato , and C. Cosentino , “Role of Ultrasensitivity in Biomolecular Circuitry for Achieving Homeostasis,” Nonlinear Dynamics 112, no. 7 (2024): 5635–5662.

[30] Q. Zhang and M. E. Andersen , “Dose Response Relationship in Anti‐Stress Gene Regulatory Networks,” PLoS Computational Biology 3, no. 3 (2007): e24. 17335342 10.1371/journal.pcbi.0030024 PMC1808489

[31] H. El‐Samad , “Biological Feedback Control—Respect the Loops,” Cell Systems 12, no. 6 (2021): 477–487. 34139160 10.1016/j.cels.2021.05.004

[32] C. Cuba Samaniego and E. Franco , “Ultrasensitive Molecular Controllers for Quasi‐Integral Feedback,” Cell Systems 12, no. 3 (2021): 272–288.e273. 33539724 10.1016/j.cels.2021.01.001

[33] Q. Zhang , S. Bhattacharya , and M. E. Andersen , “Ultrasensitive Response Motifs: Basic Amplifiers in Molecular Signalling Networks,” Open Biology 3, no. 4 (2013): 130031. 23615029 10.1098/rsob.130031 PMC3718334

[34] J. E. Ferrell, Jr. and S. H. Ha , “Ultrasensitivity Part I: Michaelian Responses and Zero‐Order Ultrasensitivity,” Trends in Biochemical Sciences 39, no. 10 (2014): 496–503. 25240485 10.1016/j.tibs.2014.08.003 PMC4214216

[35] J. E. Ferrell, Jr. and S. H. Ha , “Ultrasensitivity Part II: Multisite Phosphorylation, Stoichiometric Inhibitors, and Positive Feedback,” Trends in Biochemical Sciences 39, no. 11 (2014): 556–569. 25440716 10.1016/j.tibs.2014.09.003 PMC4435807

[36] R. Hoermann , W. Eckl , C. Hoermann , and R. Larisch , “Complex Relationship Between Free Thyroxine and TSH in the Regulation of Thyroid Function,” European Journal of Endocrinology 162, no. 6 (2010): 1123–1129. 20299491 10.1530/EJE-10-0106

[37] H. E. van Deventer , D. R. Mendu , A. T. Remaley , and S. J. Soldin , “Inverse Log‐Linear Relationship Between Thyroid‐Stimulating Hormone and Free Thyroxine Measured by Direct Analog Immunoassay and Tandem Mass Spectrometry,” Clinical Chemistry 57, no. 1 (2011): 122–127. 21097676 10.1373/clinchem.2010.154088

[38] C. A. Spencer , J. S. Lopresti , A. Patel , et al., “Applications of a New Chemiluminometric Thyrotropin Assay to Subnormal Measurement*,” Journal of Clinical Endocrinology & Metabolism 70, no. 2 (1990): 453–460. 2105333 10.1210/jcem-70-2-453

[39] N. C. Hadlow , K. M. Rothacker , R. Wardrop , S. J. Brown , E. M. Lim , and J. P. Walsh , “The Relationship Between TSH and Free T_4_ in a Large Population Is Complex and Nonlinear and Differs by Age and Sex,” Journal of Clinical Endocrinology and Metabolism 98, no. 7 (2013): 2936–2943. 23671314 10.1210/jc.2012-4223

[40] K. M. Rothacker , S. J. Brown , N. C. Hadlow , R. Wardrop , and J. P. Walsh , “Reconciling the Log‐Linear and Non–Log‐Linear Nature of the TSH‐Free T4 Relationship: Intra‐Individual Analysis of a Large Population,” Journal of Clinical Endocrinology & Metabolism 101, no. 3 (2016): 1151–1158. 26735261 10.1210/jc.2015-4011

[41] P. M. Clark , R. L. Holder , S. M. Haque , F. D. Hobbs , L. M. Roberts , and J. A. Franklyn , “The Relationship Between Serum TSH and Free T4 in Older People,” Postgraduate Medical Journal 88, no. 1045 (2012): 668–670. 23097057 10.1136/postgradmedj-2011-200433rep

[42] J. Jonklaas , N. Kahric‐Janicic , O. P. Soldin , and S. J. Soldin , “Correlations of Free Thyroid Hormones Measured by Tandem Mass Spectrometry and Immunoassay With Thyroid‐Stimulating Hormone Across 4 Patient Populations,” Clinical Chemistry 55, no. 7 (2009): 1380–1388. 19460839 10.1373/clinchem.2008.118752 PMC3633598

[43] S. J. Brown , A. P. Bremner , N. C. Hadlow , et al., “The Log TSH–Free T4 Relationship in a Community‐Based Cohort Is Nonlinear and Is Influenced by Age, Smoking and Thyroid Peroxidase Antibody Status,” Clinical Endocrinology 85, no. 5 (2016): 789–796. 27197788 10.1111/cen.13107

[44] S. Reichlin and R. D. Utiger , “Regulation of the Pituitary‐Thyroid axis in Man: Relationship of TSH Concentration to Concentration of Free and Total Thyroxine in Plasma,” Journal of Clinical Endocrinology and Metabolism 27, no. 2 (1967): 251–255. 4163614 10.1210/jcem-27-2-251

[45] S. L. Goede and M. K. Leow , “Letter to the Editor: The Ultimate Proof of the Log‐Linear Nature of TSH‐Free T4 Relationship by Intraindividual Analysis of a Large Population,” Journal of Clinical Endocrinology and Metabolism 101, no. 5 (2016): L57–L58. 27163472 10.1210/jc.2016-1439

[46] H. Alkhalaileh , R. Wei , J. K. Y. Lee , J. Jones , and J. Li , “Relationship Between TSH and Free Thyroxine in Outpatient cancer Patient Population,” Endocrine 82, no. 2 (2023): 319–325. 37286745 10.1007/s12020-023-03399-3

[47] N. Benhadi , E. Fliers , T. J. Visser , J. B. Reitsma , and W. M. Wiersinga , “Pilot Study on the Assessment of the Setpoint of the Hypothalamus‐Pituitary‐Thyroid axis in Healthy Volunteers,” European Journal of Endocrinology 162, no. 2 (2010): 323–329. 19926783 10.1530/EJE-09-0655

[48] S. L. Goede , M. K. Leow , J. W. Smit , and J. W. Dietrich , “A Novel Minimal Mathematical Model of the Hypothalamus‐Pituitary‐Thyroid axis Validated for Individualized Clinical Applications,” Mathematical Biosciences 249 (2014): 1–7. 24480737 10.1016/j.mbs.2014.01.001

[49] S. L. Goede and M. K. S. Leow , “The Effects of Triiodothyronine on the Free Thyroxine Set Point Position in the Hypothalamus Pituitary Thyroid Axis,” Acta Biotheoretica 72, no. 3 (2024): 10. 39207534 10.1007/s10441-024-09486-w

[50] C. A. Meier , M. N. Maisey , A. Lowry , J. Müller , and M. A. Smith , “Interindividual Differences in the Pituitary‐Thyroid axis Influence the Interpretation of Thyroid Function Tests,” Clinical Endocrinology 39, no. 1 (1993): 101–107. 8348700 10.1111/j.1365-2265.1993.tb01758.x

[51] S. L. Goede and M. K.‐S. Leow , “General Error Analysis in the Relationship Between Free Thyroxine and Thyrotropin and Its Clinical Relevance,” Computational and Mathematical Methods in Medicine 2013 (2013): 831275. 24082916 10.1155/2013/831275 PMC3780511

[52] J. P. Walsh , “Thyroid Function Across the Lifespan: Do Age‐Related Changes Matter?,” Endocrinology and Metabolism (Seoul) 37, no. 2 (2022): 208–219. 10.3803/EnM.2022.1463 PMC9081302 35417936

[53] M. Medici , W. E. Visser , T. J. Visser , and R. P. Peeters , “Genetic Determination of the Hypothalamic‐Pituitary‐Thyroid Axis: Where Do we Stand?,” Endocrine Reviews 36, no. 2 (2015): 214–244. 25751422 10.1210/er.2014-1081

[54] P. S. Hansen , T. H. Brix , T. I. Sørensen , K. O. Kyvik , and L. Hegedüs , “Major Genetic Influence on the Regulation of the Pituitary‐Thyroid Axis: A Study of Healthy Danish Twins,” Journal of Clinical Endocrinology and Metabolism 89, no. 3 (2004): 1181–1187. 15001606 10.1210/jc.2003-031641

[55] S. P. Fitzgerald and N. G. Bean , “The Relationship Between Population T4/TSH Set Point Data and T4/TSH Physiology,” Journal of Thyroid Research 2016 (2016): 6351473. 27123359 10.1155/2016/6351473 PMC4830732

[56] R. Hoermann , A. S. Cheung , M. Milne , and M. Grossmann , “Hypothalamic‐Pituitary‐Thyroid Axis Set Point Alterations Are Associated With Body Composition in Androgen‐Deprived Men,” Journal of the Endocrine Society 1, no. 7 (2017): 874–885. 29264538 10.1210/js.2017-00057 PMC5686654

[57] M. Daimon , A. Kamba , H. Murakami , et al., “Association Between Pituitary‐Adrenal Axis Dominance Over the Renin‐Angiotensin‐Aldosterone System and Hypertension,” Journal of Clinical Endocrinology & Metabolism 101, no. 3 (2016): 889–897. 26731257 10.1210/jc.2015-3568

[58] H. Bando , C. Y. Zhang , Y. Takada , H. Takahashi , R. Yamasaki , and S. Saito , “Correlation Between Plasma Levels of ACTH and Cortisol in Basal States and During the CRH Test in Normal Subjects and Patients With Hypothalamo‐Pituitary Disorders,” Tokushima Journal of Experimental Medicine 38, no. 3–4 (1991): 61–69. 1668824

[59] M. K.‐S. Leow and S. L. Goede , “The Homeostatic Set Point of the Hypothalamus‐Pituitary‐Thyroid Axis—Maximum Curvature Theory for Personalized Euthyroid Targets,” Theoretical Biology and Medical Modelling 11, no. 1 (2014): 35. 25102854 10.1186/1742-4682-11-35 PMC4237899

[60] S. J. Ferrara , D. Bourdette , and T. S. Scanlan , “Hypothalamic‐Pituitary‐Thyroid Axis Perturbations in Male Mice by CNS‐Penetrating Thyromimetics,” Endocrinology 159, no. 7 (2018): 2733–2740. 29846550 10.1210/en.2018-00065 PMC6457038

[61] L. Jing and Q. Zhang , “Intrathyroidal Feedforward and Feedback Network Regulating Thyroid Hormone Synthesis and Secretion,” Frontiers in Endocrinology (Lausanne) 13 (2022): 992883. 10.3389/fendo.2022.992883 PMC9519864 36187113

[62] C. M. Kumbale , E. O. Voit , and Q. Zhang , “Emergence and Enhancement of Ultrasensitivity Through Posttranslational Modulation of Protein Stability,” Biomolecules 11, no. 11 (2021): 1741. 34827739 10.3390/biom11111741 PMC8615576

[63] C. Fekete and R. M. Lechan , “Negative Feedback Regulation of Hypophysiotropic Thyrotropin‐Releasing Hormone (TRH) Synthesizing Neurons: Role of Neuronal Afferents and Type 2 Deiodinase,” Frontiers in Neuroendocrinology 28, no. 2–3 (2007): 97–114. 17588648 10.1016/j.yfrne.2007.04.002 PMC2000455

[64] E. D. R. Arrojo , T. L. Fonseca , J. P. Werneck‐de‐Castro , and A. C. Bianco , “Role of the Type 2 Iodothyronine Deiodinase (D2) in the Control of Thyroid Hormone Signaling,” Biochimica et Biophysica Acta 1830, no. 7 (2013): 3956–3964. 22967761 10.1016/j.bbagen.2012.08.019 PMC4979226

[65] S. Mariotti and P. Beck‐Peccoz , “Physiology of the Hypothalamic‐Pituitary‐Thyroid Axis,” in Endotext [Internet] 2000–2023, ed. K. R. Feingold , B. Anawalt , M. R. Blackman , et al. (MDText.com, 2000), https://www.ncbi.nlm.nih.gov/books/NBK278958/.

[66] J. Sap , A. Muñoz , K. Damm , et al., “The c‐Erb‐A Protein Is a High‐Affinity Receptor for Thyroid Hormone,” Nature 324, no. 6098 (1986): 635–640. 2879242 10.1038/324635a0

[67] C. Weinberger , C. C. Thompson , E. S. Ong , R. Lebo , D. J. Gruol , and R. M. Evans , “The c‐Erb‐A Gene Encodes a Thyroid Hormone Receptor,” Nature 324, no. 6098 (1986): 641–646. 2879243 10.1038/324641a0

[68] S. Izumo and V. Mahdavi , “Thyroid Hormone Receptor Alpha Isoforms Generated by Alternative Splicing Differentially Activate Myosin HC Gene Transcription,” Nature 334, no. 6182 (1988): 539–542. 2841611 10.1038/334539a0

[69] M. B. Murray , N. D. Zilz , N. L. McCreary , M. J. MacDonald , and H. C. Towle , “Isolation and Characterization of Rat cDNA Clones for Two Distinct Thyroid Hormone Receptors,” Journal of Biological Chemistry 263, no. 25 (1988): 12770–12777. 2457590

[70] W. M. Wood , J. M. Dowding , B. R. Haugen , T. M. Bright , D. F. Gordon , and E. C. Ridgway , “Structural and Functional Characterization of the Genomic Locus Encoding the Murine Beta 2 Thyroid Hormone Receptor,” Molecular Endocrinology (Baltimore, md) 8, no. 12 (1994): 1605–1617. 7708051 10.1210/mend.8.12.7708051

[71] C. S. Anyetei‐Anum , V. R. Roggero , and L. A. Allison , “Thyroid Hormone Receptor Localization in Target Tissues,” Journal of Endocrinology 237, no. 1 (2018): R19–r34. 29440347 10.1530/JOE-17-0708 PMC5843491

[72] R. A. Hodin , M. A. Lazar , B. I. Wintman , et al., “Identification of a Thyroid Hormone Receptor That Is Pituitary‐Specific,” Science 244, no. 4900 (1989): 76–79. 2539642 10.1126/science.2539642

[73] J. B. Hahm and M. L. Privalsky , “Research Resource: Identification of Novel Coregulators Specific for Thyroid Hormone Receptor‐β2,” Molecular Endocrinology (Baltimore, md) 27, no. 5 (2013): 840–859. 23558175 10.1210/me.2012-1117 PMC3634115

[74] G. Kuroda , S. Sasaki , A. Matsushita , et al., “G ATA2 Mediates the Negative Regulation of the Prepro‐Thyrotropin‐Releasing Hormone Gene by Liganded T3 Receptor β2 in the Rat Hypothalamic Paraventricular Nucleus,” PLoS One 15, no. 11 (2020): e0242380. 33201916 10.1371/journal.pone.0242380 PMC7671546

[75] S. M. Dupré , H. Guissouma , F. Flamant , et al., “Both Thyroid Hormone Receptor (TR) Beta 1 and TR Beta 2 Isoforms Contribute to the Regulation of Hypothalamic Thyrotropin‐Releasing Hormone,” Endocrinology 145, no. 5 (2004): 2337–2345. 14726446 10.1210/en.2003-1209

[76] E. D. Abel , R. S. Ahima , M. E. Boers , J. K. Elmquist , and F. E. Wondisford , “Critical Role for Thyroid Hormone Receptor Beta2 in the Regulation of Paraventricular Thyrotropin‐Releasing Hormone Neurons,” Journal of Clinical Investigation 107, no. 8 (2001): 1017–1023. 11306605 10.1172/JCI10858 PMC199552

[77] D. Forrest , E. Hanebuth , R. J. Smeyne , et al., “Recessive Resistance to Thyroid Hormone in Mice Lacking Thyroid Hormone Receptor Beta: Evidence for Tissue‐Specific Modulation of Receptor Function,” EMBO Journal 15, no. 12 (1996): 3006–3015. 8670802 PMC450242

[78] R. E. Weiss , D. Forrest , J. Pohlenz , K. Cua , T. Curran , and S. Refetoff , “Thyrotropin Regulation by Thyroid Hormone in Thyroid Hormone Receptor beta‐Deficient Mice,” Endocrinology 138, no. 9 (1997): 3624–3629. 9275045 10.1210/endo.138.9.5412

[79] R. J. Koenig , M. A. Lazar , R. A. Hodin , et al., “Inhibition of Thyroid Hormone Action by a Non‐Hormone Binding c‐erbA Protein Generated by Alternative mRNA Splicing,” Nature 337, no. 6208 (1989): 659–661. 2537467 10.1038/337659a0

[80] J. Bernal and B. Morte , “Thyroid Hormone Receptor Activity in the Absence of Ligand: Physiological and Developmental Implications,” Biochimica et Biophysica Acta 1830, no. 7 (2013): 3893–3899. 22554916 10.1016/j.bbagen.2012.04.014

[81] B. R. Southwell , W. Duan , D. Alcorn , et al., “Thyroxine Transport to the Brain: Role of Protein Synthesis by the Choroid Plexus,” Endocrinology 133, no. 5 (1993): 2116–2126. 8404661 10.1210/endo.133.5.8404661

[82] R. M. Paragliola , A. Corsello , P. Concolino , et al., “Iodothyronine Deiodinases and Reduced Sensitivity to Thyroid Hormones,” Frontiers in Bioscience (Landmark Edition) 25 (2020): 201–228. 31585886 10.2741/4803

[83] I. Kakucska , W. Rand , and R. M. Lechan , “Thyrotropin‐Releasing Hormone Gene Expression in the Hypothalamic Paraventricular Nucleus Is Dependent Upon Feedback Regulation by Both Triiodothyronine and Thyroxine,” Endocrinology 130, no. 5 (1992): 2845–2850. 1572297 10.1210/endo.130.5.1572297

[84] M. J. Schneider , S. N. Fiering , S. E. Pallud , A. F. Parlow , D. L. St Germain , and V. A. Galton , “Targeted Disruption of the Type 2 Selenodeiodinase Gene (DIO2) Results in a Phenotype of Pituitary Resistance to T4,” Molecular Endocrinology 15, no. 12 (2001): 2137–2148. 11731615 10.1210/mend.15.12.0740

[85] R. M. Lechan and C. Fekete , “Role of Thyroid Hormone Deiodination in the Hypothalamus,” Thyroid 15, no. 8 (2005): 883–897. 16131331 10.1089/thy.2005.15.883

[86] A. Guadaño‐Ferraz , M. J. Obregón , D. L. S. Germain , and J. Bernal , “The Type 2 Iodothyronine Deiodinase Is Expressed Primarily in Glial Cells in the Neonatal Rat Brain,” National Academy of Sciences of the United States of America 94, no. 19 (1997): 10391–10396. 10.1073/pnas.94.19.10391 PMC23373 9294221

[87] P. N. Riskind , J. M. Kolodny , and P. R. Larsen , “The Regional Hypothalamic Distribution of Type II 5′‐Monodeiodinase in Euthyroid and Hypothyroid Rats,” Brain Research 420, no. 1 (1987): 194–198. 3676753 10.1016/0006-8993(87)90260-5

[88] H. M. Tu , S. W. Kim , D. Salvatore , et al., “Regional Distribution of Type 2 Thyroxine Deiodinase Messenger Ribonucleic Acid in Rat Hypothalamus and Pituitary and Its Regulation by Thyroid Hormone,” Endocrinology 138, no. 8 (1997): 3359–3368. 9231788 10.1210/endo.138.8.5318

[89] E. M. Rodríguez , J. L. Blázquez , F. E. Pastor , et al., “Hypothalamic Tanycytes: A Key Component of Brain‐Endocrine Interaction,” International Review of Cytology 247 (2005): 89–164. 16344112 10.1016/S0074-7696(05)47003-5

[90] A. Alkemade , E. C. Friesema , U. A. Unmehopa , et al., “Neuroanatomical Pathways for Thyroid Hormone Feedback in the Human Hypothalamus,” Journal of Clinical Endocrinology and Metabolism 90, no. 7 (2005): 4322–4334. 15840737 10.1210/jc.2004-2567

[91] A. Alkemade , E. C. Friesema , A. Kalsbeek , D. F. Swaab , T. J. Visser , and E. Fliers , “Expression of Thyroid Hormone Transporters in the Human Hypothalamus,” Journal of Clinical Endocrinology and Metabolism 96, no. 6 (2011): E967–E971. 21508134 10.1210/jc.2010-2750

[92] L. M. Roberts , K. Woodford , M. Zhou , et al., “Expression of the Thyroid Hormone Transporters Monocarboxylate Transporter‐8 (SLC16A2) and Organic Ion Transporter‐14 (SLCO1C1) at the Blood‐Brain Barrier,” Endocrinology 149, no. 12 (2008): 6251–6261. 18687783 10.1210/en.2008-0378

[93] F. Salas‐Lucia , C. Fekete , R. Sinkó , et al., “Axonal T3 Uptake and Transport Can Trigger Thyroid Hormone Signaling in the Brain,” eLife 12 (2023): e82683. 37204837 10.7554/eLife.82683 PMC10241515

[94] I. Kalló , P. Mohácsik , B. Vida , et al., “A Novel Pathway Regulates Thyroid Hormone Availability in Rat and Human Hypothalamic Neurosecretory Neurons,” PLoS One 7, no. 6 (2012): e37860. 22719854 10.1371/journal.pone.0037860 PMC3377717

[95] S. Diano , F. Naftolin , F. Goglia , and T. L. Horvath , “Fasting‐Induced Increase in Type II Iodothyronine Deiodinase Activity and Messenger Ribonucleic Acid Levels Is Not Reversed by Thyroxine in the Rat Hypothalamus,” Endocrinology 139, no. 6 (1998): 2879–2884. 9607797 10.1210/endo.139.6.6062

[96] J. P. Werneck de Castro , T. L. Fonseca , C. B. Ueta , et al., “Differences in Hypothalamic Type 2 Deiodinase Ubiquitination Explain Localized Sensitivity to Thyroxine,” Journal of Clinical Investigation 125, no. 2 (2015): 769–781. 25555216 10.1172/JCI77588 PMC4319436

[97] P. Joseph‐Bravo , L. Jaimes‐Hoy , R. M. Uribe , and J. L. Charli , “60 Years of Neuroendocrinology: TRH, the First Hypophysiotropic Releasing Hormone Isolated: Control of the Pituitary‐Thyroid axis,” Journal of Endocrinology 226, no. 2 (2015): T85–t100. 26101376 10.1530/JOE-15-0124

[98] E. M. Dyess , T. P. Segerson , Z. Liposits , et al., “Triiodothyronine Exerts Direct Cell‐Specific Regulation of Thyrotropin‐Releasing Hormone Gene Expression in the Hypothalamic Paraventricular Nucleus,” Endocrinology 123, no. 5 (1988): 2291–2297. 3139393 10.1210/endo-123-5-2291

[99] T. P. Segerson , J. Kauer , H. C. Wolfe , et al., “Thyroid Hormone Regulates TRH Biosynthesis in the Paraventricular Nucleus of the Rat Hypothalamus,” Science 238, no. 4823 (1987): 78–80. 3116669 10.1126/science.3116669

[100] M. L. Sugrue , K. R. Vella , C. Morales , M. E. Lopez , and A. N. Hollenberg , “The Thyrotropin‐Releasing Hormone Gene Is Regulated by Thyroid Hormone at the Level of Transcription In Vivo,” Endocrinology 151, no. 2 (2010): 793–801. 20032051 10.1210/en.2009-0976 PMC2817611

[101] N. G. Blake , M. R. Johnson , D. J. Eckland , O. J. Foster , and S. L. Lightman , “Effect of Food Deprivation and Altered Thyroid Status on the Hypothalamic‐Pituitary‐Thyroid axis in the Rat,” Journal of Endocrinology 133, no. 2 (1992): 183–188. 1613420 10.1677/joe.0.1330183

[102] E. D. Abel , M. E. Boers , C. Pazos‐Moura , et al., “Divergent Roles for Thyroid Hormone Receptor beta Isoforms in the Endocrine axis and Auditory System,” Journal of Clinical Investigation 104, no. 3 (1999): 291–300. 10430610 10.1172/JCI6397 PMC408418

[103] R. M. Lechan , Y. Qi , I. M. Jackson , and V. Mahdavi , “Identification of Thyroid Hormone Receptor Isoforms in Thyrotropin‐Releasing Hormone Neurons of the Hypothalamic Paraventricular Nucleus,” Endocrinology 135, no. 1 (1994): 92–100. 7516871 10.1210/endo.135.1.7516871

[104] D. J. Bradley , W. S. Young, 3rd , and C. Weinberger , “Differential Expression of Alpha and beta Thyroid Hormone Receptor Genes in Rat Brain and Pituitary,” Proceedings of the National Academy of Sciences of the United States of America 86, no. 18 (1989): 7250–7254. 2780568 10.1073/pnas.86.18.7250 PMC298035

[105] C. B. Cook , I. Kakucska , R. M. Lechan , and R. J. Koenig , “Expression of Thyroid Hormone Receptor Beta 2 in Rat Hypothalamus,” Endocrinology 130, no. 2 (1992): 1077–1079. 1733708 10.1210/endo.130.2.1733708

[106] H. Guissouma , M. S. Froidevaux , Z. Hassani , and B. A. Demeneix , “In Vivo siRNA Delivery to the Mouse Hypothalamus Confirms Distinct Roles of TR beta Isoforms in Regulating TRH Transcription,” Neuroscience Letters 406, no. 3 (2006): 240–243. 16930836 10.1016/j.neulet.2006.07.041

[107] A. N. Hollenberg , T. Monden , T. R. Flynn , M. E. Boers , O. Cohen , and F. E. Wondisford , “The Human Thyrotropin‐Releasing Hormone Gene Is Regulated by Thyroid Hormone Through Two Distinct Classes of Negative Thyroid Hormone Response Elements,” Molecular Endocrinology 9, no. 5 (1995): 540–550. 7565802 10.1210/mend.9.5.7565802

[108] M. Y. Díaz‐Gallardo , A. Cote‐Vélez , A. Carreón‐Rodríguez , J. L. Charli , and P. Joseph‐Bravo , “Phosphorylated Cyclic‐AMP‐Response Element‐Binding Protein and Thyroid Hormone Receptor Have Independent Response Elements in the Rat Thyrotropin‐Releasing Hormone Promoter: An Analysis in Hypothalamic Cells,” Neuroendocrinology 91, no. 1 (2010): 64–76. 19602869 10.1159/000228833

[109] T. Satoh , M. Yamada , T. Iwasaki , and M. Mori , “Negative Regulation of the Gene for the Preprothyrotropin‐Releasing Hormone From the Mouse by Thyroid Hormone Requires Additional Factors in Conjunction With Thyroid Hormone Receptors,” Journal of Biological Chemistry 271, no. 44 (1996): 27919–27926. 8910392 10.1074/jbc.271.44.27919

[110] S. Ishii , M. Yamada , T. Satoh , et al., “Aberrant Dynamics of Histone Deacetylation at the Thyrotropin‐Releasing Hormone Gene in Resistance to Thyroid Hormone,” Molecular Endocrinology 18, no. 7 (2004): 1708–1720. 15131262 10.1210/me.2004-0067

[111] M. F. Langlois , K. Zanger , T. Monden , J. D. Safer , A. N. Hollenberg , and F. E. Wondisford , “A Unique Role of the Beta‐2 Thyroid Hormone Receptor Isoform in Negative Regulation by Thyroid Hormone. Mapping of a Novel Amino‐Terminal Domain Important for Ligand‐Independent Activation,” Journal of Biological Chemistry 272, no. 40 (1997): 24927–24933. 9312095 10.1074/jbc.272.40.24927

[112] R. M. Lechan , P. Wu , I. M. Jackson , et al., “Thyrotropin‐Releasing Hormone Precursor: Characterization in Rat Brain,” Science 231, no. 4734 (1986): 159–161. 3079917 10.1126/science.3079917

[113] E. A. Nillni , “Neuroregulation of ProTRH Biosynthesis and Processing,” Endocrine 10, no. 3 (1999): 185–199. 10484283 10.1007/BF02738618

[114] E. A. Nillni , “Regulation of the Hypothalamic Thyrotropin Releasing Hormone (TRH) Neuron by Neuronal and Peripheral Inputs,” Frontiers in Neuroendocrinology 31, no. 2 (2010): 134–156. 20074584 10.1016/j.yfrne.2010.01.001 PMC2849853

[115] I. P. Cruz and E. A. Nillni , “Intracellular Sites of Prothyrotropin‐Releasing Hormone Processing,” Journal of Biological Chemistry 271, no. 37 (1996): 22736–22745. 8798448 10.1074/jbc.271.37.22736

[116] E. A. Nillni and K. A. Sevarino , “The Biology of Pro‐Thyrotropin‐Releasing Hormone‐Derived Peptides,” Endocrine Reviews 20, no. 5 (1999): 599–648. 10529897 10.1210/edrv.20.5.0379

[117] M. Perello and E. A. Nillni , “The Biosynthesis and Processing of Neuropeptides: Lessons From Prothyrotropin Releasing Hormone (proTRH),” Frontiers in Bioscience 12 (2007): 3554–3565. 17485321 10.2741/2334

[118] M. Yamada , S. Radovick , F. E. Wondisford , Y. Nakayama , B. D. Weintraub , and J. F. Wilber , “Cloning and Structure of Human Genomic DNA and Hypothalamic cDNA Encoding Human Prepro Thyrotropin‐Releasing Hormone,” Molecular Endocrinology 4, no. 4 (1990): 551–556. 2126343 10.1210/mend-4-4-551

[119] S. P. Smeekens and D. F. Steiner , “Identification of a Human Insulinoma cDNA Encoding a Novel Mammalian Protein Structurally Related to the Yeast Dibasic Processing Protease Kex2,” Journal of Biological Chemistry 265, no. 6 (1990): 2997–3000. 2154467

[120] N. G. Seidah , M. Marcinkiewicz , S. Benjannet , et al., “Cloning and Primary Sequence of a Mouse Candidate Prohormone Convertase PC1 Homologous to PC2, Furin, and Kex2: Distinct Chromosomal Localization and Messenger RNA Distribution in Brain and Pituitary Compared to PC2,” Molecular Endocrinology (Baltimore, md) 5, no. 1 (1991): 111–122. 2017186 10.1210/mend-5-1-111

[121] Y. Zhou and I. Lindberg , “Purification and Characterization of the Prohormone Convertase PC1(PC3),” Journal of Biological Chemistry 268, no. 8 (1993): 5615–5623. 8449925

[122] T. C. Friedman , Y. P. Loh , N. X. Cawley , et al., “Processing of Prothyrotropin‐Releasing Hormone (Pro‐TRH) by Bovine Intermediate Lobe Secretory Vesicle Membrane PC1 and PC2 Enzymes,” Endocrinology 136, no. 10 (1995): 4462–4472. 7664666 10.1210/endo.136.10.7664666

[123] E. Sánchez , J. L. Charli , C. Morales , et al., “Expression of the Proprotein Convertases PC1 and PC2 mRNAs in Thyrotropin Releasing Hormone Neurons of the Rat Paraventricular Nucleus of Hypothalamus,” Brain Research 761, no. 1 (1997): 77–86. 9247068 10.1016/s0006-8993(97)00280-1

[124] N. E. Cyr , R. C. Stuart , X. Zhu , D. F. Steiner , and E. A. Nillni , “Biosynthesis of proTRH‐Derived Peptides in Prohormone Convertase 1 and 2 Knockout Mice,” Peptides 35, no. 1 (2012): 42–48. 22421509 10.1016/j.peptides.2012.02.024

[125] M. Perello , R. Stuart , and E. A. Nillni , “Prothyrotropin‐Releasing Hormone Targets Its Processing Products to Different Vesicles of the Secretory Pathway,” Journal of Biological Chemistry 283, no. 29 (2008): 19936–19947. 18474603 10.1074/jbc.M800732200 PMC2459294

[126] E. A. Nillni , W. Xie , L. Mulcahy , V. C. Sanchez , and W. C. Wetsel , “Deficiencies in Pro‐Thyrotropin‐Releasing Hormone Processing and Abnormalities in Thermoregulation in Cpefat/Fat Mice,” Journal of Biological Chemistry 277, no. 50 (2002): 48587–48595. 12270926 10.1074/jbc.M206702200

[127] Q. L. Li , E. Jansen , G. A. Brent , S. Naqvi , J. F. Wilber , and T. C. Friedman , “Interactions Between the Prohormone Convertase 2 Promoter and the Thyroid Hormone Receptor,” Endocrinology 141, no. 9 (2000): 3256–3266. 10965896 10.1210/endo.141.9.7674

[128] X. Shen , Q. L. Li , G. A. Brent , and T. C. Friedman , “Thyroid Hormone Regulation of Prohormone Convertase 1 (PC1): Regional Expression in Rat Brain and In Vitro Characterization of Negative Thyroid Hormone Response Elements,” Journal of Molecular Endocrinology 33, no. 1 (2004): 21–33. 15291740 10.1677/jme.0.0330021

[129] X. Shen , Q. L. Li , G. A. Brent , and T. C. Friedman , “Regulation of Regional Expression in Rat Brain PC2 by Thyroid Hormone/Characterization of Novel Negative Thyroid Hormone Response Elements in the PC2 Promoter,” American Journal of Physiology. Endocrinology and Metabolism 288, no. 1 (2005): E236–E245. 15585599 10.1152/ajpendo.00144.2004

[130] M. Perello , T. Friedman , V. Paez‐Espinosa , X. Shen , R. C. Stuart , and E. A. Nillni , “Thyroid Hormones Selectively Regulate the Posttranslational Processing of Prothyrotropin‐Releasing Hormone in the Paraventricular Nucleus of the Hypothalamus,” Endocrinology 147, no. 6 (2006): 2705–2716. 16497799 10.1210/en.2005-1609

[131] V. P. Espinosa , M. Ferrini , X. Shen , K. Lutfy , E. A. Nillni , and T. C. Friedman , “Cellular Colocalization and Coregulation Between Hypothalamic Pro‐TRH and Prohormone Convertases in Hypothyroidism,” American Journal of Physiology. Endocrinology and Metabolism 292, no. 1 (2007): E175–E186. 16926379 10.1152/ajpendo.00288.2006

[132] J. M. Rondeel , W. J. de Greef , W. Klootwijk , and T. J. Visser , “Effects of Hypothyroidism on Hypothalamic Release of Thyrotropin‐Releasing Hormone in Rats,” Endocrinology 130, no. 2 (1992): 651–656. 1733713 10.1210/endo.130.2.1733713

[133] J. M. Rondeel , W. J. de Greef , and T. J. Visser , “Effect of Thyroid Status on Release of Hypothalamic Thyrotropin‐Releasing Hormone,” Hormone and Metabolic Research Supplement 23 (1990): 1–4. 2120121

[134] A. Rodríguez‐Rodríguez , I. Lazcano , E. Sánchez‐Jaramillo , et al., “Tanycytes and the Control of Thyrotropin‐Releasing Hormone Flux Into Portal Capillaries,” Frontiers in Endocrinology (Lausanne) 10 (2019): 401. 10.3389/fendo.2019.00401 PMC6603095 31293518

[135] K. Rizzoti and R. Lovell‐Badge , “Pivotal Role of Median Eminence Tanycytes for Hypothalamic Function and Neurogenesis,” Molecular and Cellular Endocrinology 445 (2017): 7–13. 27530416 10.1016/j.mce.2016.08.020

[136] E. Sánchez , M. A. Vargas , P. S. Singru , et al., “Tanycyte Pyroglutamyl Peptidase II Contributes to Regulation of the Hypothalamic‐Pituitary‐Thyroid axis Through Glial‐Axonal Associations in the Median Eminence,” Endocrinology 150, no. 5 (2009): 2283–2291. 19179432 10.1210/en.2008-1643 PMC2671897

[137] A. Marsili , E. Sanchez , P. Singru , et al., “Thyroxine‐Induced Expression of Pyroglutamyl Peptidase II and Inhibition of TSH Release Precedes Suppression of TRH mRNA and Requires Type 2 Deiodinase,” Journal of Endocrinology 211, no. 1 (2011): 73–78. 21788297 10.1530/JOE-11-0248 PMC3558748

[138] I. Lazcano , A. Cabral , R. M. Uribe , et al., “Fasting Enhances Pyroglutamyl Peptidase II Activity in Tanycytes of the Mediobasal Hypothalamus of Male Adult Rats,” Endocrinology 156, no. 7 (2015): 2713–2723. 25942072 10.1210/en.2014-1885

[139] R. M. Lechan , Y. Qi , T. J. Berrodin , et al., “Immunocytochemical Delineation of Thyroid Hormone Receptor beta 2‐Like Immunoreactivity in the Rat Central Nervous System,” Endocrinology 132, no. 6 (1993): 2461–2469. 7684976 10.1210/endo.132.6.7684976

[140] A. Chandrasekar , P. M. Schmidtlein , V. Neve , et al., “Regulation of Thyroid Hormone Gatekeepers by Thyrotropin in Tanycytes,” Thyroid 34 (2024): 261–273. 38115594 10.1089/thy.2023.0375

[141] Y. Sun , X. Lu , and M. C. Gershengorn , “Thyrotropin‐Releasing Hormone Receptors—Similarities and Differences,” Journal of Molecular Endocrinology 30, no. 2 (2003): 87–97. 12683933 10.1677/jme.0.0300087

[142] R. Rabeler , J. Mittag , L. Geffers , et al., “Generation of Thyrotropin‐Releasing Hormone Receptor 1‐Deficient Mice as an Animal Model of Central Hypothyroidism,” Molecular Endocrinology 18, no. 6 (2004): 1450–1460. 14988432 10.1210/me.2004-0017

[143] J. Cao , D. O'Donnell , H. Vu , et al., “Cloning and Characterization of a cDNA Encoding a Novel Subtype of Rat Thyrotropin‐Releasing Hormone Receptor,” Journal of Biological Chemistry 273, no. 48 (1998): 32281–32287. 9822707 10.1074/jbc.273.48.32281

[144] H. Heuer , M. K.‐H. Schäfer , D. O'Donnell , P. Walker , and K. Bauer , “Expression of Thyrotropin‐Releasing Hormone Receptor 2 (TRH‐R2) in the Central Nervous System of Rats,” Journal of Comparative Neurology 428, no. 2 (2000): 319–336. 11064370

[145] M. C. Gershengorn , “Mechanism of Signal Transduction by TRH,” Annals of the New York Academy of Sciences 553 (1989): 191–196. 2497671 10.1111/j.1749-6632.1989.tb46641.x

[146] R. Trubacova , Z. Drastichova , and J. Novotny , “Biochemical and Physiological Insights Into TRH Receptor‐Mediated Signaling,” Frontiers in Cell and Development Biology 10 (2022): 981452. 10.3389/fcell.2022.981452 PMC9485831 36147745

[147] L. Brenner‐Gati and M. C. Gershengorn , “Effects of Thyrotropin‐Releasing Hormone on Phosphoinositides and Cytoplasmic Free Calcium in Thyrotropic Pituitary Cells,” Endocrinology 118, no. 1 (1986): 163–169. 3000732 10.1210/endo-118-1-163

[148] F. E. Carr , R. J. Galloway , A. H. Reid , et al., “Thyrotropin‐Releasing Hormone Regulation of Thyrotropin beta‐Subunit Gene Expression Involves Intracellular Calcium and Protein Kinase C,” Biochemistry 30, no. 15 (1991): 3721–3728. 1707668 10.1021/bi00229a019

[149] F. E. Carr , C. U. Fisher , H. G. Fein , and R. C. Smallridge , “Thyrotropin‐Releasing Hormone Stimulates c‐Jun and c‐Fos Messenger Ribonucleic Acid Levels: Implications for Calcium Mobilization and Protein Kinase‐C Activation,” Endocrinology 133, no. 4 (1993): 1700–1707. 8404612 10.1210/endo.133.4.8404612

[150] M. A. Shupnik , J. Weck , and P. M. Hinkle , “Thyrotropin (TSH)‐Releasing Hormone Stimulates TSH Beta Promoter Activity by Two Distinct Mechanisms Involving Calcium Influx Through L Type Ca2+ Channels and Protein Kinase C,” Molecular Endocrinology (Baltimore, md) 10, no. 1 (1996): 90–99. 8838148 10.1210/mend.10.1.8838148

[151] B. Wang , J. Zhang , D. Zhang , et al., “Casein Kinase 1α as a Novel Factor Affects Thyrotropin Synthesis via PKC/ERK/CREB Signaling,” International Journal of Molecular Sciences 24, no. 8 (2023): 7034. 37108197 10.3390/ijms24087034 PMC10138882

[152] G. G. Altobelli , S. Van Noorden , D. Cimini , M. Illario , D. Sorriento , and V. Cimini , “Calcium/Calmodulin‐Dependent Kinases Can Regulate the TSH Expression in the Rat Pituitary,” Journal of Endocrinological Investigation 44, no. 11 (2021): 2387–2394. 33743173 10.1007/s40618-021-01545-0

[153] N. C. Vamvakopoulos and I. A. Kourides , “Identification of Separate mRNAs Coding for the Alpha and beta Subunits of Thyrotropin,” Proceedings of the National Academy of Sciences of the United States of America 76, no. 8 (1979): 3809–3813. 291041 10.1073/pnas.76.8.3809 PMC383924

[154] J. C. Fiddes and H. M. Goodman , “The Gene Encoding the Common Alpha Subunit of the Four Human Glycoprotein Hormones,” Journal of Molecular and Applied Genetics 1, no. 1 (1981): 3–18. 6286817

[155] M. Grossmann , B. D. Weintraub , and M. W. Szkudlinski , “Novel Insights Into the Molecular Mechanisms of Human Thyrotropin Action: Structural, Physiological, and Therapeutic Implications for the Glycoprotein Hormone Family,” Endocrine Reviews 18, no. 4 (1997): 476–501. 9267761 10.1210/edrv.18.4.0305

[156] M. A. Shupnik , S. L. Greenspan , and E. C. Ridgway , “Transcriptional Regulation of Thyrotropin Subunit Genes by Thyrotropin‐Releasing Hormone and Dopamine in Pituitary Cell Culture,” Journal of Biological Chemistry 261, no. 27 (1986): 12675–12679. 2427524

[157] D. J. Steger , J. H. Hecht , and P. L. Mellon , “GATA‐Binding Proteins Regulate the Human Gonadotropin Alpha‐Subunit Gene in the Placenta and Pituitary Gland,” Molecular and Cellular Biology 14, no. 8 (1994): 5592–5602. 7518566 10.1128/mcb.14.8.5592-5602.1994 PMC359078

[158] K. Ohba , S. Sasaki , A. Matsushita , et al., “GATA2 Mediates Thyrotropin‐Releasing Hormone‐Induced Transcriptional Activation of the Thyrotropin β Gene,” PLoS One 6, no. 4 (2011): e18667. 21533184 10.1371/journal.pone.0018667 PMC3077393

[159] D. S. Kim , S. K. Ahn , J. H. Yoon , et al., “Involvement of a cAMP‐Responsive DNA Element in Mediating TRH Responsiveness of the Human Thyrotropin Alpha‐Subunit Gene,” Molecular Endocrinology (Baltimore, md) 8, no. 4 (1994): 528–536. 7519724 10.1210/mend.8.4.7519724

[160] D. S. Kim , J. H. Yoon , S. K. Ahn , et al., “TRH Stimulation of Human Thyrotropin α‐Subunit Gene Expression Independent of de Novo Protein Synthesis,” Molecules and Cells 5, no. 3 (1995): 248–252.

[161] K. Hashimoto , K. Zanger , A. N. Hollenberg , L. E. Cohen , S. Radovick , and F. E. Wondisford , “cAMP Response Element‐Binding Protein‐Binding Protein Mediates Thyrotropin‐Releasing Hormone Signaling on Thyrotropin Subunit Genes,” Journal of Biological Chemistry 275, no. 43 (2000): 33365–33372. 10931853 10.1074/jbc.M006819200

[162] D. Wang , X. Xia , R. E. Weiss , S. Refetoff , and P. M. Yen , “Distinct and Histone‐Specific Modifications Mediate Positive Versus Negative Transcriptional Regulation of TSHalpha Promoter,” PLoS One 5, no. 3 (2010): e9853. 20352046 10.1371/journal.pone.0009853 PMC2844428

[163] M. Johannessen , M. P. Delghandi , and U. Moens , “What Turns CREB on?,” Cellular Signalling 16, no. 11 (2004): 1211–1227. 15337521 10.1016/j.cellsig.2004.05.001

[164] I. E. Akerblom , E. C. Ridgway , and P. L. Mellon , “An Alpha‐Subunit‐Secreting Cell Line Derived From a Mouse Thyrotrope Tumor,” Molecular Endocrinology 4, no. 4 (1990): 589–596. 1704102 10.1210/mend-4-4-589

[165] R. C. Fowkes , P. King , and J. M. Burrin , “Regulation of Human Glycoprotein Hormone Alpha‐Subunit Gene Transcription in LbetaT2 Gonadotropes by Protein Kinase C and Extracellular Signal‐Regulated Kinase 1/2,” Biology of Reproduction 67, no. 3 (2002): 725–734. 12193378 10.1095/biolreprod67.3.725

[166] K. Hashimoto , M. Yamada , T. Monden , T. Satoh , F. E. Wondisford , and M. Mori , “Thyrotropin‐Releasing Hormone (TRH) Specific Interaction Between Amino Terminus of P‐Lim and CREB Binding Protein (CBP),” Molecular and Cellular Endocrinology 229, no. 1–2 (2005): 11–20. 15607524 10.1016/j.mce.2004.10.005

[167] M. S. Roberson , W. E. Schoderbek , G. Tremml , and R. A. Maurer , “Activation of the Glycoprotein Hormone Alpha‐Subunit Promoter by a LIM‐Homeodomain Transcription Factor,” Molecular and Cellular Biology 14, no. 5 (1994): 2985–2993. 7513049 10.1128/mcb.14.5.2985-2993.1994 PMC358666

[168] D. F. Gordon , S. R. Lewis , B. R. Haugen , et al., “Pit‐1 and GATA‐2 Interact and Functionally Cooperate to Activate the Thyrotropin beta‐Subunit Promoter,” Journal of Biological Chemistry 272, no. 39 (1997): 24339–24347. 9305891 10.1074/jbc.272.39.24339

[169] B. R. Haugen , M. T. McDermott , D. F. Gordon , C. L. Rupp , W. M. Wood , and E. C. Ridgway , “Determinants of Thyrotrope‐Specific Thyrotropin β Promoter Activation: Cooperation of Pit‐1 With Another Factor (*),” Journal of Biological Chemistry 271, no. 1 (1996): 385–389. 8550592 10.1074/jbc.271.1.385

[170] H. J. Steinfelder , P. Hauser , Y. Nakayama , et al., “Thyrotropin‐Releasing Hormone Regulation of Human TSHB Expression: Role of a Pituitary‐Specific Transcription Factor (Pit‐1/GHF‐1) and Potential Interaction With a Thyroid Hormone‐Inhibitory Element,” Proceedings of the National Academy of Sciences of the United States of America 88, no. 8 (1991): 3130–3134. 1901656 10.1073/pnas.88.8.3130 PMC51399

[171] B. R. Haugen , D. F. Gordon , A. R. Nelson , W. M. Wood , and E. C. Ridgway , “The Combination of Pit‐1 and Pit‐1T Have a Synergistic Stimulatory Effect on the Thyrotropin beta‐Subunit Promoter but Not the Growth Hormone or Prolactin Promoters,” Molecular Endocrinology 8, no. 11 (1994): 1574–1582. 7877626 10.1210/mend.8.11.7877626

[172] H. J. Steinfelder , S. Radovick , and F. E. Wondisford , “Hormonal Regulation of the Thyrotropin Beta‐Subunit Gene by Phosphorylation of the Pituitary‐Specific Transcription Factor Pit‐1,” Proceedings of the National Academy of Sciences of the United States of America 89, no. 13 (1992): 5942–5945. 1321428 10.1073/pnas.89.13.5942 PMC402114

[173] K. Nakano , A. Matsushita , S. Sasaki , et al., “Thyroid‐Hormone‐Dependent Negative Regulation of Thyrotropin beta Gene by Thyroid Hormone Receptors: Study With a New Experimental System Using CV1 Cells,” Biochemical Journal 378, no. Pt 2 (2004): 549–557. 14611644 10.1042/BJ20031592 PMC1223958

[174] D. F. Gordon , E. A. Tucker , K. Tundwal , H. Hall , W. M. Wood , and E. C. Ridgway , “MED220/Thyroid Receptor‐Associated Protein 220 Functions as a Transcriptional Coactivator With Pit‐1 and GATA‐2 on the Thyrotropin‐Beta Promoter in Thyrotropes,” Molecular Endocrinology 20, no. 5 (2006): 1073–1089. 16396960 10.1210/me.2005-0115

[175] A. Matsushita , S. Sasaki , Y. Kashiwabara , et al., “Essential Role of GATA2 in the Negative Regulation of Thyrotropin beta Gene by Thyroid Hormone and Its Receptors,” Molecular Endocrinology 21, no. 4 (2007): 865–884. 17244762 10.1210/me.2006-0208

[176] K. Matsui , K. Oda , S. Mizuta , et al., “Mediator Subunit MED1 Is a T3‐Dependent and T3‐Independent Coactivator on the Thyrotropin β Gene Promoter,” Biochemical and Biophysical Research Communications 440, no. 1 (2013): 184–189. 24055033 10.1016/j.bbrc.2013.09.061 PMC4388257

[177] M. Ito , C. X. Yuan , H. J. Okano , R. B. Darnell , and R. G. Roeder , “Involvement of the TRAP220 Component of the TRAP/SMCC Coactivator Complex in Embryonic Development and Thyroid Hormone Action,” Molecular Cell 5, no. 4 (2000): 683–693. 10882104 10.1016/s1097-2765(00)80247-6

[178] Y. Kashiwabara , S. Sasaki , A. Matsushita , et al., “Functions of PIT1 in GATA2‐Dependent Transactivation of the Thyrotropin Beta Promoter,” Journal of Molecular Endocrinology 42, no. 3 (2009): 225–237. 19103719 10.1677/JME-08-0099

[179] Y. Nakajima , M. Yamada , R. Taguchi , et al., “NR4A1 (Nur77) Mediates Thyrotropin‐Releasing Hormone‐Induced Stimulation of Transcription of the Thyrotropin β Gene: Analysis of TRH Knockout Mice,” PLoS One 7, no. 7 (2012): e40437. 22792320 10.1371/journal.pone.0040437 PMC3392219

[180] K. K. Kim , S. B. Song , K. I. Kang , M. Rhee , and K. E. Kim , “Activation of the Thyroid‐Stimulating Hormone Beta‐Subunit Gene by LIM Homeodomain Transcription Factor Lhx2,” Endocrinology 148, no. 7 (2007): 3468–3476. 17446187 10.1210/en.2006-1088

[181] S. Ray , D. Dutta , M. A. Rumi , L. N. Kent , M. J. Soares , and S. Paul , “Context‐Dependent Function of Regulatory Elements and a Switch in Chromatin Occupancy Between GATA3 and GATA2 Regulate Gata2 Transcription During Trophoblast Differentiation,” Journal of Biological Chemistry 284, no. 8 (2009): 4978–4988. 19106099 10.1074/jbc.M807329200 PMC2643515

[182] M. Kobayashi‐Osaki , O. Ohneda , N. Suzuki , et al., “GATA Motifs Regulate Early Hematopoietic Lineage‐Specific Expression of the Gata2 Gene,” Molecular and Cellular Biology 25, no. 16 (2005): 7005–7020. 16055713 10.1128/MCB.25.16.7005-7020.2005 PMC1190224

[183] D. Nozawa , N. Suzuki , M. Kobayashi‐Osaki , X. Pan , J. D. Engel , and M. Yamamoto , “GATA2‐Dependent and Region‐Specific Regulation of Gata2 Transcription in the Mouse Midbrain,” Genes to Cells 14, no. 5 (2009): 569–582. 19371385 10.1111/j.1365-2443.2009.01289.x

[184] N. Hirahara , H. M. Nakamura , S. Sasaki , et al., “Liganded T3 Receptor β2 Inhibits the Positive Feedback Autoregulation of the Gene for GATA2, a Transcription Factor Critical for Thyrotropin Production,” PLoS One 15, no. 1 (2020): e0227646. 31940421 10.1371/journal.pone.0227646 PMC6961892

[185] M. W. Szkudlinski , V. Fremont , C. Ronin , and B. D. Weintraub , “Thyroid‐Stimulating Hormone and Thyroid‐Stimulating Hormone Receptor Structure‐Function Relationships,” Physiological Reviews 82, no. 2 (2002): 473–502. 11917095 10.1152/physrev.00031.2001

[186] M. T. Ribela , R. Damiani , F. D. Silva , et al., “N‐Glycoprofiling Analysis for Carbohydrate Composition and Site‐Occupancy Determination in a Poly‐Glycosylated Protein: Human Thyrotropin of Different Origins,” International Journal of Molecular Sciences 18, no. 2 (2017): 131. 28165356 10.3390/ijms18020131 PMC5343769

[187] T. Taylor and B. D. Weintraub , “Thyrotropin (TSH)‐releasing Hormone Regulation of TSH Subunit Biosynthesis and Glycosylation in Normal and Hypothyroid Rat Pituitaries,” Endocrinology 116, no. 5 (1985): 1968–1976. 3921348 10.1210/endo-116-5-1968

[188] B. D. Weintraub , N. Gesundheit , T. Taylor , and P. W. Gyves , “Effect of TRH on TSH Glycosylation and Biological Action,” Annals of the New York Academy of Sciences 553 (1989): 205–213. 2497672 10.1111/j.1749-6632.1989.tb46643.x

[189] R. W. Lash , R. K. Desai , C. A. Zimmerman , et al., “Mutations of the Human Thyrotropin‐ß Subunit Glycosylate Site Reduce Thyrotropin Synthesis Independent of Changes in Glycosylate Status,” Journal of Endocrinological Investigation 15, no. 4 (1992): 255–263. 1512415 10.1007/BF03348723

[190] N. R. Thotakura , M. W. Szkudlinski , and B. D. Weintraub , “Structure‐Function Studies of Oligosaccharides of Recombinant Human Thyrotrophin by Sequential Deglycosylation and Resialylation,” Glycobiology 4, no. 4 (1994): 525–533. 7827414 10.1093/glycob/4.4.525

[191] G. Ponsin and R. Mornex , “Control of Thyrotropin Glycosylation in Normal Rat Pituitary Cells in Culture: Effect of Thyrotropin‐Releasing Hormone,” Endocrinology 113, no. 2 (1983): 549–556. 6409587 10.1210/endo-113-2-549

[192] J. F. Wilber , “Stimulation of 14‐C‐Glucosamine and 14‐C‐Alanine Incorporation Into Thyrotropin by Synthetic Thyrotropin‐Releasing Hormone,” Endocrinology 89, no. 3 (1971): 873–877. 4998470 10.1210/endo-89-3-873

[193] C. Ronin , B. S. Stannard , and B. D. Weintraub , “Differential Processing and Regulation of Thyroid‐Stimulating Hormone Subunit Carbohydrate Chains in Thyrotropic Tumors and in Normal and Hypothyroid Pituitaries,” Biochemistry 24, no. 20 (1985): 5626–5631. 4074717 10.1021/bi00341a051

[194] B. D. Weintraub , B. S. Stannard , and L. Meyers , “Glycosylation of Thyroid‐Stimulating Hormone in Pituitary Tumor Cells: Influence of High Mannose Oligosaccharide Units on Subunit Aggregation, Combination, and Intracellular Degradation,” Endocrinology 112, no. 4 (1983): 1331–1345. 6403327 10.1210/endo-112-4-1331

[195] T. Taylor and B. D. Weintraub , “Altered Thyrotropin (TSH) Carbohydrate Structures in Hypothalamic Hypothyroidism Created by Paraventricular Nuclear Lesions Are Corrected by In Vivo TSH‐Releasing Hormone Administration,” Endocrinology 125, no. 4 (1989): 2198–2203. 2507289 10.1210/endo-125-4-2198

[196] B. M. Lifschitz , C. R. Defesi , and M. I. Surks , “Thyrotropin Response to Thyrotropin‐Releasing Hormone in the Euthyroid Rat: Dose‐Response, Time Course, and Demonstration of Partial Refractoriness to a Second Dose of Thyrotropin‐Releasing Hormone,” Endocrinology 102, no. 6 (1978): 1775–1782. 105883 10.1210/endo-102-6-1775

[197] L. Shenkman , T. Mitsuma , and C. S. Hollander , “Modulation of Pituitary Responsiveness to Thyrotropin‐Releasing Hormone by Triiodothyronine,” Journal of Clinical Investigation 52, no. 1 (1973): 205–209. 4629908 10.1172/JCI107166 PMC302244

[198] P. J. Snyder and R. D. Utiger , “Inhibition of Thyrotropin Response to Thyrotropin‐Releasing Hormone by Small Quantities of Thyroid Hormones,” Journal of Clinical Investigation 51, no. 8 (1972): 2077–2084. 4626582 10.1172/JCI107014 PMC292364

[199] M. Yamada , Y. Saga , N. Shibusawa , et al., “Tertiary Hypothyroidism and Hyperglycemia in Mice With Targeted Disruption of the Thyrotropin‐Releasing Hormone Gene,” Proceedings of the National Academy of Sciences of the United States of America 94, no. 20 (1997): 10862–10867. 9380725 10.1073/pnas.94.20.10862 PMC23510

[200] W. Vale , G. Grant , M. Amoss , R. Blackwell , and R. Guillemin , “Culture of Enzymatically Dispersed Anterior Pituitary Cells: Functional Validation of a Method,” Endocrinology 91, no. 2 (1972): 562–572. 4630101 10.1210/endo-91-2-562

[201] M. C. Gershengorn , “Regulation of Thyrotropin Production by Mouse Pituitary Thyrotropic Tumor Cells in Vitro by Physiological Levels of Thyroid Hormones,” Endocrinology 102, no. 4 (1978): 1122–1128. 744013 10.1210/endo-102-4-1122

[202] M. C. Gershengorn , “Bihormonal Regulation of the Thyrotropin‐Releasing Hormone Receptor in Mouse Pituitary Thyrotropic Tumor Cells in Culture,” Journal of Clinical Investigation 62, no. 5 (1978): 937–943. 213447 10.1172/JCI109222 PMC371851

[203] M. A. Christoffolete , R. Ribeiro , P. Singru , et al., “Atypical Expression of Type 2 Iodothyronine Deiodinase in Thyrotrophs Explains the Thyroxine‐Mediated Pituitary Thyrotropin Feedback Mechanism,” Endocrinology 147, no. 4 (2006): 1735–1743. 16396983 10.1210/en.2005-1300

[204] S. L. Abend , S. L. Fang , S. Alex , L. E. Braverman , and J. L. Leonard , “Rapid Alteration in Circulating Free Thyroxine Modulates Pituitary Type II 5′ Deiodinase and Basal Thyrotropin Secretion in the Rat,” Journal of Clinical Investigation 88, no. 3 (1991): 898–903. 1885776 10.1172/JCI115392 PMC295477

[205] S. W. Kim , J. W. Harney , and P. R. Larsen , “Studies of the Hormonal Regulation of Type 2 5′‐Iodothyronine Deiodinase Messenger Ribonucleic Acid in Pituitary Tumor Cells Using Semiquantitative Reverse Transcription‐Polymerase Chain Reaction,” Endocrinology 139, no. 12 (1998): 4895–4905. 9832426 10.1210/endo.139.12.6334

[206] A. Serrano‐Lozano , M. Montiel , M. Morell , and P. Morata , “5' Deiodinase Activity in Brain Regions of Adult Rats: Modifications in Different Situations of Experimental Hypothyroidism,” Brain Research Bulletin 30, no. 5–6 (1993): 611–616. 8457909 10.1016/0361-9230(93)90090-x

[207] A. Batistuzzo , F. Salas‐Lucia , B. Gereben , M. O. Ribeiro , and A. C. Bianco , “Sustained Pituitary T3 Production Explains the T4‐Mediated TSH Feedback Mechanism,” Endocrinology 164, no. 12 (2023): bqad155. 37864846 10.1210/endocr/bqad155 PMC10637099

[208] V. K. Chatterjee , J. K. Lee , A. Rentoumis , and J. L. Jameson , “Negative Regulation of the Thyroid‐Stimulating Hormone Alpha Gene by Thyroid Hormone: Receptor Interaction Adjacent to the TATA Box,” Proceedings of the National Academy of Sciences of the United States of America 86, no. 23 (1989): 9114–9118. 2480596 10.1073/pnas.86.23.9114 PMC298444

[209] J. Burnside , D. S. Darling , F. E. Carr , and W. W. Chin , “Thyroid Hormone Regulation of the Rat Glycoprotein Hormone Alpha‐Subunit Gene Promoter Activity,” Journal of Biological Chemistry 264, no. 12 (1989): 6886–6891. 2468663

[210] D. Wang , X. Xia , Y. Liu , et al., “Negative Regulation of TSHalpha Target Gene by Thyroid Hormone Involves Histone Acetylation and Corepressor Complex Dissociation,” Molecular Endocrinology (Baltimore, md) 23, no. 5 (2009): 600–609. 19196836 10.1210/me.2008-0389 PMC2675953

[211] F. E. Carr and W. W. Chin , “Differential Thyroid Hormone‐Regulated Rat Thyrotropin Beta Gene Expression Detected by Blot Hybridization,” Molecular Endocrinology 2, no. 8 (1988): 667–673. 3211152 10.1210/mend-2-8-667

[212] W. M. Wood , M. Y. Kao , D. F. Gordon , and E. C. Ridgway , “Thyroid Hormone Regulates the Mouse Thyrotropin Beta‐Subunit Gene Promoter in Transfected Primary Thyrotropes,” Journal of Biological Chemistry 264, no. 25 (1989): 14840–14847. 2768243

[213] W. W. Chin , M. A. Shupnik , D. S. Ross , J. F. Habener , and E. C. Ridgway , “Regulation of the Alpha and Thyrotropin Beta‐Subunit Messenger Ribonucleic Acids by Thyroid Hormones,” Endocrinology 116, no. 3 (1985): 873–878. 2578951 10.1210/endo-116-3-873

[214] F. E. Carr , J. Burnside , and W. W. Chin , “Thyroid Hormones Regulate Rat Thyrotropin Beta Gene Promoter Activity Expressed in GH3 Cells,” Molecular Endocrinology 3, no. 4 (1989): 709–716. 2542780 10.1210/mend-3-4-709

[215] F. E. Carr , L. L. Kaseem , and N. C. Wong , “Thyroid Hormone Inhibits Thyrotropin Gene Expression via a Position‐Independent Negative L‐Triiodothyronine‐Responsive Element,” Journal of Biological Chemistry 267, no. 26 (1992): 18689–18694. 1527000

[216] F. E. Carr and N. C. Wong , “Characteristics of a Negative Thyroid Hormone Response Element,” Journal of Biological Chemistry 269, no. 6 (1994): 4175–4179. 8307979

[217] F. E. Wondisford , E. A. Farr , S. Radovick , et al., “Thyroid Hormone Inhibition of Human Thyrotropin Beta‐Subunit Gene Expression Is Mediated by a cis‐Acting Element Located in the First Exon,” Journal of Biological Chemistry 264, no. 25 (1989): 14601–14604. 2768233

[218] D. L. Bodenner , M. A. Mroczynski , B. D. Weintraub , S. Radovick , and F. E. Wondisford , “A Detailed Functional and Structural Analysis of a Major Thyroid Hormone Inhibitory Element in the Human Thyrotropin Beta‐Subunit Gene,” Journal of Biological Chemistry 266, no. 32 (1991): 21666–21673. 1657975

[219] M. I. Chiamolera , A. R. Sidhaye , S. Matsumoto , et al., “Fundamentally Distinct Roles of Thyroid Hormone Receptor Isoforms in a Thyrotroph Cell Line Are due to Differential DNA Binding,” Molecular Endocrinology (Baltimore, md) 26, no. 6 (2012): 926–939. 22570333 10.1210/me.2011-1290 PMC3355539

[220] A. Fraichard , O. Chassande , M. Plateroti , et al., “The T3R Alpha Gene Encoding a Thyroid Hormone Receptor Is Essential for Post‐Natal Development and Thyroid Hormone Production,” EMBO Journal 16, no. 14 (1997): 4412–4420. 9250685 10.1093/emboj/16.14.4412 PMC1170067

[221] A. C. Figueira , I. Polikarpov , D. Veprintsev , and G. M. Santos , “Dissecting the Relation Between a Nuclear Receptor and GATA: Binding Affinity Studies of Thyroid Hormone Receptor and GATA2 on TSHβ Promoter,” PLoS One 5, no. 9 (2010): e12628. 20838640 10.1371/journal.pone.0012628 PMC2935386

[222] I. M. Krane , E. R. Spindel , and W. W. Chin , “Thyroid Hormone Decreases the Stability and the Poly(A) Tract Length of Rat Thyrotropin Beta‐Subunit Messenger RNA,” Molecular Endocrinology 5, no. 4 (1991): 469–475. 1922079 10.1210/mend-5-4-469

[223] J. M. Staton and P. J. Leedman , “Posttranscriptional Regulation of Thyrotropin Beta‐Subunit Messenger Ribonucleic Acid by Thyroid Hormone in Murine Thyrotrope Tumor Cells: A Conserved Mechanism Across Species,” Endocrinology 139, no. 3 (1998): 1093–1100. 9492042 10.1210/endo.139.3.5799

[224] F. Goulart‐Silva , P. B. de Souza , and M. T. Nunes , “T3 Rapidly Modulates TSHβ mRNA Stability and Translational Rate in the Pituitary of Hypothyroid Rats,” Molecular and Cellular Endocrinology 332, no. 1–2 (2011): 277–282. 21078364 10.1016/j.mce.2010.11.005

[225] P. J. Leedman , A. R. Stein , and W. W. Chin , “Regulated Specific Protein Binding to a Conserved Region of the 3′‐Untranslated Region of Thyrotropin Beta‐Subunit mRNA,” Molecular Endocrinology 9, no. 3 (1995): 375–387. 7776983 10.1210/mend.9.3.7776983

[226] J. M. Staton , A. M. Thomson , and P. J. Leedman , “Hormonal Regulation of mRNA Stability and RNA‐Protein Interactions in the Pituitary,” Journal of Molecular Endocrinology 25, no. 1 (2000): 17–34. 10915215 10.1677/jme.0.0250017

[227] Z. Wang , N. Day , P. Trifillis , and M. Kiledjian , “An mRNA Stability Complex Functions With Poly(A)‐Binding Protein to Stabilize mRNA In Vitro,” Molecular and Cellular Biology 19, no. 7 (1999): 4552–4560. 10373504 10.1128/mcb.19.7.4552 PMC84253

[228] P. M. Hinkle and K. B. Goh , “Regulation of Thyrotropin‐Releasing Hormone Receptors and Responses by L‐Triiodothyronine in Dispersed Rat Pituitary Cell Cultures,” Endocrinology 110, no. 5 (1982): 1725–1731. 6280973 10.1210/endo-110-5-1725

[229] M. Yamada , T. Monden , T. Satoh , et al., “Differential Regulation of Thyrotropin‐Releasing Hormone Receptor mRNA Levels by Thyroid Hormone In Vivo and In Vitro (GH3 Cells),” Biochemical and Biophysical Research Communications 184, no. 1 (1992): 367–372. 1373613 10.1016/0006-291x(92)91202-2

[230] L. Schomburg and K. Bauer , “Thyroid Hormones Rapidly and Stringently Regulate the Messenger RNA Levels of the Thyrotropin‐Releasing Hormone (TRH) Receptor and the TRH‐Degrading Ectoenzyme,” Endocrinology 136, no. 8 (1995): 3480–3485. 7628384 10.1210/endo.136.8.7628384

[231] J. Trojan , M. Theodoropoulou , K. H. Usadel , G. K. Stalla , and L. Schaaf , “Modulation of Human Thyrotropin Oligosaccharide Structures—Enhanced Proportion of Sialylated and Terminally Galactosylated Serum Thyrotropin Isoforms in Subclinical and Overt Primary Hypothyroidism,” Journal of Endocrinology 158, no. 3 (1998): 359–365. 9846165 10.1677/joe.0.1580359

[232] M. J. Papandreou , L. Persani , C. Asteria , C. Ronin , and P. Beck‐Peccoz , “Variable Carbohydrate Structures of Circulating Thyrotropin as Studied by Lectin Affinity Chromatography in Different Clinical Conditions,” Journal of Clinical Endocrinology and Metabolism 77, no. 2 (1993): 393–398. 8345043 10.1210/jcem.77.2.8345043

[233] L. Wide and K. Eriksson , “Thyrotropin N‐Glycosylation and Glycan Composition in Severe Primary Hypothyroidism,” Journal of the Endocrine Society 5, no. 4 (2021): bvab006. 33644618 10.1210/jendso/bvab006 PMC7896355

[234] L. Persani , S. Borgato , R. Romoli , C. Asteria , A. Pizzocaro , and P. Beck‐Peccoz , “Changes in the Degree of Sialylation of Carbohydrate Chains Modify the Biological Properties of Circulating Thyrotropin Isoforms in Various Physiological and Pathological States,” Journal of Clinical Endocrinology and Metabolism 83, no. 7 (1998): 2486–2492. 9661632 10.1210/jcem.83.7.4970

[235] M. W. Szkudlinski , N. R. Thotakura , J. E. Tropea , M. Grossmann , and B. D. Weintraub , “Asparagine‐Linked Oligosaccharide Structures Determine Clearance and Organ Distribution of Pituitary and Recombinant Thyrotropin,” Endocrinology 136, no. 8 (1995): 3325–3330. 7628367 10.1210/endo.136.8.7628367

[236] K. Bauer , P. Carmeliet , M. Schulz , M. Baes , and C. Denef , “Regulation and Cellular Localization of the Membrane‐Bound Thyrotropin‐Releasing Hormone‐Degrading Enzyme in Primary Cultures of Neuronal, Glial and Adenohypophyseal Cells,” Endocrinology 127, no. 3 (1990): 1224–1233. 2117525 10.1210/endo-127-3-1224

[237] G. Ponce , J. L. Charli , J. A. Pasten , C. Aceves , and P. Joseph‐Bravo , “Tissue‐Specific Regulation of Pyroglutamate Aminopeptidase II Activity by Thyroid Hormones,” Neuroendocrinology 48, no. 2 (1988): 211–213. 2906116 10.1159/000125011

[238] H. Heuer , J. Ehrchen , K. Bauer , and M. K. Schäfer , “Region‐Specific Expression of Thyrotrophin‐Releasing Hormone‐Degrading Ectoenzyme in the Rat Central Nervous System and Pituitary Gland,” European Journal of Neuroscience 10, no. 4 (1998): 1465–1478. 9749801 10.1046/j.1460-9568.1998.00158.x

[239] R. Cruz , M. A. Vargas , R. M. Uribe , et al., “Anterior Pituitary Pyroglutamyl Peptidase II Activity Controls TRH‐Induced Prolactin Release,” Peptides 29, no. 11 (2008): 1953–1964. 18703099 10.1016/j.peptides.2008.07.011

[240] M. A. Vargas , M. Cisneros , P. Joseph‐Bravo , and J. L. Charli , “Regulation of Adenohypophyseal Pyroglutamyl Aminopeptidase II Activity by Thyrotropin‐Releasing Hormone and Phorbol Esters,” Endocrine 13, no. 3 (2000): 267–272. 11216637 10.1385/endo:13:3:267

[241] A. C. M. Figueira , L. M. T. R. Lima , L. H. F. Lima , A. T. Ranzani , G. dos Santos Mule , and I. Polikarpov , “Recognition by the Thyroid Hormone Receptor of Canonical DNA Response Elements,” Biochemistry 49, no. 5 (2010): 893–904. 20025240 10.1021/bi901282s

[242] S. Ercan‐Fang , H. L. Schwartz , C. N. Mariash , and J. H. Oppenheimer , “Quantitative Assessment of Pituitary Resistance to Thyroid Hormone From Plots of the Logarithm of Thyrotropin Versus Serum Free Thyroxine Index,” Journal of Clinical Endocrinology and Metabolism 85, no. 6 (2000): 2299–2303. 10852467 10.1210/jcem.85.6.6625

[243] B. N. Kholodenko , J. B. Hoek , H. V. Westerhoff , and G. C. Brown , “Quantification of Information Transfer via Cellular Signal Transduction Pathways,” FEBS Letters 414, no. 2 (1997): 430–434. 9315734 10.1016/s0014-5793(97)01018-1

[244] R. M. Bassiri and R. D. Utiger , “Metabolism and Excretion of Exogenous Thyrotropin‐Releasing Hormone in Humans,” Journal of Clinical Investigation 52, no. 7 (1973): 1616–1619. 4198107 10.1172/JCI107339 PMC302433

[245] J. T. Nicoloff , J. C. Low , J. H. Dussault , and D. A. Fisher , “Simultaneous Measurement of Thyroxine and Triiodothyronine Peripheral Turnover Kinetics in Man,” Journal of Clinical Investigation 51, no. 3 (1972): 473–483. 4110897 10.1172/JCI106835 PMC302152

[246] W. D. Odell , R. D. Utiger , J. F. Wilber , and P. G. Condliffe , “Estimation of the Secretion Rate of Thyrotropin in Man,” Journal of Clinical Investigation 46, no. 6 (1967): 953–959. 6026100 10.1172/JCI105601 PMC297099

[247] B. Novák and J. J. Tyson , “Design Principles of Biochemical Oscillators,” Nature Reviews Molecular Cell Biology 9, no. 12 (2008): 981–991. 18971947 10.1038/nrm2530 PMC2796343

[248] S. P. Fitzgerald , N. G. Bean , and L. N. Fitzgerald , “Population Data Indicate That Thyroid Regulation Is Consistent With an Equilibrium‐Point Model, but Not With a Set‐Point Model,” Temperature (Austin) 4, no. 2 (2017): 114–116. 28680925 10.1080/23328940.2017.1281370 PMC5489013

[249] S. P. Fitzgerald and N. G. Bean , “Population Correlations Do Not Support the Existence of Set Points for Blood Levels of Calcium or Glucose—A New Model for Homeostasis,” Physiological Reports 6, no. 1 (2018): e13551. 29333728 10.14814/phy2.13551 PMC5789653

[250] P. B. Samollow , G. Perez , C. M. Kammerer , et al., “Genetic and Environmental Influences on Thyroid Hormone Variation in Mexican Americans,” Journal of Clinical Endocrinology and Metabolism 89, no. 7 (2004): 3276–3284. 15240603 10.1210/jc.2003-031706

[251] V. Panicker , S. G. Wilson , T. D. Spector , et al., “Heritability of Serum TSH, Free T4 and Free T3 Concentrations: A Study of a Large UK Twin Cohort,” Clinical Endocrinology 68, no. 4 (2008): 652–659. 17970774 10.1111/j.1365-2265.2007.03079.x

[252] J. Nolan , P. J. Campbell , S. J. Brown , et al., “Genome‐Wide Analysis of Thyroid Function in Australian Adolescents Highlights SERPINA7 and NCOA3,” European Journal of Endocrinology 185, no. 5 (2021): 743–753. 34524976 10.1530/EJE-21-0614

[253] J. Gudmundsson , P. Sulem , D. F. Gudbjartsson , et al., “Discovery of Common Variants Associated With Low TSH Levels and Thyroid cancer Risk,” Nature Genetics 44, no. 3 (2012): 319–322. 22267200 10.1038/ng.1046 PMC3655412

[254] E. Porcu , M. Medici , G. Pistis , et al., “A meta‐Analysis of Thyroid‐Related Traits Reveals Novel Loci and Gender‐Specific Differences in the Regulation of Thyroid Function,” PLoS Genetics 9, no. 2 (2013): e1003266. 23408906 10.1371/journal.pgen.1003266 PMC3567175

[255] M. Medici , W. M. van der Deure , M. Verbiest , et al., “A Large‐Scale Association Analysis of 68 Thyroid Hormone Pathway Genes With Serum TSH and FT4 Levels,” European Journal of Endocrinology 164, no. 5 (2011): 781–788. 21367965 10.1530/EJE-10-1130

[256] L. C. Moeller , M. Alonso , X. Liao , et al., “Pituitary‐Thyroid Setpoint and Thyrotropin Receptor Expression in Consomic Rats,” Endocrinology 148, no. 10 (2007): 4727–4733. 17640981 10.1210/en.2007-0236

[257] N. Lafontaine , P. J. Campbell , J. E. Castillo‐Fernandez , et al., “Epigenome‐Wide Association Study of Thyroid Function Traits Identifies Novel Associations of fT3 With KLF9 and DOT1L,” Journal of Clinical Endocrinology and Metabolism 106, no. 5 (2021): e2191–e2202. 33484127 10.1210/clinem/dgaa975 PMC8063248

[258] A. Chatzitomaris , R. Hoermann , J. E. Midgley , et al., “Thyroid Allostasis–Adaptive Responses of Thyrotropic Feedback Control to Conditions of Strain, Stress, and Developmental Programming,” Frontiers in Endocrinology 8 (2017): 163. 28775711 10.3389/fendo.2017.00163 PMC5517413

[259] E. Fliers , A. Kalsbeek , and A. Boelen , “Beyond the Fixed Setpoint of the Hypothalamus‐Pituitary‐Thyroid axis,” European Journal of Endocrinology 171, no. 5 (2014): R197–R208. 25005935 10.1530/EJE-14-0285

[260] P. Joseph‐Bravo , L. Jaimes‐Hoy , and J. L. Charli , “Advances in TRH Signaling,” Reviews in Endocrine & Metabolic Disorders 17, no. 4 (2016): 545–558. 27515033 10.1007/s11154-016-9375-y

[261] E. Mihály , C. Fekete , J. B. Tatro , Z. Liposits , E. G. Stopa , and R. M. Lechan , “Hypophysiotropic Thyrotropin‐Releasing Hormone‐Synthesizing Neurons in the Human Hypothalamus Are Innervated by Neuropeptide Y, agouti‐Related Protein, and Alpha‐Melanocyte‐Stimulating Hormone,” Journal of Clinical Endocrinology and Metabolism 85, no. 7 (2000): 2596–2603. 10902813 10.1210/jcem.85.7.6662

[262] B. Gereben , A. M. Zavacki , S. Ribich , et al., “Cellular and Molecular Basis of Deiodinase‐Regulated Thyroid Hormone Signaling,” Endocrine Reviews 29, no. 7 (2008): 898–938. 18815314 10.1210/er.2008-0019 PMC2647704

[263] D. Nevozhay , R. M. Adams , K. F. Murphy , K. Josić , and G. Balázsi , “Negative Autoregulation Linearizes the Dose–Response and Suppresses the Heterogeneity of Gene Expression,” Proceedings of the National Academy of Sciences 106, no. 13 (2009): 5123–5128. 10.1073/pnas.0809901106 PMC2654390 19279212

[264] O. E. Sturm , R. Orton , J. Grindlay , et al., “The Mammalian MAPK/ERK Pathway Exhibits Properties of a Negative Feedback Amplifier,” Science Signaling 3, no. 153 (2010): ra90. 21177493 10.1126/scisignal.2001212

[265] M. Khammash , “An Engineering Viewpoint on Biological Robustness,” BMC Biology 14, no. 1 (2016): 22. 27007299 10.1186/s12915-016-0241-x PMC4804522

[266] W. F. Ganong , “Circumventricular Organs: Definition and Role in the Regulation of Endocrine and Autonomic Function,” Clinical and Experimental Pharmacology & Physiology 27, no. 5–6 (2000): 422–427. 10831247 10.1046/j.1440-1681.2000.03259.x

[267] C. Kiecker , “The Origins of the Circumventricular Organs,” Journal of Anatomy 232, no. 4 (2018): 540–553. 29280147 10.1111/joa.12771 PMC5835788

[268] K. J. Åström and R. Murray , Feedback Systems: An Introduction for Scientists and Engineers (Princeton University Press, 2008).

[269] W. Ma , A. Trusina , H. El‐Samad , W. A. Lim , and C. Tang , “Defining Network Topologies That Can Achieve Biochemical Adaptation,” Cell 138, no. 4 (2009): 760–773. 19703401 10.1016/j.cell.2009.06.013 PMC3068210

[270] T. M. Yi , Y. Huang , M. I. Simon , and J. Doyle , “Robust Perfect Adaptation in Bacterial Chemotaxis Through Integral Feedback Control,” Proceedings of the National Academy of Sciences of the United States of America 97, no. 9 (2000): 4649–4653. 10781070 10.1073/pnas.97.9.4649 PMC18287

[271] D. Muzzey , C. A. Gómez‐Uribe , J. T. Mettetal , and A. van Oudenaarden , “A Systems‐Level Analysis of Perfect Adaptation in Yeast Osmoregulation,” Cell 138, no. 1 (2009): 160–171. 19596242 10.1016/j.cell.2009.04.047 PMC3109981

[272] S. K. Aoki , G. Lillacci , A. Gupta , A. Baumschlager , D. Schweingruber , and M. Khammash , “A Universal Biomolecular Integral Feedback Controller for Robust Perfect Adaptation,” Nature 570, no. 7762 (2019): 533–537. 31217585 10.1038/s41586-019-1321-1

[273] T. Frei , C.‐H. Chang , M. Filo , A. Arampatzis , and M. Khammash , “A Genetic Mammalian Proportional–Integral Feedback Control Circuit for Robust and Precise Gene Regulation,” Proceedings of the National Academy of Sciences 119, no. 24 (2022): e2122132119. 10.1073/pnas.2122132119 PMC9214505 35687671

[274] N. Atapattu , N. Shaw , and W. Högler , “Relationship Between Serum 25‐Hydroxyvitamin D and Parathyroid Hormone in the Search for a Biochemical Definition of Vitamin D Deficiency in Children,” Pediatric Research 74, no. 5 (2013): 552–556. 23999068 10.1038/pr.2013.139

[275] F. Malberti , M. Farina , and E. Imbasciati , “The PTH‐Calcium Curve and the Set Point of Calcium in Primary and Secondary Hyperparathyroidism,” Nephrology, Dialysis, Transplantation 14, no. 10 (1999): 2398–2406. 10.1093/ndt/14.10.2398 10528664

[276] G. P. Mayer and J. G. Hurst , “Sigmoidal Relationship Between Parathyroid Hormone Secretion Rate and Plasma Calcium Concentration in Calves,” Endocrinology 102, no. 4 (1978): 1036–1042. 744006 10.1210/endo-102-4-1036

[277] S. Goede , M. Leow , J. Smit , H. Klein , and J. W. Dietrich , “Hypothalamus‐Pituitary‐Thyroid Feedback Control: Implications of Mathematical Modeling and Consequences for Thyrotropin (TSH) and Free Thyroxine (FT4) Reference Ranges,” Bulletin of Mathematical Biology 76 (2014): 1270–1287. 24789568 10.1007/s11538-014-9955-5

[278] R. Hoermann , M. J. Pekker , J. E. M. Midgley , R. Larisch , and J. W. Dietrich , “Principles of Endocrine Regulation: Reconciling Tensions Between Robustness in Performance and Adaptation to Change,” Frontiers in Endocrinology 13 (2022): 825107. 35757421 10.3389/fendo.2022.825107 PMC9219553

[279] J. J. DiStefano, 3rd and E. B. Stear , “Neuroendocrine Control of Thyroid Secretion in Living Systems: A Feedback Control System Model,” Bulletin of Mathematical Biophysics 30, no. 1 (1968): 3–26. 4969955 10.1007/BF02476936

[280] J. Berberich , J. W. Dietrich , R. Hoermann , and M. A. Müller , “Mathematical Modeling of the Pituitary‐Thyroid Feedback Loop: Role of a TSH‐T(3)‐shunt and Sensitivity Analysis,” Frontiers in Endocrinology (Lausanne) 9 (2018): 91. 10.3389/fendo.2018.00091 PMC5871688 29619006

[281] J. W. Dietrich , G. Landgrafe , and E. H. Fotiadou , “TSH and Thyrotropic Agonists: Key Actors in Thyroid Homeostasis,” Journal of Thyroid Research 2012, no. 1 (2012): 351864. 23365787 10.1155/2012/351864 PMC3544290

[282] M. Eisenberg , M. Samuels , and J. J. DiStefano, 3rd , “Extensions, Validation, and Clinical Applications of a Feedback Control System Simulator of the Hypothalamo‐Pituitary‐Thyroid Axis,” Thyroid 18, no. 10 (2008): 1071–1085. 18844475 10.1089/thy.2007.0388 PMC2962855

[283] M. C. Eisenberg , F. Santini , A. Marsili , A. Pinchera , and J. J. DiStefano, 3rd , “TSH Regulation Dynamics in Central and Extreme Primary Hypothyroidism,” Thyroid 20, no. 11 (2010): 1215–1228. 21062194 10.1089/thy.2009.0349 PMC2974848

[284] R. Ben‐Shachar , M. Eisenberg , S. A. Huang , and J. J. DiStefano, 3rd , “Simulation of Post‐Thyroidectomy Treatment Alternatives for Triiodothyronine or Thyroxine Replacement in Pediatric Thyroid Cancer Patients,” Thyroid 22, no. 6 (2012): 595–603. 22578300 10.1089/thy.2011.0355 PMC3358124

[285] M. K.‐S. Leow , “A Mathematical Model of Pituitary–Thyroid Interaction to Provide an Insight Into the Nature of the Thyrotropin–Thyroid Hormone Relationship,” Journal of Theoretical Biology 248, no. 2 (2007): 275–287. 17602707 10.1016/j.jtbi.2007.05.016

[286] M. Pompa , A. De Gaetano , A. Borri , et al., “A Physiological Mathematical Model of the Human Thyroid,” Journal of Computational Science 76 (2024): 102236.

[287] S. X. Han , M. Eisenberg , P. R. Larsen , and J. DiStefano, 3rd , “THYROSIM App for Education and Research Predicts Potential Health Risks of Over‐The‐Counter Thyroid Supplements,” Thyroid 26, no. 4 (2016): 489–498. 26895744 10.1089/thy.2015.0373

[288] M. Cruz‐Loya , B. B. Chu , J. Jonklaas , D. F. Schneider , and J. DiStefano , “Optimized Replacement T4 and T4+T3 Dosing in Male and Female Hypothyroid Patients With Different BMIs Using a Personalized Mechanistic Model of Thyroid Hormone Regulation Dynamics,” Frontiers in Endocrinology 13 (2022): 888429. 35909562 10.3389/fendo.2022.888429 PMC9330449

[289] J. W. Dietrich , A. Tesche , C. R. Pickardt , and U. Mitzdorf , “Thyrotropic Feedback Control: Evidence for an Additional Ultrashort Feedback Loop From Fractal Analysis,” Cybernetics and Systems 35, no. 4 (2004): 315–331.

[290] J. E. Midgley , R. Hoermann , R. Larisch , and J. W. Dietrich , “Physiological States and Functional Relation Between Thyrotropin and Free Thyroxine in Thyroid Health and Disease: In Vivo and in Silico Data Suggest a Hierarchical Model,” Journal of Clinical Pathology 66, no. 4 (2013): 335–342. 23423518 10.1136/jclinpath-2012-201213

[291] T. M. Wolff , C. Veil , J. W. Dietrich , and M. A. Müller , “Mathematical Modeling and Simulation of Thyroid Homeostasis: Implications for the Allan‐Herndon‐Dudley Syndrome,” Frontiers in Endocrinology (Lausanne) 13 (2022): 882788. 10.3389/fendo.2022.882788 PMC9772020 36568087

[292] Y. Korem Kohanim , T. Milo , M. Raz , et al., “Dynamics of Thyroid Diseases and Thyroid‐Axis Gland Masses,” Molecular Systems Biology 18, no. 8 (2022): e10919. 35938225 10.15252/msb.202210919 PMC9358402

[293] M. Raz , D. S. Glass , T. Milo , et al., “Unifying Regulatory Motifs in Endocrine Circuits,” Nature Communications 16, no. 1 (2025): 11017. 10.1038/s41467-025-65924-4 PMC12695981 41271754

[294] P. M. Hinkle , A. U. Gehret , and B. W. Jones , “Desensitization, Trafficking, and Resensitization of the Pituitary Thyrotropin‐Releasing Hormone Receptor,” Frontiers in Neuroscience 6 (2012): 180. 23248581 10.3389/fnins.2012.00180 PMC3521152

[295] J. Fujimoto , C. S. Narayanan , J. E. Benjamin , and M. C. Gershengorn , “Posttranscriptional Up‐Regulation of Thyrotropin‐Releasing Hormone (TRH) Receptor Messenger Ribonucleic Acid by TRH in COS‐1 Cells Transfected With Mouse Pituitary TRH Receptor Complementary Deoxyribonucleic Acid,” Endocrinology 131, no. 4 (1992): 1716–1720. 1327718 10.1210/endo.131.4.1327718

[296] J. Fujimoto , R. E. Straub , and M. C. Gershengorn , “Thyrotropin‐Releasing Hormone (TRH) and Phorbol Myristate Acetate Decrease TRH Receptor Messenger RNA in Rat Pituitary GH3 Cells: Evidence That Protein Kinase‐C Mediates the TRH Effect,” Molecular Endocrinology (Baltimore, md) 5, no. 10 (1991): 1527–1532. 1723145 10.1210/mend-5-10-1527

[297] J. Yang and A. H. Tashjian, Jr. , “Regulation of Endogenous Thyrotropin‐Releasing Hormone (TRH) Receptor Messenger RNA by TRH in GH4C1 Cells,” Molecular Endocrinology 7, no. 6 (1993): 753–758. 8395652 10.1210/mend.7.6.8395652

[298] H. Müller‐Fielitz , M. Stahr , M. Bernau , et al., “Tanycytes Control the Hormonal Output of the Hypothalamic‐Pituitary‐Thyroid Axis,” Nature Communications 8, no. 1 (2017): 484. 10.1038/s41467-017-00604-6 PMC5589884 28883467

[299] E. Farkas , E. Varga , B. Kovács , et al., “A Glial‐Neuronal Circuit in the Median Eminence Regulates Thyrotropin‐Releasing Hormone‐Release via the Endocannabinoid System,” IScience 23, no. 3 (2020): 100921. 32143135 10.1016/j.isci.2020.100921 PMC7058404

[300] F. Roelfsema and J. D. Veldhuis , “Thyrotropin Secretion Patterns in Health and Disease,” Endocrine Reviews 34, no. 5 (2013): 619–657. 23575764 10.1210/er.2012-1076

[301] A. Guillou , Y. Kemkem , C. Lafont , et al., “TSH Pulses Finely Tune Thyroid Hormone Release and TSH Receptor Transduction,” Endocrinology 165, no. 1 (2024): 1–9. 10.1210/endocr/bqad164 PMC10666572 37934802

[302] M. F. Prummel , L. J. Brokken , and W. M. Wiersinga , “Ultra Short‐Loop Feedback Control of Thyrotropin Secretion,” Thyroid: Official Journal of the American Thyroid Association 14, no. 10 (2004): 825–829. 15588378 10.1089/thy.2004.14.825

[303] T. Kakita and W. D. Odell , “Pituitary Gland: One Site of Ultrashort‐Feedback Regulation for Control of Thyrotropin,” American Journal of Physiology 250, no. 2 Pt 1 (1986): E121–E124. 3082213 10.1152/ajpendo.1986.250.2.E121

[304] T. Kakita , N. P. Laborde , and W. D. Odell , “Autoregulatory Control of Thyrotropin in Rabbits,” Endocrinology 114, no. 6 (1984): 2301–2305. 6723584 10.1210/endo-114-6-2301

[305] M. F. Prummel , L. J. Brokken , G. Meduri , M. Misrahi , O. Bakker , and W. M. Wiersinga , “Expression of the Thyroid‐Stimulating Hormone Receptor in the Folliculo‐Stellate Cells of the Human Anterior Pituitary,” Journal of Clinical Endocrinology and Metabolism 85, no. 11 (2000): 4347–4353. 11095478 10.1210/jcem.85.11.6991

[306] L. J. Brokken , M. Leendertse , O. Bakker , W. M. Wiersinga , and M. F. Prummel , “Expression of Adenohypophyseal‐Hormone Receptors in a Murine Folliculo‐Stellate Cell Line,” Hormone and Metabolic Research 36, no. 8 (2004): 538–541. 15326563 10.1055/s-2004-825758

[307] L. J. Brokken , O. Bakker , W. M. Wiersinga , and M. F. Prummel , “Functional Thyrotropin Receptor Expression in the Pituitary Folliculo‐Stellate Cell Line TtT/GF,” Experimental and Clinical Endocrinology & Diabetes: Official Journal, German Society of Endocrinology [and] German Diabetes Association 113, no. 1 (2005): 13–20. 10.1055/s-2004-830516 15662590

[308] C. M. Dayan , P. Saravanan , and G. Bayly , “Whose Normal Thyroid Function Is Better—Yours or Mine?,” Lancet 360, no. 9330 (2002): 353–354. 12241772 10.1016/S0140-6736(02)09602-2

[309] V. H. Brun , A. H. Eriksen , R. Selseth , et al., “Patient‐Tailored Levothyroxine Dosage With Pharmacokinetic/Pharmacodynamic Modeling: A Novel Approach After Total Thyroidectomy,” Thyroid 31, no. 9 (2021): 1297–1304. 33980057 10.1089/thy.2021.0125 PMC8558060

[310] T. M. Wolff , J. W. Dietrich , and M. A. Müller , “Optimal Hormone Replacement Therapy in Hypothyroidism—A Model Predictive Control Approach,” Frontiers in Endocrinology 13 (2022): 884018. 35813623 10.3389/fendo.2022.884018 PMC9263720

[311] S. L. Goede , “Fast Track Treatment of Hypothyroidism With Levothyroxine: Reaching Homeostasis Within Four Weeks,” Acta Biotheoretica 71, no. 2 (2023): 10. 36881192 10.1007/s10441-023-09461-x

[312] F. Egalini , L. Marinelli , M. Rossi , et al., “Endocrine Disrupting Chemicals: Effects on Pituitary, Thyroid and Adrenal Glands,” Endocrine 78, no. 3 (2022): 395–405. 35604630 10.1007/s12020-022-03076-x PMC9637063

[313] B. Yilmaz , H. Terekeci , S. Sandal , and F. Kelestimur , “Endocrine Disrupting Chemicals: Exposure, Effects on Human Health, Mechanism of Action, Models for Testing and Strategies for Prevention,” Reviews in Endocrine & Metabolic Disorders 21, no. 1 (2020): 127–147. 31792807 10.1007/s11154-019-09521-z

[314] K. J. Oliveira , M. I. Chiamolera , G. Giannocco , C. C. Pazos‐Moura , and T. M. Ortiga‐Carvalho , “Thyroid Function Disruptors: From Nature to Chemicals,” Journal of Molecular Endocrinology 62 (2018): R1–R19. 10.1530/JME-18-0081 30006341

[315] L. Ramhøj , M. Axelstad , Y. Baert , et al., “New Approach Methods to Improve Human Health Risk Assessment of Thyroid Hormone System Disruption—A PARC Project,” Frontiers in Toxicology 5 (2023): 1189303. 37265663 10.3389/ftox.2023.1189303 PMC10229837

[316] P. D. Noyes , K. P. Friedman , P. Browne , et al., “Evaluating Chemicals for Thyroid Disruption: Opportunities and Challenges With in Vitro Testing and Adverse Outcome Pathway Approaches,” Environmental Health Perspectives 127, no. 9 (2019): e095001. 10.1289/EHP5297 PMC6791490 31487205

[317] N. Spinu , M. T. D. Cronin , S. J. Enoch , J. C. Madden , and A. P. Worth , “Quantitative Adverse Outcome Pathway (qAOP) Models for Toxicity Prediction,” Archives of Toxicology 94, no. 5 (2020): 1497–1510. 32424443 10.1007/s00204-020-02774-7 PMC7261727

[318] R. B. Conolly , G. T. Ankley , W. Cheng , et al., “Quantitative Adverse Outcome Pathways and Their Application to Predictive Toxicology,” Environmental Science & Technology 51, no. 8 (2017): 4661–4672. 28355063 10.1021/acs.est.6b06230 PMC6134852

[319] E. D. McLanahan , M. E. Andersen , J. L. Campbell , and J. W. Fisher , “Competitive Inhibition of Thyroidal Uptake of Dietary Iodide by Perchlorate Does Not Describe Perturbations in Rat Serum Total T 4 and TSH,” Environmental Health Perspectives 117, no. 5 (2009): 731–738. 19479014 10.1289/ehp.0800111 PMC2685834

[320] Z. Shi , S. Xiao , and Q. Zhang , “Interference With Systemic Negative Feedback as a Potential Mechanism for Nonmonotonic Dose‐Responses of Endocrine‐Disrupting Chemicals,” Toxicological Sciences 206, no. 2 (2025): 354–372. 40317127 10.1093/toxsci/kfaf060 PMC12342977

